# Sphingomyelin synthase-related protein is a regulator of serine palmitoyltransferase

**DOI:** 10.1016/j.jlr.2025.100908

**Published:** 2025-09-23

**Authors:** Xiang Li, Zhiqiang Li, Yeun-po Chiang, Tilla Worgall, Tade Souaiaia, Xian-Cheng Jiang

**Affiliations:** 1 Department of Cell Biology, State University of New York Downstate Health Sciences University, Brooklyn, NY, USA; 2 Molecular and Cellular Cardiology Program, VA New York Harbor Healthcare System, Brooklyn, NY, USA; 3 Department of Pathology, Columbia University, New York, NY, USA

**Keywords:** Sphingomyelin Synthase Related Protein, Phosphatidylethanolamine-specific phospholipase C, Serine Palmitoyltransferase, Phosphatidylethanolamine, Sphingolipid biosynthesis

## Abstract

Sphingomyelin synthase-related protein (SMSr) belongs to the SMS family; however, it cannot synthesize SM. We reported that SMSr is a phosphatidylethanolamine-specific phospholipase C, which is associated with metabolic dysfunction-associated fatty liver disease (MAFLD). However, the mechanism is unknown. Based on hierarchical clustering of the samples from the human Genotype-Tissue Expression project, we found that SMSr and serine palmitoyltransferase (SPT), the key enzyme for sphingolipid biosynthesis, as well as certain sphingolipid metabolism-related genes, belong to the same co-expression cluster in the liver and adipose tissues. We also found that *Smsr* expression is positively associated with *Sptlc1 and Sptlc2* expression in both tissues of both genders. In a mouse study, we found that *Smsr* overexpression induced while *Smsr* knockout (KO) (under a high-fat diet) reduced SPT activity, thus, influencing most of the tested sphingolipids. Further, we found that PE treatment reversed *Smsr* overexpression-mediated SPTLC2 upregulation. PE supplement also reduced liver microsome SPT activity in a dose-dependent manner. Furthermore, we demonstrated that SMSr interacts with SPTLC2 in vivo. Thus, SMSr, as a member in the sphingolipid biosynthesis pathway, regulates SPT. Perturbation of SPT activity has been linked to the prevention of MAFLD and cardiovascular diseases. However, the approach to finding an SPT-specific inhibitor, as a drug, has not been successful so far. Importantly, global *Smsr* KO mice are viable and healthy; therefore, inhibiting SPT activity by reducing PE, mediated by SMSr/PE-PLC activity, could provide a novel approach for preventing and treating MAFLD.

## Graphical Abstract

**Figure:**
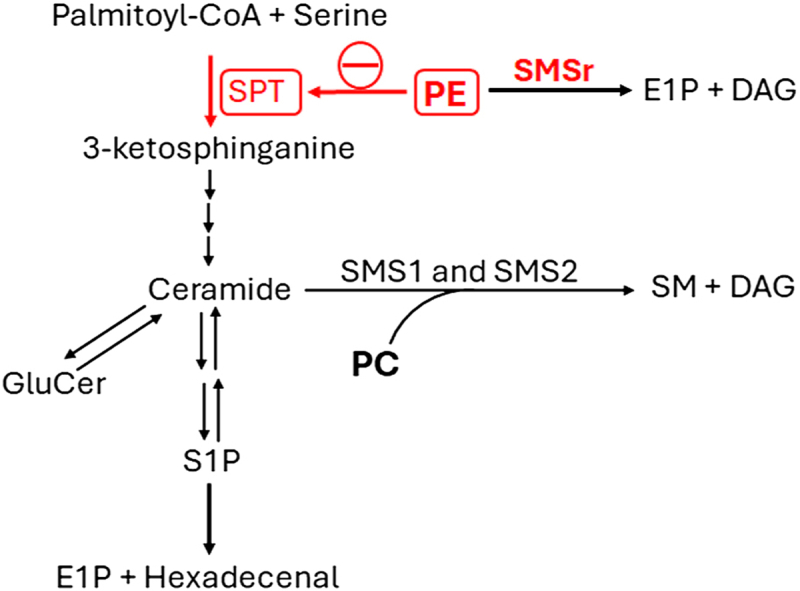

The sphingomyelin synthase (SMS) gene family includes SMS1, SMS2, and SMSr (1). SMSr, located on the endoplasmic reticulum (ER) and conserved in all animals, has no SM synthase activity (1). In vitro, SMSr uses the phosphatidylethanolamine (PE) and ceramide to produce ceramide phosphoethanolamine (CPE), an SM-like molecule (2, 3). Although *Smsr* is expressed ubiquitously (2), we and others have shown that CPE levels are extremely low in mouse liver and plasma (2, 3) and undetectable in most tissues. Mouse *Smsr* gene knockout (KO) (2, 3) or overexpression (4) strains show little effect on CPE; therefore, SMSr is not a CPE synthase in vivo. Recently, we and others found that SMSr has phospholipase C (PLC) activity (4, 5), and we also found that its PLC activity has PE specificity (4). Moreover, we found that SMSr/PE-PLC deficiency attenuates metabolic dysfunction-associated fatty liver disease (MAFLD) (6).

Sphingolipid biosynthesis in the ER starts with the condensation of serine and palmitoyl-CoA to form 3-ketosphinganine, catalyzed by serine palmitoyltransferase (SPT) (7). The mammalian SPT holoenzyme is primarily a heterodimer of two subunits, SPTLC1 and SPTLC2 (8, 9). Two additional low-molecular-weight proteins, ssSPTa and ssSPTb, enhance SPT activity and confer distinct acyl-CoA substrate specificities to mammalian SPT (10). Orosomucoid (ORM)1 and ORM2 that physically interact with SPTLC1 and negatively regulate SPT activity (11, 12). Human ORMDLs also negatively regulate SPT activity (13, 14). Recently, the structure of the SPT-ORMDL3 complex has been resolved by cryo-electron microscopy (EM) (15). In addition to ORMDLs, several other membrane-embedded proteins, including the neurite outgrowth inhibitor B (Nogo-B) are negative regulators of SPT (16).

Perturbation of SPT activity has been linked to metabolic diseases. Park *et al.* (17) and we (18) first reported that treatment with myriocin, a specific inhibitor of SPT activity, decreases atherosclerosis induced by a high-fat/high-cholesterol diet in mouse models. The mechanism could involve the reduction of plasma SM, ceramide, and glycosphingolipids. In mice, myriocin-mediated reduction of ceramide protects against diet-induced insulin resistance (19, 20), MALFD (21, 22). Heterozygous *Sptlc2* knockout (KO) mice were protected from high-fat-diet-induced obesity, insulin resistance (23) and atherosclerosis (24). All these observations clearly indicated that inhibiting SPT is an ideal approach for the treatment of metabolic diseases. However, a big challenge was met in the field. Although myriocin can help prevent metabolic diseases, it is toxic and has unfavorable physicochemical properties, such as low solubility (25). Thus, we need a new approach to regulate SPT activity.

We unexpectedly found that *Smsr* overexpression in mice induced most of the tested sphingolipids, whereas *Smsr* deficiency had the opposite effect under a high-fat diet. We then measured SPT activity and found that the overexpression induced while the deficiency reduced the activity. Importantly, global *Smsr* deficiency is different from SPT deficiency;. *Smsr* KO mice are viable, fertile, and healthy. Thus, the current study was designed to determine whether SMSr-mediated PE-PLC activity serves as a switch to fine-tune SPT activity.

## Materials and Methods

### Mice

In this study, all animal experiments were conducted with the approval of the Institutional Animal Care and Use Committee (IACUC) at SUNY Downstate Health Sciences University. The strategy to prepare global SMSr KO mice was previously described (2). Mice were fed either a standard normal chow diet (CD) (Cat#5053; PicoLab Rodent Diet) or a high-fat diet (HFD) (Envigo, Cat# TD88137) for 16 weeks. All mice were bred on a C57BL/6J genetic background.

### Adenovirus (AdV) – SMSr Administration

AdV-SMSr-FLAG and Ad-null were prepared by ViraQuest Inc. AdV-null or AdV-SMSr-FLAG (1 × 10^11^ viral particles/mouse) were injected intravenously (i.v.) into male wild-type (WT) C57BL/6J mice at the same age (2–3 months old). AdV-null served as the control. On day 4 after the injection, mouse livers and plasma were collected after 4-h fasting and stored at −80°C for future experiments.

### Quantitative reverse transcription PCR

Total RNA was extracted from the liver using TRIzol (Thermo Fisher Scientific), following the manufacturer's instructions. Total RNA (2 μg) was used with the High-Capacity cDNA Reverse Transcription Kit (Applied Biosystems). Quantitative PCR was performed by Real-Time PCR System using the SYBR Green Master Mix System (Applied Biosystems). Relative gene expression was normalized to GAPDH and calculated using the ΔΔCt method. The primer sequences used for real-time PCR are listed in supplemental Table S2.

### Measurement of PE-PLC activity

Liver microsomes were subjected to hydrolyze NBD-PE, generating NBD-DAG, which was used to measure the PE-PLC activity as previously discussed (6).

### PE measurement

PE was measured using the Phosphatidylethanolamine Assay Kit (Fluorometric) (Biovision, Cat# K499, according to the manufacturer’s protocols.

### Sphingolipids analyses

Sphingolipid levels in liver and plasma were measured using liquid chromatography with tandem mass spectrometry (LC-MS/MS) as described earlier (26).

### Western blot analysis and immunoprecipitation

Tissue homogenates were subjected to Western blotting as previously described (27). The following primary antibodies were used: Sptlc2 (Invitrogen, Cat# PA5-21142), Sptlc1, (BD transduction lab, Cat# 611304), FLAG (Sigma, Cat# A8592), Rabbit IgG (Cell signaling, Cat# 3900), Mouse IgG (Cell signaling, Cat# 68860), Glyceraldehyde-3-phosphate dehydrogenase (GAPDH) (Novus Biologicals, Cat# NB 300–324) and Beta-actin (Abcam, Cat# 9227). β-actin and GAPDH were used as loading controls.

For immunoprecipitation, tissues were lysed in a buffer containing 50 mM Tris-HCl (pH 7.4), 150 mM NaCl and 1% Triton X-100, supplemented with protease inhibitors. Primary antibodies (2–3 μg) were incubated with protein A/G agarose beads (Santa Cruz, Cat# sc-2003) overnight at 4°C. Pre-cleared tissue lysates were added to the antibody-coated beads and incubated for one hour at 4°C. Beads were washed and prepared to do immunoblotting.

### SPT activity measurement

Livers were homogenized in SPT homogenization buffer (10 mM HEPES, pH 7.5, 10 mM Sucrose, 1 mM EDTA) and centrifuged at 4,500 rpm at 4°C for 15 min. The supernatant was adjusted to a final concentration of 0.25 M sucrose, then spun at 50,000 rpm (SW55) for 1 hour at 4°C. The microsome pellets were resuspended in SPT microsome suspension buffer (10 mM HEPES, pH 7.5, 0.25 M Sucrose) for the SPT activity assay. ^14^C serine and palmitoyl-CoA, as substrates, were incubated with microsomes similarly as described previously (28). Following adding chloroform: methanol (v/v, 2/1), the organic phase was collected after centrifugation at 6,000×g for 10 min and washed twice with deionized water. The lipids were then dried under nitrogen gas, dissolved in 20 μl of chloroform, and counted using a liquid scintillation counter.

For the PE regulation of SPT activity experiment, PE (Avanti, Cat# 840026P) was dissolved in 100% ethanol. Liver microsomes (400 μg) were preincubated at 37°C for 20 min, then, PE (100 μM, 200 μM, and 300 μM) was added in a total volume of 100 μl. The mixture was then incubated with substrates for the SPT activity as discussed.

### PE treatment

AdV-SMSr-FLAG was administered to WT male mice on day 1. Starting the following day, mice were divided into two groups and received PE (20 μg/g body weight per day) daily intraperitoneal (i.p.) injections for the next seven days. The control group received vehicle injections, after the treatment, the mice were sacrificed, and liver tissues were collected and stored at −80°C for further analysis.

### RNA sequencing (RNA-seq)

Fresh frozen liver samples, isolated from AdV-null and AdV-SMSr-FLAG mice (n = 4 per group, male), were sent to Azenta Life Sciences Company for RNA extraction, library preparation, and standard RNA sequencing.

### RNA sequencing data processing and differential expression analysis

Sequence reads were trimmed to remove possible adapter sequences and nucleotides with poor quality using Trimmomatic v.0.36. The trimmed reads were mapped to the Mus musculus GRCm38 reference genome available on ENSEMBL using the STAR aligner v.2.5.2 b, which detects splice junctions and incorporates them to help align the entire read sequences. Unique gene hit counts were calculated by using featureCounts from the Subread package v.1.5.2, summarizing and reporting using the gene id feature in the annotation file. Only unique reads within exon regions were counted. If a strand-specific library preparation was performed, the reads were strand-specifically counted. After extraction of gene hit counts, the gene hit counts table was used for downstream differential expression analysis. Using DESeq2, a comparison of gene expression between the Ad-null and Ad-SMSr groups was performed. The Wald test was used to generate *P*-values and log2 fold changes. Genes with an adjusted *P*-value < 0.05 and absolute log2 fold change > 1 were considered differentially expressed genes (DEGs). Significantly differentially expressed genes were clustered by their gene ontology, and the enrichment of gene ontology terms was tested using Fisher exact test (GeneSCF v1.1-p2).

### Quantification and statistical analysis

Experimental analysis was carried out using GraphPad Prism, Version 8.0.2. Each in vitro experiment was independently performed with duplicate or triplicate to ensure reproducibility. Data are shown as mean ± SD. Unpaired two-tailed Student’s *t* test or Mann–Whitney U test was performed for two group analyses. Multiple group comparisons were tested by Kruskal–Wallis followed by Dunn post hoc multiple comparisons tests or Mann–Whitney corrected for multiple tests. *P* values of 0.05 or less were considered to be statistically significant.

Further statistical analysis and visualization of SC-RNA clusters were carried out using Python3. Groupwise comparison of tissue specific expression was accomplished using the regression tools included in the statsmodels library and hierarchical clustering was accomplished using Scipy; clustering relied on average linkage and a distance of one minus the absolute value of the Pearson correlation coefficient between genes.

## Results

### The association between *Smsr* expression with *Sptlc1/2* expression in human liver and adipose tissues

**Fig. 1:**
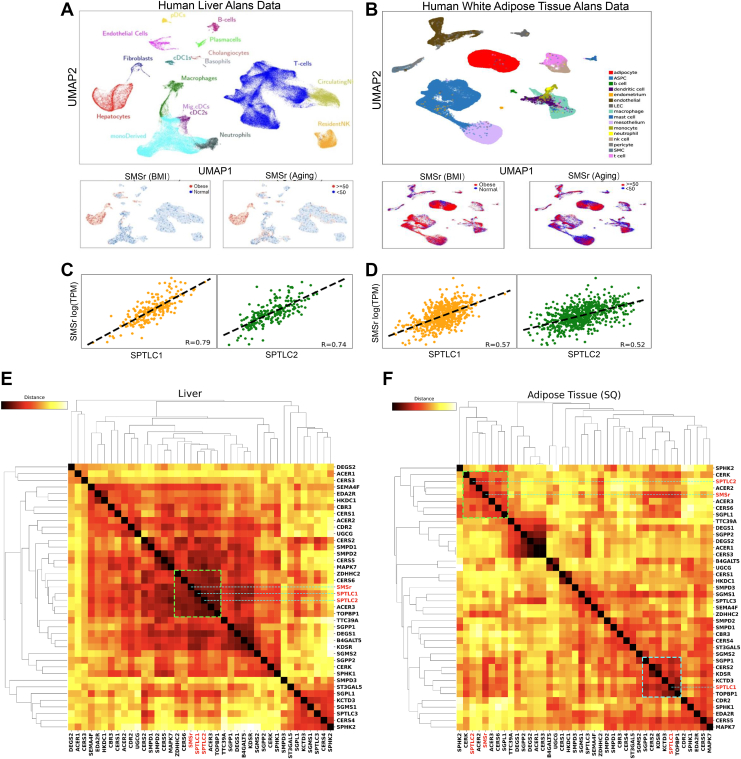
The Association of Human SMSr and SPT. A: Umap Analysis. Top Panel: Cell-Type Clusters from Human Liver Atlas. Bottom Panels: *Smsr* expression is significantly higher in obese and aged human samples, respectively. B: Human *Smsr* expression was positively associated with *Sptlc1* and *Sptlc2* expressions. The results were based on the analysis of the samples from GTEx project. Both male and female were included. C and D: heatmap of and hierarchical clustering of sphingolipid synthesis related genes in human liver and adipose tissue. Clustering was performed using average linkage and a distance of one minus the absolute value of the Pearson correlation coefficient between the two genes. Darker color indicates higher correlation. *Smsr* assigned to a group including *Sptlc1, Sptlc2, Cerk, Acer3, and Cers6* (green dot line highlighted in the middle) which are involved in sphingolipid synthesis, in the liver. *Smsr* assigned to a group including *Sptlc2, Sphk2, Cerk, Acer2,Acer3, and Cers6* (green dot line highlighted in the top left); which are involved in sphingolipid synthesis, in adipose tissues.

To analyze the role that SMSr plays in mammalian tissues and whether its role is conserved between humans and mice, we analyzed multiple publicly available human and mouse RNA-seq databases. An initial exploration of the publicly available database analyses (supplemental Table S2) revealed that SMSr upregulation is associated with age in humans and mice, with obesity in humans, and with a western diet in mice. Single-cell RNA-seq data allow specific cell-type clusters to be identified in a single tissue. Using Liver Atlas (29, 30) scRNA cell assignments (to 17unique clusters), we separated the cells assigned to hepatocytes by obesity status (BMI > 35) and aged status (>50 years) and found that both obese and aged group have greater *Smsr* expression in hepatocytes (*P* < 0.001). Using White Adipose tissue Atlas scRNA cell assignments (31), we obtained similar results (Fig. 1B).

To investigate the role of SMSr in the sphingolipid synthesis pathway in human tissues, as a member of the SM synthase family, we analyzed two tissue types from the GTEx database where the SM pathway is especially important: the liver and adipose tissue. Using a multiple regression model, we residualized gene expression values (log(TPM)). We found that *Smsr* is tightly correlated with *Sptlc1* and *Sptlc2* in the liver (Fig. 1C) and moderately correlated with both in the adipose tissue (Fig. 1D). To further investigate the association among *Smsr, Sptlc1, Sptlc2* and other genes in the sphingolipid synthesis pathway, we performed hierarchical clustering using the 30 sphingolipid biosynthesis genes and the ten genes with highest association with *Smsr*. Assigning genes to a group (shown in dotted line in Fig. 1E, F), based on a cutoff clustering distance of 0.6, we found that in the liver, *Smsr* and *Sptlc1* and *Sptlc2* are part of an expression cluster that also includes *Zdhhc2*, *Cers6, Acer3, and Topbp1* (Fig. 1e). In adipose tissue we found that *Sptlc2* but not *Sptlc1* was part of the same cluster, along with *Cerk, Acer2, Acer3, Cers6, and Sgpl1* (Fig. 1F). The Pearson correlation coefficient, Spearman rank, and SERE statistics were listed in supplemental Table S3. This suggests that although SMSr cannot synthesize SM, it could participate in sphingolipid metabolism through SPT regulation. Our preliminary analysis in bulk and pseudo-bulk single cell RNA sequencing (scRNA) data (combined across samples) also revealed that *Smsr* expression in the liver and is associated with obesity, age, gender, and diet (supplemental Table S2).

### *Smsr* overexpression in mice induces sphingolipid levels

**Fig. 2:**
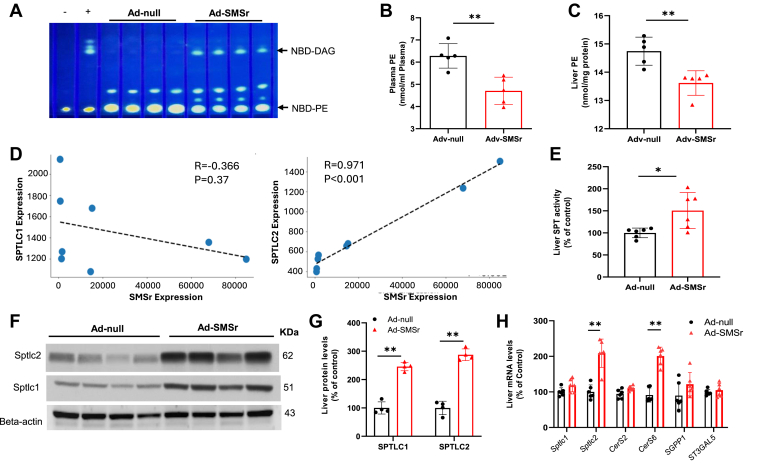
The association of mouse *Smsr* overexpression and SPT. Mice were injected (i.v.) with AdV-SMSr or AdV-null. Four days after the injection, the mouse liver was isolated. A: liver PE-PLC activity were measured. Bacterial PEPLC- was used as a positive control. B: plasma and (C) liver PE levels were measured. Mouse livers were used for RNA-Seq analysis. D: The correlation between *Smsr* and *Sptlc1* and *Sptlc2* expression across the samples. E: SPT activity was measured. F and G: Western blot and quantification of SPTLC1 and SPTLC2. H: Real-time PCR analysis for *Sptlc1/2* and other candidate genes which associate with SMSr.

We very recently found that SMSr is the first mammalian PE-PLC, biochemically (4) and structurally (32). To further characterize SMSr’s PE-PLC activity in vivo, we prepared adenovirus which express SMSr with a Flag tag (AdV-SMSr-Flag). We injected AdV-SMSr-Flag or AdV-null into WT mice and measured enzyme activity in liver microsomes 4 days after injection. AdV-SMSr-Flag treatment significantly increased liver PE-PLC activity (Fig. 2A) and significantly reduced plasma and liver PE (Kit, MAK361, Millipore Sigma) (Fig. 2B, C). CPE was not detected (measured by LC/MS/MS; data not shown).

While SMSr is associated with both SPTLC1 and SPTLC2 in human liver and adipose tissues, and clusters with SPTLC1 and SPTLC2 in the liver and with SPTLC2 but not SPTLC1 in the adipose tissue, the directionality of the relationship cannot easily be assessed with observational human RNA-seq data. In our previous work, using *Smsr* knockout (KO) mice and RNA-seq analysis, we found *Smsr* KO cause a reduction of *Sptlc2* but not *Sptlc1* expression (6). Here, based on RNA-seq analysis, we observed that the correlation between *Smsr* and *Sptlc2* expression across the samples is almost perfect, while *Smsr* and *Sptlc1* expression have no positive correlation (Fig. 2D). This suggests SMSr is likely a direct regulator of SPTLC2, which in turn modulates SPT activity.

Importantly, AdV-SMSr-FLAG expression in mice significantly increased SPT activity (Fig. 2E). Immunoblot analysis confirmed that both SPTLC1 and SPTL2 protein levels were elevated following *Smsr* overexpression (Fig. 2F, G). Given these findings, we sought to identify a set of candidate genes that exhibited a similar expression pattern in both our mouse model and the human RNA-seq data. Among the candidate genes analyzed, only *CERS6* was also found in both clusters (supplemental Table S3). To further examine this relationship, we measured mRNA levels of SPTLC1 and SPTLC2 and found that AdV-SMSr significantly induced *Sptlc2* mRNA but not *Sptlc1* mRNA (Fig. 2H). We also analyzed mRNA levels of other candidate genes in supplemental Table S4 and observed that *CerS6* was markedly upregulated (Fig. 2H). CerS6 is a key downstream enzyme of SPT, catalyzing the production of long-chain ceramides, particularly C18-ceramide. Thus, these results further support the idea that SPTLC2 could be the direct target of SMSr activity.

**Fig. 3:**
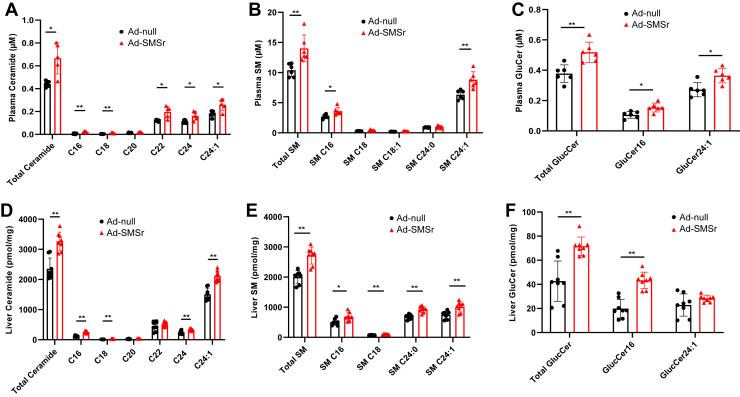
Sphingolipid measurement in *Smsr* Overexpressed and control mice. Sphingolipids were measured by LC/MS/MS. A–C: Plasma sphingolipids; (D–F) Liver sphingolipids. C, ceramide; SM, sphingomyelin; GluCer, glucosylceramide. Values are mean ± SD, n = 4–5, ∗*P* < 0.05; ∗∗*P* < 0.01.

We next measured sphingolipids in the liver and plasma by LC/MS/MS. We found that total ceramides (Fig. 3A, D), SMs (Fig. 3B, E), and GluCers (Fig. 3C, F) were all significantly increased, reflecting the fact that *Smsr* expression mediated the increase in sphingolipids through upregulation of SPT activity. While most major species were elevated, a few minor species showed little or no change. We also measured plasma triglyceride, total phospholipid, and total cholesterol levels in these mice, and we did not observe significant changes (supplemental Fig. S1).

### *Smsr* deficiency in mice reduces the tested sphingolipid levels under a high-fat diet

We previously reported that germline *Smsr*-KO mice show no significant changes in plasma sphingolipid levels compared with WT mice under a standard chow diet (2). Consistent with this, we confirmed that *Smsr* KO mouse livers exhibit comparable SPTLC1 and SPTLC2 protein levels (supplemental Fig. S2A, B) and mRNA expression (supplemental Fig. S2C), compared with controls. Also, there were no significant changes in the tested sphingolipid species, including ceramides, SMs, and GluCers (supplemental Fig. S2D–F).

**Fig. 4:**
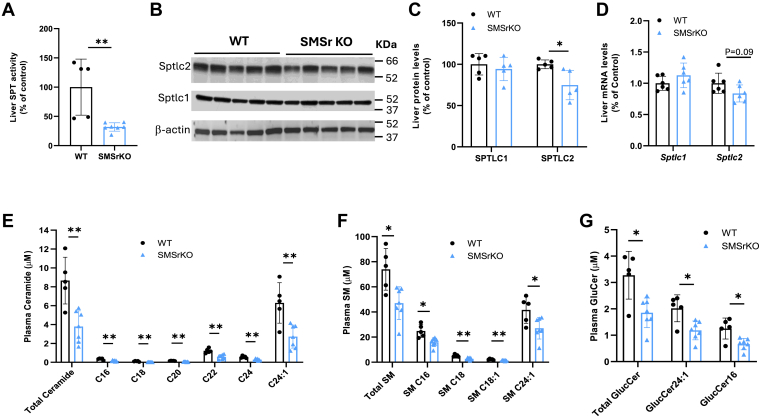
*Smsr* deficiency lowers liver SPTLC2 protein and SPT activity levels as well as a plasma sphingolipid levels under a high fat/cholesterol diet. Five-month-old *Smsr* KO and WT male mice were fed a high fat/cholesterol diet for 4 months. Their livers and plasma were isolated. A: Liver SPT activity was measured. B, C: Western blots and corresponding quantification of liver SPTLC1 and SPTLC2, normalized to the loading control (β-actin). D: Real-time PCR analysis for liver SPTLC1 and SPTLC2 mRNA. E-G: Plasma sphingolipid were measured by LC/MS/MS. Sphingolipid: C, ceramide; SM, sphingomyelin; GluCer, glucosylceramide. Values are mean ± SD, n = 4–7, ∗*P* < 0.05; ∗∗*P* < 0.01.

Next, we sought to challenge the KO mice with a high-fat/cholesterol diet for 4 months weeks and found that liver SPT activity was significantly reduced (Fig. 4A). Moreover, we found that SPTLC2 but not SPTLC1 protein mass (Fig. 4B, C) was significantly reduced in the liver, compared with the controls. However, the mRNA (Fig. 4D) levels of both *Sptlc1* and *Sptlc2* remain unchanged, indicating a post-translational regulation. Importantly, we observed a significant reduction in plasma ceramides, SMs, and GluCers (Fig. 4E–G), suggesting that *Smsr* deficiency reduces sphingolipid accumulation under metabolic stress and may protect against diet-induced sphingolipid elevation.

### PE treatment reduces liver SPT activity

**Fig. 5:**
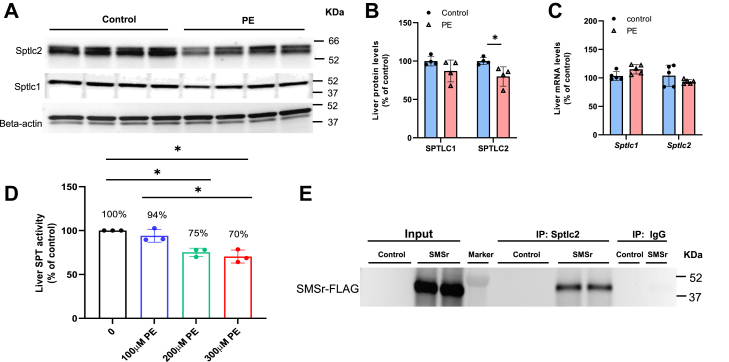
The effect of PE on SPT. Mice (three-month-old) were treated with AdV-SMSr, followed by 7 days of PE (i.p., 10 mg/kg) or vehicle injection. Their livers were isolated. A, B: Western blots and corresponding quantification of SPTLC1 and SPTLC2. C: Real-time PCR analysis for liver SPTLC1 and SPTLC2 mRNAs. D: Measurement of liver (AdV-SMSr-FLAG treated) microsome SPT activity by incubating 0 μM–300 μM liver PE with microsomes prior to substrate additing. E: Western blot analysis of FLAG-tagged SMSr coimmunoprecipitated with SPTLC2 (IP: SPTLC2) from mouse liver (AdV-SMSr-FLAG treated) lysates. Rabbit IgG was used as a negative control for immunoprecipitation. WT mice were intravenously injected with either AdV-null or AdV-SMSr-FLAG. Values are mean ± SD, n = 3–4, ∗*P* < 0.05; ∗∗*P* < 0.01.

To evaluate the direct effect of PE on SPT, we set up an in vivo approach. The *Smsr* overexpressed (AdV-SMSr-FLAG) mice were treated daily with PE (i.p., 10 mg/kg BW/day) or vehicle for 7 consecutive days. We expect that PE treatment will reverse the effect of *Smsr* overexpression-mediated up-regulation of SPTLC2. Indeed, immunoblotting analysis revealed that SPTLC2 but not SPTLC1 was significantly reduced in the PE-treated group compared with control (vehicle) (Fig. 5A, B). Whereas no changes were observed in mRNA levels of both SPTLC1 and SPTLC2 (Fig. 5C), indicating that the effect of PE occurs at the post-transcriptional level.

We next set up an in vitro approach to examine the effect of PE on SPT. We isolated liver microsomes from AdV-SMSr-FLAG mice and supplemented them with different concentrations of PE. We then measured SPT activity. We found that PE supplementation significantly reduced SPT activity in a dose-dependent fashion (Fig. 5D). Collectively, PE supplementation, which mimics the situation of *Smsr* deficiency, could reverse the *Smsr* expression-mediated induction of SPTLC2 and then SPT activity.

To evaluate a possible protein-protein interaction between SMSr and SPTLC2, we performed immunoprecipitation. We utilized an anti-SPTLC2 antibody to immunoprecipitated the potential complex from AdV-SMSr-FLAG treated mouse liver, and we then utilized an anti-Flag antibody to perform immunoblotting to detect SMSr-FLAG in the complex. We found that SPTLC2 co-immunoprecipitated with SMSr-Flag (Fig. 5E), indicating an in vivo interaction between SMSr and SPTLC2.

## Discussion

SMSr is conserved in animals and is ubiquitous in mammalian tissues (2). Despite being older than the mammalian-specific synthase genes, SMS1 and SMS2, its role has been poorly understood until recently. We found that SMSr is the first mammalian PE-PLC that uses PE to produce phosphoethanolamine (4) and resolved its cryo-EM structure which provided further evidence for its PE-PLC activity (32). We also found that PE-PLC deficiency attenuates diet-induced MAFLD (6). However, the mechanism is still unknown.

Using publicly available human data, we observed that obese and aged humans exhibit higher levels of SMSr mRNA in hepatocytes compared to normal and young humans (both male and female), respectively (Fig. 1A). Most metabolic diseases are associated with aging (33, 34), and our findings support that SMSr plays a role in the development of metabolic diseases. Further, we utilized results from human genome-wide association study (GWAS) and found three SMSr SNAPs (rs9299525, rs10762577, and rs6480771) that are associated with coronary artery disease, plasma triglyceride, and HDL-cholesterol (35, 36). In animal studies, SMSr expression has been linked to macrophage foam cell formation (37) and saturated fatty acid levels (38). Furthermore, through the analysis of publicly available RNA-seq data (39), we found that SMSr expression is significantly altered in mice under Western diet and aging conditions. Since SMSr is a component in sphingolipid biosynthesis pathway but cannot synthesize SM and CPE in vivo (2, 3), we aimed to investigate the mechanisms underlying these observations within the pathway.

We identified a strong co-relationship between human SMSr and SPT. We utilized existing human databases and found that there is a positive correlation with *Sptlc1/Sptlc2* expression in human liver and adipose tissues (Fig. 1B). Further, we found that *Smsr* and *Sptlc2*, together with other sphingolipid biosynthesis genes, belong to the same co-expression cluster in human liver and adipose tissues (Fig. 1C, D). Taken together, these human results suggest that SMSr plays a critical role in the development of metabolic diseases and this could be mediated by SMSr’s regulation of SPT.

Compelling evidence has shown that blocking SPT activity with myriocin (an SPT-specific inhibitor) decreases ceramide, SM, and glucosylceramide (GluCer) levels and attenuates MAFLD and insulin resistance (19, 20, 21, 22). All these observations clearly indicated that inhibiting SPT is an ideal approach for the treatment of metabolic diseases. However, a major challenge remains in the field. Although myriocin, can help prevent metabolic diseases, it is toxic (40) and has unfavorable physicochemical properties such as low solubility (25). A modification of myriocin led to the development of FTY720, an agonist of the S1P receptor rather than an SPT inhibitor, to treat multiple sclerosis (25). Thus, investigating a new way to regulate SPT activity could provide a novel approach for the treatment of MAFLD, insulin resistance, and atherosclerosis.

SPT is primarily a heterodimer of two subunits, SPTLC1 and SPTLC2 (8, 9). Recently, the structure of the SPT-ORMDL3 complex has been resolved by cryo-electron microscopy (EM) (15). Based on the structure, SPTLC1 is not directly involved in the catalytic reaction; rather, it acts as an anchor that targets SPTLC2 to the endoplasmic reticulum (ER) membrane (41). The fact that expressing SPTLC2 but not SPTLC1 in HeLa293 cells increases SPT activity (42) indicates that SPTLC2 is the rate determined factor for SPT activity.

SMSr is a positive regulator of SPT. Sphingolipids are essential for life, and germline SPT deficiency causes embryo lethality (28). Thus, sphingolipid biosynthesis must be regulated very precisely. There are two known internal feedback factors in the pathway, which can regulate SPT activity. 1) Ceramide can negatively regulate human SPT for establishing sphingolipid homeostasis (43); and 2) S1P lyase deficiency-mediated accumulation of S1P can down-regulate SPT activity (44), for maintaining sphingolipid homeostasis. In the current study, we found that *Smsr* overexpression induces while its deficiency reduces SPT activity. Thus, SMSr is a positive internal factor to fine-tune SPT activity for keeping sphingolipid homeostasis, although it cannot produce SM and CPE in vivo (2, 3).

The effect of SMSr on SPT is through its effect on SPTLC2. Based on *Smsr* overexpression and deficiency results, the most consistent association in the sphingolipid biosynthesis pathway is between SMSr and SPTLC2. We found that *Smsr* deficiency significantly reduces liver SPT activity and SPTLC2 but not SPTLC1 protein levels, and then all tested sphingolipid levels in the circulation (Fig. 3A–F). Immunoprecipitation clearly indicated that SMSr interacts with SPTLC2 (Fig. 5E). Since both SMSr (1, 45) and SPTLC2 (8, 9) are located in the ER and SMSr is complexed with SPTLC2, potentially, SMSr’s PE-PLC activity may participates in regulating SPT activity.

SMSr-mediated PE-PLC activity regulates the steady-state of SPT activity. We found that overexpression of SMSr significantly induces liver PE-PLC activity (Fig. 2A), reduces plasma and liver PE levels (Fig. 2B, C), and induces liver SPT activity (Fig. 2E). PE supplements in *Smsr* overexpressed mice significantly reduced SPTLC2 protein levels (Fig. 5A) and PE supplementation in liver microsomes, which overexpress *Smsr*, significantly reduces SPT activity in a dose-dependent manner (Fig. 5D). Although the exact mechanism remains to be further determined, it is possible that PE, like ceramide, could inhibit SPT activity by blocking the catalytic domain of the SPT–ORMDL3 complex (15). It is also possible that PE’s effect, like S1P, could be through regulating ORMDLs, which are negative regulators of SPT activity (13, 44). In a previous study, it has been shown that neurite outgrowth inhibitor B (Nogo-B) is also a negative regulator of SPT (16). It is also possible that PE can be a regulator of Nogo-B. Alternatively, changes in PE may alter membrane biophysical properties, such as curvature and fluidity (46), thereby influencing SPT-ORMDL3 complex stability. Moreover, elevated PE may trigger protein quality-control or degradation pathways (47). These possible mechanisms deserve further investigation.

SMSr-mediated PE-PLC activity can be one of the factors for controlling the steady-state of PE in vivo. PE (the second most abundant phospholipid in mammalian tissues) levels are determined by biosynthesis, through the Kennedy pathway in the ER and catabolism. Catabolism of PE in the ER can include 1) phosphatidylcholine (PC) formation via PE *N*-methyltansferase (48) and 2) hydrolysis by PE-PLC, as we discovered (4), which affects PE levels in the liver and circulation (4). PE and PC are two important lipid components of VLDL and its metabolite, LDL. Increasing PE or PE/PC ratio on VLDL and LDL contributes to the reduction of atherosclerosis in mouse models (49).

Previously, we reported that SMSr/PE-PLC activity also influences diacylglycerol (DAG) levels (6) which may, in turn, affect SPT regulation. DAG is known as an activator of certain protein kinases (PKCs) (50), however, DAG generated from different sources may have variable effects on PKCs. For example, DAG derived from the hydrolysis of cell membrane PI can activate PKCs, whereas DAG produced from other sources cannot activate PKCs (51). The impact of PE-PLC-related DAG changes deserves further investigation.

There is a limitation of the current study. Depletion of SMSr in the embryo may lead to compensatory responses; thus, results of the global approach may reflect these compensations together with the loss of SMSr. This could be the reason why the effect of overexpression of SMSr (in adulthood) is overwhelming, while the effect of Smsr deficiency (in early life) is rather modest and requires certain conditions, such as a high-fat diet.

In summary, we found that SMSr/PE-PLC is an SPT regulator, although it cannot directly be involved in SM biosynthesis in vivo. *Smsr* deficiency attenuates diet-induced obesity and MAFLD, which are reminiscent of SPTLC2 partial deficiency (23). It has been noted that, unlike germline SPT deficiency, germline SMSr-deficient mice are viable, fertile, and healthy. Thus, inhibiting SPT activity through reducing SMSr could provide an effective and novel approach for preventing and treating MAFLD, dyslipidemia, insulin resistance, and atherosclerosis in humans.

## Data Availability

All study data are included in the article and/or supplemental data.

## Supplemental data

This article contains supplemental data(30, 39, 52, 53).

## Conflict of interest

The authors declare that they do not have any conflicts of interest with the content of this article.

## References

[1] Huitema K., van den Dikkenberg J., Brouwers J.F., Holthuis J.C. (2004). Identification of a family of animal sphingomyelin synthases. EMBO J..

[2] Ding T., Kabir I., Li Y., Lou C., Yazdanyar A., Xu J. (2015). All members in the sphingomyelin synthase gene family have ceramide phosphoethanolamine synthase activity. J. Lipid Res..

[3] Bickert A., Ginkel C., Kol M., vom Dorp K., Jastrow H., Degen J. (2015). Functional characterization of enzymes catalyzing ceramide phosphoethanolamine biosynthesis in mice. J. Lipid Res..

[4] Chiang Y.P., Li Z., Chen Y., Cao Y., Jiang X.C. (2021). Sphingomyelin synthase related protein is a mammalian phosphatidylethanolamine phospholipase C. Biochim. Biophys. Acta Mol. Cell Biol. Lipids.

[5] Murakami C., Sakane F. (2021). Sphingomyelin synthase-related protein generates diacylglycerol via the hydrolysis of glycerophospholipids in the absence of ceramide. J. Biol. Chem..

[6] Chiang Y.P., Li Z., He M., Jones Q., Pan M., Han X. (2023). Sphingomyelin synthase-related protein SMSr is a phosphatidylethanolamine phospholipase C that promotes nonalcoholic fatty liver disease. J. Biol. Chem..

[7] Hannun Y.A., Obeid L.M. (2008). Principles of bioactive lipid signalling: lessons from sphingolipids. Nat. Rev. Mol. Cell Biol..

[8] Weiss B., Stoffel W. (1997). Human and murine serine-palmitoyl-coa transferase--cloning, expression and characterization of the key enzyme in sphingolipid synthesis. Eur. J. Biochem..

[9] Hanada K., Hara T., Nishijima M. (2000). Purification of the serine palmitoyltransferase complex responsible for sphingoid base synthesis by using affinity peptide chromatography techniques. J. Biol. Chem..

[10] Han G., Gupta S.D., Gable K., Niranjanakumari S., Moitra P., Eichler F. (2009). Identification of small subunits of mammalian serine palmitoyltransferase that confer distinct acyl-CoA substrate specificities. Proc. Natl. Acad. Sci. U. S. A..

[11] Breslow D.K., Collins S.R., Bodenmiller B., Aebersold R., Simons K., Shevchenko A. (2010). Orm family proteins mediate sphingolipid homeostasis. Nature.

[12] Han S., Lone M.A., Schneiter R., Chang A. (2010). Orm1 and Orm2 are conserved endoplasmic reticulum membrane proteins regulating lipid homeostasis and protein quality control. Proc. Natl. Acad. Sci. U. S. A..

[13] Siow D., Sunkara M., Dunn T.M., Morris A.J., Wattenberg B. (2015). ORMDL/serine palmitoyltransferase stoichiometry determines effects of ORMDL3 expression on sphingolipid biosynthesis. J. Lipid Res..

[14] Kiefer K., Carreras-Sureda A., Garcia-Lopez R., Rubio-Moscardo F., Casas J., Fabrias G. (2015). Coordinated regulation of the orosomucoid-like gene family expression controls de novo ceramide synthesis in mammalian cells. J. Biol. Chem..

[15] Li S., Xie T., Liu P., Wang L., Gong X. (2021). Structural insights into the assembly and substrate selectivity of human SPT-ORMDL3 complex. Nat. Struct. Mol. Biol..

[16] Cantalupo A., Zhang Y., Kothiya M., Galvani S., Obinata H., Bucci M. (2015). Nogo-B regulates endothelial sphingolipid homeostasis to control vascular function and blood pressure. Nat. Med..

[17] Park T.S., Panek R.L., Mueller S.B., Hanselman J.C., Rosebury W.S., Robertson A.W. (2004). Inhibition of sphingomyelin synthesis reduces atherogenesis in apolipoprotein E-knockout mice. Circulation.

[18] Hojjati M.R., Li Z., Zhou H., Tang S., Huan C., Ooi E. (2005). Effect of myriocin on plasma sphingolipid metabolism and atherosclerosis in apoE-deficient mice. J. Biol. Chem..

[19] Kurek K., Miklosz A., Lukaszuk B., Chabowski A., Gorski J., Zendzian-Piotrowska M. (2015). Inhibition of Ceramide De Novo Synthesis Ameliorates Diet Induced Skeletal Muscles Insulin Resistance. J. Diabetes Res..

[20] Holland W.L., Brozinick J.T., Wang L.P., Hawkins E.D., Sargent K.M., Liu Y. (2007). Inhibition of ceramide synthesis ameliorates glucocorticoid-, saturated-fat-, and obesity-induced insulin resistance. Cell Metab..

[21] Kasumov T., Li L., Li M., Gulshan K., Kirwan J.P., Liu X. (2015). Ceramide as a mediator of non-alcoholic Fatty liver disease and associated atherosclerosis. PLoS One.

[22] Kurek K., Piotrowska D.M., Wiesiolek-Kurek P., Lukaszuk B., Chabowski A., Gorski J. (2014). Inhibition of ceramide de novo synthesis reduces liver lipid accumulation in rats with nonalcoholic fatty liver disease. Liver Int. official J. Int. Assoc. Study Liver.

[23] Li Z., Zhang H., Liu J., Liang C.P., Li Y., Li Y. (2011). Reducing plasma membrane sphingomyelin increases insulin sensitivity. Mol. Cell Biol..

[24] Chakraborty M., Lou C., Huan C., Kuo M.S., Park T.S., Cao G. (2013). Myeloid cell-specific serine palmitoyltransferase subunit 2 haploinsufficiency reduces murine atherosclerosis. J. Clin. Invest..

[25] Adachi K., Chiba K. (2007). FTY720 story. Its discovery and the following accelerated development of sphingosine 1-phosphate receptor agonists as immunomodulators based on reverse pharmacology. Perspect. Med. Chem..

[26] Li Z., Ding T., Pan X., Li Y., Li R., Sanders P.E. (2012). Lysophosphatidylcholine acyltransferase 3 knockdown-mediated liver lysophosphatidylcholine accumulation promotes very low density lipoprotein production by enhancing microsomal triglyceride transfer protein expression. J. Biol. Chem..

[27] Li Z., Jiang H., Ding T., Lou C., Bui H.H., Kuo M.S. (2015). Deficiency in lysophosphatidylcholine acyltransferase 3 reduces plasma levels of lipids by reducing lipid absorption in mice. Gastroenterology.

[28] Hojjati M.R., Li Z., Jiang X.C. (2005). Serine palmitoyl-CoA transferase (SPT) deficiency and sphingolipid levels in mice. Biochim. Biophys. Acta.

[29] All of Us Research Program I, Denny J.C., Rutter J.L., Goldstein D.B., Philippakis A., Smoller J.W. (2019). The "All of us" research program. N. Engl. J. Med..

[30] Guilliams M., Bonnardel J., Haest B., Vanderborght B., Wagner C., Remmerie A. (2022). Spatial proteogenomics reveals distinct and evolutionarily conserved hepatic macrophage niches. Cell.

[31] Emont M.P., Jacobs C., Essene A.L., Pant D., Tenen D., Colleluori G. (2022). A single-cell atlas of human and mouse white adipose tissue. Nature.

[32] Hu K., Zhang Q., Chen Y., Yang J., Xia Y., Rao B. (2024). Cryo-EM structure of human sphingomyelin synthase and its mechanistic implications for sphingomyelin synthesis. Nat. Struct. Mol. Biol..

[33] Bonomini F., Rodella L.F., Rezzani R. (2015). Metabolic syndrome, aging and involvement of oxidative stress. Aging Dis..

[34] Amorim J.A., Coppotelli G., Rolo A.P., Palmeira C.M., Ross J.M., Sinclair D.A. (2022). Mitochondrial and metabolic dysfunction in ageing and age-related diseases. Nat. Rev. Endocrinol..

[35] Bellomo T.R., Bone W.P., Chen B.Y., Gawronski K.A.B., Zhang D., Park J. (2021). Multi-trait genome-wide association study of atherosclerosis detects novel pleiotropic loci. Front. Genet..

[36] Graham S.E., Clarke S.L., Wu K-H.H., Kanoni S., Zajac G.J.M., Ramdas S. (2021). The power of genetic diversity in genome-wide association studies of lipids. Nature.

[37] Seo J.W., Park K.S., Lee G.B., Park S.E., Choi J.H., Moon M.H. (2023). Comprehensive lipid profiling recapitulates enhanced lipolysis and fatty acid metabolism in intimal foamy macrophages from murine atherosclerotic aorta. Immune Netw..

[38] Laghouaouta H., Sosa-Madrid B.S., Zubiri-Gaitan A., Hernandez P., Blasco A. (2020). Novel genomic regions associated with intramuscular fatty acid composition in rabbits. Animals (Basel).

[39] Xiao Y., Batmanov K., Hu W., Zhu K., Tom A.Y., Guan D. (2023). Hepatocytes demarcated by EphB2 contribute to the progression of nonalcoholic steatohepatitis. Sci. Transl. Med..

[40] Johnson V.J., He Q., Osuchowski M.F., Sharma R.P. (2004). Disruption of sphingolipid homeostasis by myriocin, a mycotoxin, reduces thymic and splenic T-lymphocyte populations. Toxicology.

[41] Yasuda S., Nishijima M., Hanada K. (2003). Localization, topology, and function of the LCB1 subunit of serine palmitoyltransferase in mammalian cells. J. Biol. Chem..

[42] Hornemann T., Richard S., Rutti M.F., Wei Y., von Eckardstein A. (2006). Cloning and initial characterization of a new subunit for mammalian serine-palmitoyltransferase. J. Biol. Chem..

[43] Xie T., Liu P., Wu X., Dong F., Zhang Z., Yue J. (2023). Ceramide sensing by human SPT-ORMDL complex for establishing sphingolipid homeostasis. Nat. Commun..

[44] Hagen-Euteneuer N., Lutjohann D., Park H., Merrill A.H., van Echten-Deckert G. (2012). Sphingosine 1-phosphate (S1P) lyase deficiency increases sphingolipid formation via recycling at the expense of de novo biosynthesis in neurons. J. Biol. Chem..

[45] Tafesse F.G., Ternes P., Holthuis J.C. (2006). The multigenic sphingomyelin synthase family. J. Biol. Chem..

[46] van Meer G., Voelker D.R., Feigenson G.W. (2008). Membrane lipids: where they are and how they behave. Nat. Rev. Mol. Cell Biol..

[47] Doroudgar S., Völkers M., Thuerauf D.J., Khan M., Mohsin S., Respress J.L. (2015). Hrd1 and ER-Associated protein degradation, ERAD, are critical elements of the adaptive ER stress response in cardiac myocytes. Circ. Res..

[48] Walkey C.J., Donohue L.R., Bronson R., Agellon L.B., Vance D.E. (1997). Disruption of the murine gene encoding phosphatidylethanolamine N-methyltransferase. Proc. Natl. Acad. Sci. U. S. A..

[49] Zhao Y., Su B., Jacobs R.L., Kennedy B., Francis G.A., Waddington E. (2009). Lack of phosphatidylethanolamine N-methyltransferase alters plasma VLDL phospholipids and attenuates atherosclerosis in mice. Arterioscler. Thromb. Vasc. Biol..

[50] Huang K.P. (1989). The mechanism of protein kinase C activation. Trends Neurosci..

[51] Pettitt T.R., Martin A., Horton T., Liossis C., Lord J.M., Wakelam M.J. (1997). Diacylglycerol and phosphatidate generated by phospholipases C and D, respectively, have distinct fatty acid compositions and functions. Phospholipase D-derived diacylglycerol does not activate protein kinase C in porcine aortic endothelial cells. J. Biol. Chem..

[52] Scott C., Guilliams M. (2023). Liver cell atlas.

[53] Remmerie A., Martens L., Thoné T., Castoldi A., Seurinck R., Pavie B. (2020). Osteopontin expression identifies a subset of recruited macrophages distinct from kupffer cells in the fatty liver. Immunity.

