# Supplementary material for: Sphingomyelin synthase-related protein is a regulator of serine palmitoyltransferase
Source: J Lipid Res. 2025 Sep 23;66(11):100908. doi: 10.1016/j.jlr.2025.100908 (PMC12594915; doi:10.1016/j.jlr.2025.100908)
Supplement: Supplementary data [file mmc1.pdf]

Supplements

Suppl. Table 1. Primers for Real-time PCR.

Gene symbol	Gene name	Primer sequences (5' - 3')
GAPDH	Glyceraldehyde-3-phosphate dehydrogenase	Forward: AGGTCGGTGTGAACGGATTTG Reverse: TGTAGACCATGTAGTTGAGGTCA
Sptlc1	Serine palmitoyltransferase, long chain base subunit 1	Forward: ACGAGGCTCCAGCATACCAT Reverse: TCAGAACGCTCCTGCAACTTG
Sptlc2	Serine palmitoyltransferase, long chain base subunit 2	Forward: ATATGTGGGCTATGGCGTACT Reverse: TGCATGGTGGCACTTTTCAAT
CerS2	Ceramide synthase 2	Forward: GGCGCTAGAAGTGGGAAAC Reverse: TCGAATGACGAGAAAGAGCA
CerS6	Ceramide synthase 6	Forward: GATTCATAGCCAAACCATGTGCC Reverse: AATGCTCCGAACATCCCAGTC
SGPP1	Sphingosine-1-phosphate phosphatase 1	Forward: GATGCAGAGACCGAGGTTCG Reverse: CGGCAAGTTGCTCA CTTTGAC
ST3GAL5	Ganglioside GM3 synthase	Forward: ATGCCAAGTGAGTT CACCTCT Reverse: ACTCCAAATGCAAC CAACGTG

Suppl. Table 2. In the liver, SMSr is associated with age and obesity in humans and with age and a western diet in mice

Model	Tissue	Lower SMSr Exp	Higher SMSr Exp	P	Source
Human	Liver	Lean(Males)	Obese(Males)	<0.01	Liver Atlas (1，2)
Human	Liver	<=50yrs(Females)	>50yrs(Females)	<0.01	Liver Atlas(1，2)
Human	Liver	<=50yrs(Males)	>50yrs(Males)	<0.01	Xiao et al (3)
Mouse	Liver	Standard diet	Western diet	<0.001	Liver Atlas (1，4)
Mouse	Liver	<3mos(Males)	>9mons(Males)	<0.001	Xiao et al (3)

References:

1. Charlotte Scott and Martin Guilliams. Liver cell atlas, 2023.
2. Martin Guilliams, Johnny Bonnardel, Birthe Haest, Bart Vanderborght, Camille Wagner, Anneleen Remmerie, Anna Bujko, Liesbet Martens, Tinne Thoné, Robin Browaeys, et al. Spatial proteogenomics reveals distinct and evolutionarily conserved hepatic macrophage niches. *Cell*, 185(2):379–396, 2022.
3. Yang Xiao, Kirill Batmanov, Wenxiang Hu, Kun Zhu, Alexander Y Tom, Dongyin Guan, Chunjie Jiang, Lan Cheng, Sam J McCright, Eric C Yang, et al. Hepatocytes demarcated by ephb2 contribute to the progression of nonalcoholic steatohepatitis. *Science translational medicine*, 15(682):eadc9653, 2023.
4. Anneleen Remmerie, Liesbet Martens, Tinne Thoné, Angela Castoldi, Ruth Seurinck, Benjamin Pavie, Joris Roels, Bavo Vanneste, Sofie De Prijck, Mathias Vanhockerhout, et al. Osteopontin expression identifies a subset of recruited macrophages distinct from kupffer cells in the fatty liver. *Immunity*, 53(3):641–657, 2020.

Suppl. Table 3. Pearson correlation coefficients， Spearman rank, and SERE statistics .

gene1	gene2	tissue	pearsonR	Rpv	spearmanR	Spv	SERE
ACER1	ACER2	adipose_su	-0.03328	0.392225	-0.03528	0.364433	-0.0235
ACER1	ACER2	liver	-0.07907	0.236439	-0.22027	0.000856	-0.14796
ACER1	CERS1	adipose_su	0.045827	0.238643	0.071048	0.067512	0.047422
ACER1	CERS1	liver	0.014283	0.830908	0.024138	0.71816	0.018368
ACER1	CERS6	adipose_su	0.030086	0.439286	-0.06755	0.082225	-0.04489
ACER1	CERS6	liver	-0.0872	0.191515	-0.14232	0.032472	-0.10061
ACER1	DEGS2	adipose_su	0.868613	9.37E-204	0.272279	9.83E-13	0.183461
ACER1	DEGS2	liver	0.168633	0.011108	0.235138	0.000363	0.164326
ACER1	SGMS1	adipose_su	0.174958	5.86E-06	0.165246	1.90E-05	0.111404
ACER1	SGMS1	liver	-0.05098	0.445679	0.062829	0.347106	0.040708
ACER1	SPHK1	adipose_su	0.005987	0.877704	0.18561	1.49E-06	0.125375
ACER1	SPHK1	liver	-0.06436	0.335431	-0.24488	0.000201	-0.15933
ACER1	SPTLC3	adipose_su	0.534144	3.53E-50	0.097833	0.011723	0.065754
ACER1	SPTLC3	liver	0.002592	0.969089	-0.02141	0.748911	-0.01565
ACER2	CERS1	adipose_su	-0.17759	4.21E-06	-0.24503	1.60E-10	-0.16485
ACER2	CERS1	liver	0.257316	9.12E-05	0.091563	0.170142	0.06238
ACER2	SGMS1	adipose_su	-0.08939	0.021333	-0.12572	0.001179	-0.0827
ACER2	SGMS1	liver	-0.07484	0.262518	-0.12533	0.059953	-0.08079
ACER3	ACER1	adipose_su	-0.07295	0.06048	-0.0701	0.071246	-0.04684
ACER3	ACER1	liver	-0.03669	0.583216	-0.22634	0.000607	-0.15823
ACER3	ACER2	adipose_su	0.29802	4.58E-15	0.293609	1.20E-14	0.200289
ACER3	ACER2	liver	0.561676	3.44E-20	0.687583	5.63E-33	0.494081
ACER3	B4GALT5	adipose_su	0.292421	1.54E-14	0.285645	6.49E-14	0.194853
ACER3	B4GALT5	liver	0.479178	2.24E-14	0.572171	4.73E-21	0.40767
ACER3	CBR3	adipose_su	-0.23501	9.03E-10	-0.18532	1.55E-06	-0.12666
ACER3	CBR3	liver	0.463599	1.92E-13	0.377499	4.58E-09	0.256441
ACER3	CDR2	adipose_su	-0.2173	1.58E-08	-0.23239	1.40E-09	-0.156
ACER3	CDR2	liver	0.472684	5.57E-14	0.424886	2.54E-11	0.295497
ACER3	CERK	adipose_su	0.240068	3.81E-10	0.213534	2.82E-08	0.144723
ACER3	CERK	liver	0.264448	5.69E-05	0.291664	8.29E-06	0.199095
ACER3	CERS1	adipose_su	-0.16129	3.01E-05	-0.169	1.21E-05	-0.11479
ACER3	CERS1	liver	0.298807	4.83E-06	0.087033	0.192363	0.059862
ACER3	CERS2	adipose_su	0.058197	0.134407	0.087241	0.024679	0.059133
ACER3	CERS2	liver	0.602937	9.24E-24	0.611059	1.59E-24	0.437286
ACER3	CERS3	adipose_su	-0.06711	0.084231	0.038602	0.320975	0.026306
ACER3	CERS3	liver	0.071476	0.28465	0.189254	0.004301	0.124995
ACER3	CERS4	adipose_su	-0.10932	0.004831	-0.06585	0.090251	-0.04423
ACER3	CERS4	liver	0.108615	0.103395	0.088031	0.187296	0.061475
ACER3	CERS5	adipose_su	-0.17625	4.99E-06	-0.19107	7.18E-07	-0.1294
ACER3	CERS5	liver	0.59245	8.35E-23	0.58562	3.36E-22	0.419272
ACER3	CERS6	adipose_su	0.343329	8.89E-20	0.344203	7.08E-20	0.234766
ACER3	CERS6	liver	0.740213	1.74E-40	0.760538	6.77E-44	0.563225
ACER3	DEGS1	adipose_su	0.219149	1.19E-08	0.331664	1.73E-18	0.22757

ACER3	DEGS1	liver	0.689603	3.11E-33	0.736628	6.43E-40	0.544385
ACER3	DEGS2	adipose_su	-0.14286	0.000224	-0.1717	8.76E-06	-0.11562
ACER3	DEGS2	liver	-0.06081	0.362864	-0.25854	8.42E-05	-0.17487
ACER3	EDA2R	adipose_su	-0.14951	0.000112	-0.16736	1.48E-05	-0.11234
ACER3	EDA2R	liver	0.250324	0.000143	0.254931	0.000106	0.169872
ACER3	HKDC1	adipose_su	-0.09346	0.016075	-0.04535	0.243563	-0.03066
ACER3	HKDC1	liver	0.334496	2.61E-07	0.541957	1.18E-18	0.366883
ACER3	KCTD3	adipose_su	0.241282	3.09E-10	0.220506	9.59E-09	0.149244
ACER3	KCTD3	liver	0.229437	0.000508	0.157364	0.017916	0.110403
ACER3	KDSR	adipose_su	0.064629	0.096371	0.084891	0.028841	0.057124
ACER3	KDSR	liver	0.450555	1.07E-12	0.566465	1.40E-20	0.405192
ACER3	MAPK7	adipose_su	-0.22779	2.98E-09	-0.22218	7.36E-09	-0.14902
ACER3	MAPK7	liver	0.658716	1.69E-29	0.728578	1.13E-38	0.532035
ACER3	SEMA4F	adipose_su	0.184067	1.83E-06	0.188841	9.71E-07	0.127708
ACER3	SEMA4F	liver	0.451087	9.97E-13	0.473675	4.85E-14	0.328456
ACER3	SGMS1	adipose_su	0.13631	0.000432	0.125689	0.001182	0.084934
ACER3	SGMS1	liver	0.03343	0.617139	-0.02111	0.752254	-0.01074
ACER3	SGMS2	adipose_su	0.179169	3.45E-06	0.186709	1.29E-06	0.126419
ACER3	SGMS2	liver	0.157465	0.017842	0.526942	1.51E-17	0.380059
ACER3	SGPL1	adipose_su	0.458453	9.14E-36	0.475085	1.25E-38	0.332139
ACER3	SGPL1	liver	0.445435	2.05E-12	0.411843	1.15E-10	0.285034
ACER3	SGPP1	adipose_su	0.454736	3.80E-35	0.430087	3.14E-31	0.295302
ACER3	SGPP1	liver	0.525652	1.86E-17	0.598255	2.49E-23	0.422144
ACER3	SGPP2	adipose_su	-0.02126	0.584683	0.100024	0.009963	0.06693
ACER3	SGPP2	liver	0.212062	0.001342	0.20824	0.001645	0.142655
ACER3	SMPD1	adipose_su	0.077222	0.046857	0.132889	0.000602	0.088661
ACER3	SMPD1	liver	0.625822	5.65E-26	0.713127	2.07E-36	0.510993
ACER3	SMPD2	adipose_su	-0.06019	0.121568	-0.0241	0.5356	-0.01628
ACER3	SMPD2	liver	0.607071	3.79E-24	0.711581	3.43E-36	0.517601
ACER3	SMPD3	adipose_su	-0.14018	0.000294	-0.13827	0.000356	-0.0931
ACER3	SMPD3	liver	0.057422	0.390248	0.088412	0.185386	0.059036
ACER3	SMSr	adipose_su	0.374163	1.85E-23	0.395418	3.07E-26	0.273826
ACER3	SMSr	liver	0.701691	7.94E-35	0.750232	3.99E-42	0.55355
ACER3	SPHK1	adipose_su	-0.2018	1.60E-07	-0.29844	4.18E-15	-0.20158
ACER3	SPHK1	liver	0.305034	2.98E-06	0.421484	3.80E-11	0.294946
ACER3	SPTLC1	adipose_su	0.288652	3.45E-14	0.28533	6.93E-14	0.19272
ACER3	SPTLC1	liver	0.723319	6.92E-38	0.70744	1.30E-35	0.518977
ACER3	SPTLC2	adipose_su	0.251251	5.28E-11	0.244824	1.66E-10	0.166496
ACER3	SPTLC2	liver	0.782598	5.29E-48	0.816354	2.57E-55	0.612075
ACER3	SPTLC3	adipose_su	0.080113	0.039185	0.137671	0.000378	0.092298
ACER3	SPTLC3	liver	0.336554	2.18E-07	0.278325	2.18E-05	0.204444
ACER3	ST3GAL5	adipose_su	-0.0196	0.614406	-0.02143	0.581775	-0.01522
ACER3	ST3GAL5	liver	0.211589	0.001376	0.218926	0.000922	0.150678
ACER3	TOPBP1	adipose_su	0.30957	3.43E-16	0.287659	4.25E-14	0.195696

ACER3	TOPBP1	liver	0.798876	2.35E-51	0.812405	2.19E-54	0.617581
ACER3	TTC39A	adipose_su	0.178533	3.74E-06	0.285348	6.90E-14	0.194279
ACER3	TTC39A	liver	0.348258	7.63E-08	0.660723	9.98E-30	0.471898
ACER3	UGCG	adipose_su	0.11934	0.002083	0.140751	0.000278	0.094667
ACER3	UGCG	liver	0.343413	1.18E-07	0.330485	3.69E-07	0.229499
ACER3	ZDHHC2	adipose_su	0.287759	4.16E-14	0.286701	5.20E-14	0.195773
ACER3	ZDHHC2	liver	0.711268	3.80E-36	0.721912	1.12E-37	0.524995
B4GALT5	ACER1	adipose_su	-0.0434	0.264509	0.046608	0.230732	0.032276
B4GALT5	ACER1	liver	-0.19532	0.003194	-0.39934	4.62E-10	-0.27253
B4GALT5	ACER2	adipose_su	0.107257	0.005701	0.207856	6.63E-08	0.138672
B4GALT5	ACER2	liver	0.467748	1.10E-13	0.681866	2.96E-32	0.495025
B4GALT5	CBR3	adipose_su	-0.17251	7.93E-06	-0.18321	2.05E-06	-0.12177
B4GALT5	CBR3	liver	0.169715	0.010594	0.132444	0.046724	0.091091
B4GALT5	CERS1	adipose_su	0.025785	0.507472	0.094931	0.014475	0.063581
B4GALT5	CERS1	liver	0.253489	0.000117	0.038119	0.568625	0.024031
B4GALT5	CERS6	adipose_su	-0.06263	0.10713	-0.07777	0.04532	-0.0501
B4GALT5	CERS6	liver	0.416894	6.47E-11	0.564673	1.96E-20	0.406922
B4GALT5	DEGS2	adipose_su	-0.08702	0.02504	-0.10538	0.00661	-0.07053
B4GALT5	DEGS2	liver	0.034411	0.606838	-0.21047	0.001462	-0.13522
B4GALT5	MAPK7	adipose_su	0.126963	0.001052	0.20533	9.61E-08	0.138011
B4GALT5	MAPK7	liver	0.523197	2.79E-17	0.695651	5.07E-34	0.513078
B4GALT5	SGMS1	adipose_su	0.078717	0.042747	0.183216	2.04E-06	0.125207
B4GALT5	SGMS1	liver	-0.07347	0.271366	-0.1183	0.075929	-0.07886
B4GALT5	SGMS2	adipose_su	0.322309	1.72E-17	0.280285	1.96E-13	0.190168
B4GALT5	SGMS2	liver	0.586193	2.99E-22	0.746954	1.40E-41	0.561613
B4GALT5	SGPL1	adipose_su	-0.0452	0.245093	-0.00235	0.951866	-0.00175
B4GALT5	SGPL1	liver	0.085624	0.199694	0.117456	0.078058	0.08531
B4GALT5	SGPP2	adipose_su	0.056865	0.143569	-0.01711	0.660092	-0.01317
B4GALT5	SGPP2	liver	0.31564	1.28E-06	0.273585	3.05E-05	0.183953
B4GALT5	SMPD1	adipose_su	-0.00498	0.898111	0.035823	0.357075	0.022406
B4GALT5	SMPD1	liver	0.529231	1.03E-17	0.643418	8.33E-28	0.461357
B4GALT5	SPHK1	adipose_su	0.444315	1.89E-33	0.329329	3.10E-18	0.226659
B4GALT5	SPHK1	liver	0.403878	2.81E-10	0.534747	4.08E-18	0.37998
B4GALT5	SPTLC3	adipose_su	-0.04269	0.272321	0.04909	0.20681	0.036158
B4GALT5	SPTLC3	liver	-0.00609	0.927481	0.005755	0.93144	0.014159
B4GALT5	TOPBP1	adipose_su	0.131363	0.000697	0.196481	3.41E-07	0.134899
B4GALT5	TOPBP1	liver	0.435397	7.17E-12	0.529209	1.03E-17	0.37703
CBR3	ACER1	adipose_su	-0.01316	0.735218	0.159757	3.59E-05	0.107098
CBR3	ACER1	liver	0.118513	0.075399	-0.06031	0.366832	-0.04264
CBR3	ACER2	adipose_su	-0.30802	4.88E-16	-0.29355	1.21E-14	-0.19977
CBR3	ACER2	liver	0.19169	0.00382	0.187306	0.004724	0.124877
CBR3	CERS1	adipose_su	0.143578	0.000208	0.149146	0.000116	0.099657
CBR3	CERS1	liver	0.48086	1.77E-14	0.201171	0.002377	0.13412
CBR3	CERS6	adipose_su	-0.27793	3.17E-13	-0.30253	1.69E-15	-0.20316

CBR3	CERS6	liver	0.222894	0.000739	0.200804	0.002422	0.134395
CBR3	DEGS2	adipose_su	0.110437	0.004414	0.359586	1.14E-21	0.244955
CBR3	DEGS2	liver	0.04293	0.520808	0.085178	0.202053	0.05471
CBR3	MAPK7	adipose_su	0.161394	2.98E-05	0.206235	8.42E-08	0.142627
CBR3	MAPK7	liver	0.596481	3.62E-23	0.312178	1.69E-06	0.210816
CBR3	SGMS1	adipose_su	0.408001	5.54E-28	0.438998	1.31E-32	0.302589
CBR3	SGMS1	liver	0.144448	0.02994	0.137594	0.038747	0.090698
CBR3	SGMS2	adipose_su	0.178282	3.86E-06	0.240971	3.26E-10	0.166359
CBR3	SGMS2	liver	0.062731	0.347857	0.148341	0.025745	0.102812
CBR3	SGPL1	adipose_su	-0.09662	0.012809	-0.03361	0.387579	-0.02267
CBR3	SGPL1	liver	0.328097	4.53E-07	0.312575	1.64E-06	0.208142
CBR3	SGPP2	adipose_su	0.042671	0.272577	0.161552	2.92E-05	0.107791
CBR3	SGPP2	liver	0.339179	1.73E-07	0.252198	0.000127	0.175654
CBR3	SMPD1	adipose_su	0.372788	2.75E-23	0.393607	5.39E-26	0.269552
CBR3	SMPD1	liver	0.133519	0.044954	0.20364	0.002093	0.132035
CBR3	SPHK1	adipose_su	0.130025	0.000791	0.254612	2.86E-11	0.177478
CBR3	SPHK1	liver	0.446785	1.73E-12	0.296612	5.71E-06	0.200747
CBR3	SPTLC3	adipose_su	0.226639	3.60E-09	0.307259	5.81E-16	0.212419
CBR3	SPTLC3	liver	0.097547	0.143795	0.177895	0.007342	0.119843
CBR3	TOPBP1	adipose_su	0.196241	3.52E-07	0.232445	1.39E-09	0.157141
CBR3	TOPBP1	liver	0.502623	7.17E-16	0.37272	7.39E-09	0.252311
CDR2	ACER1	adipose_su	-0.01306	0.737111	0.153304	7.39E-05	0.102578
CDR2	ACER1	liver	-0.13388	0.044374	-0.19232	0.003704	-0.12794
CDR2	ACER2	adipose_su	0.074632	0.054766	0.014271	0.713773	0.010663
CDR2	ACER2	liver	0.471967	6.15E-14	0.452926	7.86E-13	0.31119
CDR2	B4GALT5	adipose_su	0.294311	1.03E-14	0.344225	7.04E-20	0.234542
CDR2	B4GALT5	liver	0.508135	3.07E-16	0.497474	1.56E-15	0.352134
CDR2	CBR3	adipose_su	0.150562	9.95E-05	0.211651	3.76E-08	0.141215
CDR2	CBR3	liver	0.400669	4.00E-10	0.238324	0.0003	0.166765
CDR2	CERS1	adipose_su	0.219145	1.19E-08	0.298267	4.34E-15	0.202517
CDR2	CERS1	liver	0.426542	2.09E-11	0.141131	0.033962	0.095143
CDR2	CERS2	adipose_su	0.240508	3.53E-10	0.176453	4.86E-06	0.118226
CDR2	CERS2	liver	0.222672	0.000748	0.264406	5.70E-05	0.182183
CDR2	CERS3	adipose_su	-0.02613	0.501777	0.05119	0.188017	0.034057
CDR2	CERS3	liver	0.021296	0.750173	0.23741	0.000317	0.159764
CDR2	CERS6	adipose_su	-0.23976	4.02E-10	-0.36388	3.46E-22	-0.24043
CDR2	CERS6	liver	0.455192	5.85E-13	0.41975	4.65E-11	0.294199
CDR2	DEGS1	adipose_su	0.187026	1.24E-06	0.261775	7.52E-12	0.176744
CDR2	DEGS1	liver	0.533044	5.44E-18	0.470786	7.23E-14	0.333176
CDR2	DEGS2	adipose_su	0.018889	0.627324	0.200959	1.81E-07	0.130903
CDR2	DEGS2	liver	0.112359	0.09197	0.023412	0.726294	0.021711
CDR2	HKDC1	adipose_su	0.096512	0.012912	0.09164	0.018269	0.061717
CDR2	HKDC1	liver	0.381771	2.96E-09	0.396458	6.32E-10	0.273432
CDR2	MAPK7	adipose_su	0.298809	3.85E-15	0.340439	1.88E-19	0.231307

CDR2	MAPK7	liver	0.602173	1.09E-23	0.47333	5.09E-14	0.331839
CDR2	SGMS1	adipose_su	0.351054	1.16E-20	0.414717	6.05E-29	0.285715
CDR2	SGMS1	liver	0.111444	0.094664	0.121558	0.068144	0.08177
CDR2	SGMS2	adipose_su	0.11343	0.003449	0.144697	0.000185	0.097766
CDR2	SGMS2	liver	0.445778	1.97E-12	0.455239	5.82E-13	0.322793
CDR2	SGPL1	adipose_su	-0.29263	1.47E-14	-0.3134	1.42E-16	-0.21275
CDR2	SGPL1	liver	0.04777	0.474879	0.061917	0.354161	0.048968
CDR2	SGPP2	adipose_su	-0.05536	0.154491	-0.05902	0.129007	-0.03901
CDR2	SGPP2	liver	0.275869	2.60E-05	0.225668	0.000631	0.15296
CDR2	SMPD1	adipose_su	-0.05529	0.154988	-0.06052	0.119523	-0.04009
CDR2	SMPD1	liver	0.253516	0.000117	0.295672	6.13E-06	0.201613
CDR2	SMSr	adipose_su	0.200668	1.89E-07	0.147151	0.000143	0.098654
CDR2	SMSr	liver	0.584175	4.49E-22	0.508217	3.03E-16	0.367512
CDR2	SPHK1	adipose_su	0.468642	1.68E-37	0.566181	1.83E-57	0.398322
CDR2	SPHK1	liver	0.51999	4.69E-17	0.431586	1.14E-11	0.304543
CDR2	SPTLC3	adipose_su	0.061696	0.112485	0.167634	1.43E-05	0.112958
CDR2	SPTLC3	liver	-0.13852	0.037448	-0.11236	0.091957	-0.07032
CDR2	TOPBP1	adipose_su	0.140512	0.000284	0.190846	7.41E-07	0.128747
CDR2	TOPBP1	liver	0.516317	8.46E-17	0.44019	3.97E-12	0.311583
CDR2	UGCG	adipose_su	0.348611	2.22E-20	0.399491	8.52E-27	0.27651
CDR2	UGCG	liver	0.604576	6.50E-24	0.602684	9.75E-24	0.435516
CERK	ACER1	adipose_su	-0.03362	0.387409	0.133793	0.000552	0.088948
CERK	ACER1	liver	-0.04202	0.529659	-0.01052	0.874983	-0.00952
CERK	ACER2	adipose_su	0.304162	1.17E-15	0.247595	1.02E-10	0.16753
CERK	ACER2	liver	0.145679	0.028554	0.143269	0.03132	0.095143
CERK	B4GALT5	adipose_su	0.11441	0.003177	0.116522	0.002657	0.079908
CERK	B4GALT5	liver	0.183509	0.005658	0.111811	0.093575	0.073864
CERK	CBR3	adipose_su	-0.04045	0.298381	0.01619	0.677333	0.011898
CERK	CBR3	liver	0.3237	6.56E-07	0.292408	7.84E-06	0.202281
CERK	CDR2	adipose_su	0.111877	0.003923	0.08181	0.035199	0.05506
CERK	CDR2	liver	0.384557	2.22E-09	0.353169	4.85E-08	0.246372
CERK	CERS1	adipose_su	-0.10078	0.009416	-0.03657	0.34719	-0.02456
CERK	CERS1	liver	0.096276	0.149115	0.018778	0.778899	0.011917
CERK	CERS2	adipose_su	0.235078	8.92E-10	0.269687	1.64E-12	0.184413
CERK	CERS2	liver	0.100546	0.131815	0.159982	0.016073	0.109145
CERK	CERS3	adipose_su	-0.02856	0.46283	0.090459	0.019827	0.062104
CERK	CERS3	liver	-0.06813	0.307825	0.023125	0.729523	0.016991
CERK	CERS5	adipose_su	0.178582	3.71E-06	0.153691	7.08E-05	0.10276
CERK	CERS5	liver	0.493403	2.87E-15	0.367455	1.24E-08	0.256323
CERK	CERS6	adipose_su	0.231994	1.49E-09	0.261913	7.33E-12	0.178293
CERK	CERS6	liver	0.181386	0.006249	0.239355	0.000282	0.163186
CERK	DEGS1	adipose_su	0.109147	0.0049	0.161235	3.03E-05	0.108707
CERK	DEGS1	liver	0.256782	9.44E-05	0.153861	0.020668	0.101554
CERK	DEGS2	adipose_su	0.010178	0.793644	0.096114	0.013291	0.06323

CERK	DEGS2	liver	0.080851	0.226009	0.108786	0.10285	0.072409
CERK	EDA2R	adipose_su	-0.01988	0.609451	-0.02642	0.497062	-0.01796
CERK	EDA2R	liver	0.083226	0.212621	0.100023	0.133849	0.067178
CERK	HKDC1	adipose_su	-0.0398	0.306234	0.080464	0.03833	0.053683
CERK	HKDC1	liver	0.126641	0.05731	0.257075	9.26E-05	0.176952
CERK	KCTD3	adipose_su	0.385246	6.96E-25	0.421048	7.16E-30	0.291033
CERK	KCTD3	liver	0.037514	0.574781	0.126816	0.056964	0.08826
CERK	KDSR	adipose_su	0.28624	5.73E-14	0.322853	1.50E-17	0.220362
CERK	KDSR	liver	0.423079	3.15E-11	0.2831	1.55E-05	0.197089
CERK	MAPK7	adipose_su	0.16552	1.84E-05	0.204643	1.06E-07	0.135947
CERK	MAPK7	liver	0.346153	9.24E-08	0.254261	0.000111	0.173687
CERK	SEMA4F	adipose_su	-0.03949	0.309924	-0.04603	0.236607	-0.03082
CERK	SEMA4F	liver	0.142807	0.031876	0.236434	0.000336	0.16826
CERK	SGMS1	adipose_su	0.067869	0.080766	0.095256	0.01414	0.063111
CERK	SGMS1	liver	0.103344	0.121349	0.194001	0.00341	0.132232
CERK	SGMS2	adipose_su	0.042119	0.27883	0.091088	0.018983	0.060596
CERK	SGMS2	liver	0.352822	5.01E-08	0.325365	5.71E-07	0.222458
CERK	SGPL1	adipose_su	0.249714	6.97E-11	0.306121	7.52E-16	0.210168
CERK	SGPL1	liver	0.25363	0.000116	0.321916	7.62E-07	0.229302
CERK	SGPP1	adipose_su	0.246966	1.14E-10	0.261253	8.30E-12	0.176206
CERK	SGPP1	liver	0.085378	0.200994	0.122752	0.065459	0.084248
CERK	SGPP2	adipose_su	0.088171	0.023181	0.225262	4.50E-09	0.151923
CERK	SGPP2	liver	0.469306	8.86E-14	0.280117	1.92E-05	0.188161
CERK	SMPD1	adipose_su	0.103593	0.007595	0.132733	0.000612	0.090443
CERK	SMPD1	liver	0.042638	0.523648	0.068741	0.303534	0.04645
CERK	SMPD2	adipose_su	-0.04661	0.230737	-0.0419	0.281371	-0.02763
CERK	SMPD2	liver	0.511878	1.71E-16	0.256765	9.45E-05	0.17821
CERK	SMPD3	adipose_su	0.001231	0.974758	0.023093	0.552806	0.018496
CERK	SMPD3	liver	-0.14688	0.027254	-0.24385	0.000214	-0.16181
CERK	SMSr	adipose_su	0.416335	3.52E-29	0.430576	2.65E-31	0.295384
CERK	SMSr	liver	0.364979	1.58E-08	0.321412	7.95E-07	0.224149
CERK	SPHK1	adipose_su	0.076954	0.04763	-0.00715	0.854253	-0.0041
CERK	SPHK1	liver	0.610373	1.84E-24	0.282723	1.60E-05	0.192134
CERK	SPTLC2	adipose_su	0.279007	2.55E-13	0.259564	1.14E-11	0.175331
CERK	SPTLC2	liver	0.315132	1.33E-06	0.185384	0.005178	0.127788
CERK	SPTLC3	adipose_su	-0.13701	0.000403	-0.12954	0.000828	-0.08842
CERK	SPTLC3	liver	-0.20693	0.001763	-0.18149	0.006219	-0.12118
CERK	ST3GAL5	adipose_su	0.118607	0.002221	0.128623	0.000902	0.085645
CERK	ST3GAL5	liver	-0.13086	0.049434	-0.15638	0.018655	-0.10627
CERK	TOPBP1	adipose_su	0.4173	2.55E-29	0.417072	2.75E-29	0.290882
CERK	TOPBP1	liver	0.357067	3.37E-08	0.347631	8.08E-08	0.244995
CERK	UGCG	adipose_su	-0.03067	0.430523	-0.01497	0.700456	-0.00823
CERK	UGCG	liver	0.176511	0.00782	0.238332	0.0003	0.165428
CERK	ZDHHC2	adipose_su	0.149753	0.000109	0.168557	1.28E-05	0.115478

CERK	ZDHHC2	liver	0.190026	0.004143	0.260933	7.19E-05	0.183324
CERS2	ACER1	adipose_su	-0.02816	0.469128	0.101879	0.008661	0.068233
CERS2	ACER1	liver	-0.16578	0.012574	-0.25928	8.02E-05	-0.18045
CERS2	ACER2	adipose_su	0.136077	0.000442	0.13872	0.00034	0.091418
CERS2	ACER2	liver	0.334788	2.54E-07	0.540282	1.58E-18	0.377856
CERS2	B4GALT5	adipose_su	0.225062	4.64E-09	0.251721	4.85E-11	0.169563
CERS2	B4GALT5	liver	0.490481	4.41E-15	0.552202	1.94E-19	0.396067
CERS2	CBR3	adipose_su	0.087209	0.024731	0.100861	0.009355	0.068188
CERS2	CBR3	liver	0.1531	0.021311	0.196259	0.003047	0.131878
CERS2	CERS1	adipose_su	0.145618	0.000168	0.132422	0.00063	0.087249
CERS2	CERS1	liver	-0.00157	0.981322	-0.05019	0.45277	-0.02828
CERS2	CERS3	adipose_su	-0.02072	0.594419	0.238003	5.43E-10	0.161128
CERS2	CERS3	liver	0.02686	0.687963	0.118572	0.075251	0.079174
CERS2	CERS6	adipose_su	0.397052	1.84E-26	0.412256	1.37E-28	0.277718
CERS2	CERS6	liver	0.698672	2.02E-34	0.710118	5.51E-36	0.517483
CERS2	DEGS1	adipose_su	0.189415	8.99E-07	0.294778	9.28E-15	0.1994
CERS2	DEGS1	liver	0.530666	8.12E-18	0.577059	1.84E-21	0.413215
CERS2	DEGS2	adipose_su	0.02649	0.49592	0.110202	0.004499	0.07295
CERS2	DEGS2	liver	-0.07599	0.25527	-0.23536	0.000359	-0.15768
CERS2	HKDC1	adipose_su	0.099298	0.010518	0.149257	0.000115	0.101416
CERS2	HKDC1	liver	0.248572	0.00016	0.344606	1.06E-07	0.22942
CERS2	MAPK7	adipose_su	0.424851	1.94E-30	0.434948	5.62E-32	0.298082
CERS2	MAPK7	liver	0.348242	7.64E-08	0.511599	1.79E-16	0.356893
CERS2	SGMS1	adipose_su	0.31145	2.22E-16	0.321291	2.19E-17	0.215823
CERS2	SGMS1	liver	0.245947	0.000188	0.245635	0.000192	0.163854
CERS2	SGMS2	adipose_su	0.223396	6.06E-09	0.228404	2.70E-09	0.153842
CERS2	SGMS2	liver	0.129186	0.052446	0.501708	8.24E-16	0.362399
CERS2	SGPL1	adipose_su	0.314603	1.07E-16	0.364937	2.58E-22	0.249097
CERS2	SGPL1	liver	0.577486	1.69E-21	0.562279	3.07E-20	0.399607
CERS2	SGPP2	adipose_su	-0.04514	0.245757	-0.08312	0.032362	-0.05571
CERS2	SGPP2	liver	0.028804	0.666678	0.114822	0.085018	0.075044
CERS2	SMPD1	adipose_su	0.300771	2.50E-15	0.306133	7.50E-16	0.209653
CERS2	SMPD1	liver	0.705651	2.29E-35	0.735261	1.05E-39	0.551229
CERS2	SMSr	adipose_su	0.493636	5.12E-42	0.493133	6.36E-42	0.33918
CERS2	SMSr	liver	0.461243	2.63E-13	0.544936	7.04E-19	0.386588
CERS2	SPHK1	adipose_su	0.096998	0.012463	0.053688	0.167347	0.036951
CERS2	SPHK1	liver	0.019787	0.767352	0.131572	0.048201	0.092822
CERS2	SPTLC3	adipose_su	-0.04825	0.214656	-0.02929	0.451479	-0.01931
CERS2	SPTLC3	liver	0.574308	3.13E-21	0.461887	2.42E-13	0.328378
CERS2	TOPBP1	adipose_su	0.415323	4.94E-29	0.431775	1.73E-31	0.299335
CERS2	TOPBP1	liver	0.613018	1.03E-24	0.666706	2.02E-30	0.483736
CERS2	UGCG	adipose_su	0.009583	0.805453	-0.0127	0.744076	-0.00929
CERS2	UGCG	liver	-0.03287	0.623036	0.069903	0.295413	0.050108
CERS3	ACER1	adipose_su	0.98988	0	0.184387	1.75E-06	0.125448

CERS3	ACER1	liver	0.228859	0.000525	0.018571	0.781274	0.013845
CERS3	ACER2	adipose_su	-0.03057	0.431959	-0.02163	0.578278	-0.01524
CERS3	ACER2	liver	0.05234	0.433618	0.228549	0.000535	0.154848
CERS3	B4GALT5	adipose_su	-0.04667	0.230091	-0.02623	0.500156	-0.01915
CERS3	B4GALT5	liver	0.097819	0.142673	0.177905	0.007338	0.117837
CERS3	CBR3	adipose_su	-0.01864	0.631805	0.124628	0.001302	0.083439
CERS3	CBR3	liver	0.12768	0.055282	0.21246	0.001313	0.141475
CERS3	CERS1	adipose_su	0.029664	0.445739	0.127404	0.00101	0.082847
CERS3	CERS1	liver	0.143127	0.03149	0.185302	0.005198	0.126254
CERS3	CERS6	adipose_su	0.043271	0.265885	0.080646	0.037893	0.054312
CERS3	CERS6	liver	0.039911	0.550576	0.188746	0.004408	0.123068
CERS3	DEGS2	adipose_su	0.89133	2.80E-229	0.238716	4.81E-10	0.163762
CERS3	DEGS2	liver	0.203638	0.002093	0.006079	0.927585	0.004051
CERS3	HKDC1	adipose_su	0.098973	0.010776	0.16459	2.05E-05	0.109823
CERS3	HKDC1	liver	0.066387	0.32043	0.274044	2.95E-05	0.183402
CERS3	MAPK7	adipose_su	0.055462	0.15373	0.079444	0.040859	0.052722
CERS3	MAPK7	liver	0.076051	0.254867	0.218036	0.000969	0.147257
CERS3	SGMS1	adipose_su	0.176751	4.68E-06	0.236209	7.37E-10	0.15812
CERS3	SGMS1	liver	-0.00664	0.920874	0.127798	0.055055	0.084366
CERS3	SGMS2	adipose_su	0.024429	0.53005	0.057159	0.141511	0.038532
CERS3	SGMS2	liver	-0.00857	0.898028	0.1655	0.012723	0.109341
CERS3	SGPL1	adipose_su	0.228775	2.54E-09	0.198682	2.50E-07	0.136685
CERS3	SGPL1	liver	0.003906	0.953432	0.012363	0.853358	0.006883
CERS3	SGPP2	adipose_su	0.730153	1.99E-111	0.129366	0.000841	0.08816
CERS3	SGPP2	liver	0.201149	0.002379	0.162318	0.014571	0.106116
CERS3	SMPD1	adipose_su	0.020807	0.592782	0.061294	0.114852	0.041161
CERS3	SMPD1	liver	-0.00553	0.934108	0.115256	0.083839	0.076578
CERS3	SMSr	adipose_su	-0.02546	0.512872	0.120708	0.001848	0.0805
CERS3	SMSr	liver	0.079931	0.231353	0.28637	1.22E-05	0.188673
CERS3	SPHK1	adipose_su	-0.00623	0.872697	-0.02053	0.597747	-0.01378
CERS3	SPHK1	liver	0.021703	0.745567	0.205821	0.001868	0.137502
CERS3	SPTLC3	adipose_su	0.530887	1.76E-49	0.182816	2.15E-06	0.122222
CERS3	SPTLC3	liver	0.059807	0.370836	0.118813	0.074657	0.075556
CERS3	TOPBP1	adipose_su	0.064761	0.095688	0.218317	1.35E-08	0.146861
CERS3	TOPBP1	liver	0.082394	0.217247	0.25538	0.000103	0.167984
CERS4	ACER1	adipose_su	0.059145	0.128169	0.205212	9.78E-08	0.136909
CERS4	ACER1	liver	-0.04667	0.48509	0.112784	0.090737	0.073746
CERS4	ACER2	adipose_su	-0.15304	7.61E-05	-0.16389	2.23E-05	-0.10902
CERS4	ACER2	liver	-0.08872	0.183844	-0.04912	0.46244	-0.03209
CERS4	B4GALT5	adipose_su	-0.01119	0.773608	0.009782	0.801504	0.006179
CERS4	B4GALT5	liver	-0.18439	0.005428	-0.1375	0.038876	-0.08838
CERS4	CBR3	adipose_su	0.405004	1.46E-27	0.364806	2.67E-22	0.250072
CERS4	CBR3	liver	0.044194	0.508599	0.067766	0.310454	0.044916
CERS4	CDR2	adipose_su	0.084439	0.029706	0.103609	0.007586	0.068981

CERS4	CDR2	liver	-0.0757	0.257068	-0.05398	0.419307	-0.03544
CERS4	CERK	adipose_su	-0.01993	0.608479	0.012918	0.739884	0.007824
CERS4	CERK	liver	-0.12278	0.0654	-0.06906	0.301274	-0.04688
CERS4	CERS1	adipose_su	0.215344	2.14E-08	0.198416	2.60E-07	0.133805
CERS4	CERS1	liver	-0.10786	0.105836	-0.03262	0.625686	-0.02234
CERS4	CERS2	adipose_su	0.439874	9.56E-33	0.438771	1.43E-32	0.301495
CERS4	CERS2	liver	0.403973	2.78E-10	0.389167	1.37E-09	0.268869
CERS4	CERS3	adipose_su	0.061652	0.112743	0.188839	9.72E-07	0.127485
CERS4	CERS3	liver	-0.01142	0.864403	0.027216	0.684046	0.015064
CERS4	CERS5	adipose_su	0.447337	6.16E-34	0.442571	3.58E-33	0.305036
CERS4	CERS5	liver	-0.15867	0.016972	-0.11543	0.083367	-0.07316
CERS4	CERS6	adipose_su	-0.07894	0.042158	-0.0697	0.072908	-0.04739
CERS4	CERS6	liver	0.144213	0.030211	0.111458	0.094623	0.078741
CERS4	DEGS1	adipose_su	0.06469	0.096054	0.064486	0.09711	0.043399
CERS4	DEGS1	liver	-0.11603	0.081754	-0.09165	0.169717	-0.05487
CERS4	DEGS2	adipose_su	0.152451	8.11E-05	0.268105	2.23E-12	0.180312
CERS4	DEGS2	liver	-0.07553	0.258174	0.022889	0.732177	0.013137
CERS4	EDA2R	adipose_su	0.387209	3.84E-25	0.400164	6.88E-27	0.27389
CERS4	EDA2R	liver	0.056342	0.399239	0.076026	0.255022	0.054631
CERS4	HKDC1	adipose_su	0.238692	4.83E-10	0.287892	4.05E-14	0.195163
CERS4	HKDC1	liver	-0.02059	0.758246	0.05605	0.401688	0.038781
CERS4	KCTD3	adipose_su	0.078886	0.0423	0.133971	0.000543	0.08955
CERS4	KCTD3	liver	0.498488	1.34E-15	0.517303	7.23E-17	0.361809
CERS4	KDSR	adipose_su	0.469129	1.38E-37	0.468863	1.54E-37	0.324484
CERS4	KDSR	liver	-0.21035	0.00147	-0.1404	0.034907	-0.0885
CERS4	MAPK7	adipose_su	0.395705	2.81E-26	0.421423	6.30E-30	0.285382
CERS4	MAPK7	liver	-0.10123	0.12921	-0.0831	0.213322	-0.05164
CERS4	SEMA4F	adipose_su	0.281257	1.61E-13	0.283069	1.11E-13	0.193212
CERS4	SEMA4F	liver	0.158802	0.016882	0.233117	0.000409	0.160197
CERS4	SGMS1	adipose_su	0.415332	4.92E-29	0.396662	2.08E-26	0.270956
CERS4	SGMS1	liver	0.512753	1.49E-16	0.53476	4.07E-18	0.387532
CERS4	SGMS2	adipose_su	0.297085	5.62E-15	0.360895	7.95E-22	0.245
CERS4	SGMS2	liver	-0.19835	0.002744	-0.07763	0.245111	-0.04409
CERS4	SGPL1	adipose_su	0.090074	0.020361	0.132538	0.000623	0.087837
CERS4	SGPL1	liver	0.409518	1.50E-10	0.411317	1.22E-10	0.290226
CERS4	SGPP1	adipose_su	0.231032	1.75E-09	0.25612	2.17E-11	0.172811
CERS4	SGPP1	liver	-0.09366	0.160536	-0.10159	0.127841	-0.06541
CERS4	SGPP2	adipose_su	0.061261	0.115052	0.028233	0.467998	0.019125
CERS4	SGPP2	liver	-0.18397	0.005537	-0.20854	0.001619	-0.14187
CERS4	SMPD1	adipose_su	0.495359	2.42E-42	0.4991	4.69E-43	0.347017
CERS4	SMPD1	liver	0.259111	8.11E-05	0.205841	0.001866	0.134592
CERS4	SMPD2	adipose_su	0.41797	2.03E-29	0.403997	2.02E-27	0.277426
CERS4	SMPD2	liver	-0.08773	0.188832	-0.05716	0.392422	-0.03147
CERS4	SMPD3	adipose_su	0.20925	5.39E-08	0.197607	2.91E-07	0.13309

CERS4	SMPD3	liver	0.034641	0.604436	0.148764	0.025321	0.100806
CERS4	SMSr	adipose_su	0.045339	0.243683	0.061815	0.111793	0.041016
CERS4	SMSr	liver	-0.1036	0.120418	-0.06338	0.342872	-0.03823
CERS4	SPHK1	adipose_su	0.107882	0.005424	0.173332	7.17E-06	0.116435
CERS4	SPHK1	liver	-0.13331	0.045291	-0.22517	0.000649	-0.14997
CERS4	SPTLC2	adipose_su	-0.14084	0.000275	-0.12398	0.001381	-0.08248
CERS4	SPTLC2	liver	0.023308	0.727461	0.043546	0.514838	0.03469
CERS4	SPTLC3	adipose_su	0.23883	4.71E-10	0.259663	1.12E-11	0.17694
CERS4	SPTLC3	liver	0.540017	1.66E-18	0.531708	6.82E-18	0.374749
CERS4	ST3GAL5	adipose_su	0.501503	1.62E-43	0.490774	1.76E-41	0.341991
CERS4	ST3GAL5	liver	0.22331	0.000722	0.271136	3.61E-05	0.185683
CERS4	TOPBP1	adipose_su	0.246734	1.19E-10	0.274315	6.56E-13	0.185156
CERS4	TOPBP1	liver	0.122986	0.064943	0.136517	0.040312	0.095969
CERS4	UGCG	adipose_su	-0.06402	0.09958	-0.05079	0.191493	-0.03452
CERS4	UGCG	liver	-0.10685	0.109174	-0.04559	0.495295	-0.02686
CERS4	ZDHHC2	adipose_su	0.164844	1.99E-05	0.145443	0.000171	0.096294
CERS4	ZDHHC2	liver	0.051505	0.440997	0.034531	0.605584	0.022734
CERS5	ACER1	adipose_su	0.119386	0.002075	0.223644	5.83E-09	0.151158
CERS5	ACER1	liver	-0.04467	0.504068	-0.19017	0.004114	-0.12995
CERS5	ACER2	adipose_su	-0.1622	2.71E-05	-0.22491	4.76E-09	-0.14949
CERS5	ACER2	liver	0.479294	2.21E-14	0.537685	2.47E-18	0.385487
CERS5	B4GALT5	adipose_su	0.245458	1.49E-10	0.261326	8.19E-12	0.177122
CERS5	B4GALT5	liver	0.478957	2.32E-14	0.526707	1.57E-17	0.380098
CERS5	CBR3	adipose_su	0.309659	3.36E-16	0.312205	1.87E-16	0.216215
CERS5	CBR3	liver	0.554914	1.19E-19	0.38171	2.98E-09	0.26411
CERS5	CDR2	adipose_su	0.507418	1.13E-44	0.521616	1.55E-47	0.364711
CERS5	CDR2	liver	0.62845	3.06E-26	0.469022	9.21E-14	0.336283
CERS5	CERS1	adipose_su	0.295836	7.38E-15	0.378056	5.93E-24	0.25528
CERS5	CERS1	liver	0.471877	6.22E-14	0.175164	0.008312	0.11941
CERS5	CERS2	adipose_su	0.471695	4.93E-38	0.449777	2.48E-34	0.311465
CERS5	CERS2	liver	0.188547	0.004451	0.271366	3.55E-05	0.188358
CERS5	CERS3	adipose_su	0.110111	0.004532	0.156924	4.95E-05	0.105221
CERS5	CERS3	liver	0.09212	0.167547	0.216538	0.001052	0.141908
CERS5	CERS6	adipose_su	-0.19236	6.03E-07	-0.23212	1.46E-09	-0.15407
CERS5	CERS6	liver	0.390202	1.23E-09	0.390763	1.16E-09	0.277955
CERS5	DEGS1	adipose_su	0.193191	5.38E-07	0.184188	1.80E-06	0.124227
CERS5	DEGS1	liver	0.56054	4.24E-20	0.576289	2.13E-21	0.409204
CERS5	DEGS2	adipose_su	0.211862	3.64E-08	0.351877	9.30E-21	0.239395
CERS5	DEGS2	liver	0.222326	0.000763	0.083281	0.212321	0.057856
CERS5	HKDC1	adipose_su	0.239599	4.13E-10	0.302988	1.52E-15	0.203474
CERS5	HKDC1	liver	0.32053	8.55E-07	0.500333	1.02E-15	0.348594
CERS5	MAPK7	adipose_su	0.608014	2.89E-68	0.615539	2.21E-70	0.438572
CERS5	MAPK7	liver	0.791104	1.02E-49	0.674107	2.65E-31	0.494946
CERS5	SGMS1	adipose_su	0.466368	4.14E-37	0.464145	9.96E-37	0.324607

CERS5	SGMS1	liver	0.034382	0.607143	0.015729	0.814084	0.010619
CERS5	SGMS2	adipose_su	0.379414	3.97E-24	0.427202	8.61E-31	0.29281
CERS5	SGMS2	liver	0.311888	1.73E-06	0.433889	8.62E-12	0.302852
CERS5	SGPL1	adipose_su	-0.03407	0.381111	-0.05174	0.183348	-0.03298
CERS5	SGPL1	liver	0.231758	0.000444	0.212499	0.001311	0.148515
CERS5	SGPP2	adipose_su	0.136019	0.000445	0.090722	0.019471	0.062328
CERS5	SGPP2	liver	0.403879	2.81E-10	0.264569	5.64E-05	0.179508
CERS5	SMPD1	adipose_su	0.368999	8.16E-23	0.339664	2.29E-19	0.230892
CERS5	SMPD1	liver	0.270311	3.82E-05	0.375854	5.40E-09	0.262065
CERS5	SMSr	adipose_su	0.153156	7.51E-05	0.138603	0.000344	0.093273
CERS5	SMSr	liver	0.723812	5.85E-38	0.682746	2.30E-32	0.512606
CERS5	SPHK1	adipose_su	0.496935	1.22E-42	0.585969	2.24E-62	0.41341
CERS5	SPHK1	liver	0.625601	5.94E-26	0.631202	1.60E-26	0.459076
CERS5	SPTLC3	adipose_su	0.201244	1.74E-07	0.223591	5.88E-09	0.150757
CERS5	SPTLC3	liver	-0.18067	0.006459	-0.09793	0.142203	-0.05794
CERS5	TOPBP1	adipose_su	0.432047	1.58E-31	0.417737	2.20E-29	0.287301
CERS5	TOPBP1	liver	0.637818	3.28E-27	0.60309	8.94E-24	0.436932
CERS5	UGCG	adipose_su	0.208644	5.89E-08	0.228502	2.66E-09	0.156334
CERS5	UGCG	liver	0.471464	6.59E-14	0.523006	2.88E-17	0.374199
CERS6	ACER2	adipose_su	0.30504	9.61E-16	0.311426	2.24E-16	0.211148
CERS6	ACER2	liver	0.548197	3.96E-19	0.690812	2.17E-33	0.498682
CERS6	CERS1	adipose_su	-0.15195	8.57E-05	-0.26933	1.76E-12	-0.18088
CERS6	CERS1	liver	0.114456	0.086023	-0.05666	0.39661	-0.0354
CERS6	SGMS1	adipose_su	-0.04154	0.285479	-0.10571	0.006443	-0.06461
CERS6	SGMS1	liver	0.101769	0.127158	0.033422	0.617218	0.026273
CERS6	SPHK1	adipose_su	-0.36737	1.30E-22	-0.56174	2.09E-56	-0.38621
CERS6	SPHK1	liver	0.121862	0.067453	0.283352	1.52E-05	0.197522
CERS6	SPTLC3	adipose_su	-0.13239	0.000632	-0.23718	6.25E-10	-0.15171
CERS6	SPTLC3	liver	0.401887	3.50E-10	0.325158	5.81E-07	0.235005
DEGS1	ACER1	adipose_su	0.627715	6.22E-74	0.00116	0.976224	0.000501
DEGS1	ACER1	liver	-0.1722	0.009493	-0.36715	1.28E-08	-0.24602
DEGS1	ACER2	adipose_su	0.060347	0.120583	0.071931	0.064163	0.048101
DEGS1	ACER2	liver	0.502852	6.92E-16	0.698729	1.98E-34	0.503992
DEGS1	B4GALT5	adipose_su	0.330239	2.47E-18	0.445132	1.40E-33	0.308043
DEGS1	B4GALT5	liver	0.7443	3.81E-41	0.798261	3.19E-51	0.606922
DEGS1	CBR3	adipose_su	-0.09596	0.013441	-0.11309	0.003546	-0.07568
DEGS1	CBR3	liver	0.289823	9.50E-06	0.210498	0.001459	0.142852
DEGS1	CERS1	adipose_su	0.007961	0.83789	0.030138	0.438496	0.020565
DEGS1	CERS1	liver	0.230989	0.000464	0.021264	0.750538	0.015536
DEGS1	CERS3	adipose_su	0.623781	9.08E-73	-0.02208	0.570273	-0.01541
DEGS1	CERS3	liver	0.076703	0.250805	0.127169	0.056271	0.084641
DEGS1	CERS6	adipose_su	0.19296	5.55E-07	0.169852	1.10E-05	0.113455
DEGS1	CERS6	liver	0.564836	1.91E-20	0.682801	2.26E-32	0.49471
DEGS1	DEGS2	adipose_su	0.504268	4.70E-44	-0.12405	0.001372	-0.08328

DEGS1	DEGS2	liver	0.011987	0.857767	-0.24551	0.000193	-0.16676
DEGS1	HKDC1	adipose_su	-0.0007	0.985736	-0.0524	0.177807	-0.03558
DEGS1	HKDC1	liver	0.224947	0.000658	0.362977	1.92E-08	0.241731
DEGS1	MAPK7	adipose_su	0.145072	0.000178	0.17066	9.94E-06	0.115227
DEGS1	MAPK7	liver	0.643003	9.23E-28	0.759832	9.01E-44	0.563107
DEGS1	SGMS1	adipose_su	0.242309	2.58E-10	0.213078	3.03E-08	0.144281
DEGS1	SGMS1	liver	-0.11389	0.087588	-0.15118	0.02301	-0.10521
DEGS1	SGMS2	adipose_su	0.305625	8.42E-16	0.366508	1.65E-22	0.251339
DEGS1	SGMS2	liver	0.392558	9.60E-10	0.672942	3.66E-31	0.491681
DEGS1	SGPL1	adipose_su	0.189273	9.16E-07	0.095091	0.014309	0.064784
DEGS1	SGPL1	liver	0.16681	0.012025	0.195492	0.003166	0.136047
DEGS1	SGPP2	adipose_su	0.509509	4.38E-45	0.157225	4.78E-05	0.107094
DEGS1	SGPP2	liver	0.258679	8.34E-05	0.223764	0.000703	0.147139
DEGS1	SMPD1	adipose_su	0.172343	8.09E-06	0.252367	4.31E-11	0.167676
DEGS1	SMPD1	liver	0.64519	5.37E-28	0.726999	1.95E-38	0.534277
DEGS1	SMSr	adipose_su	0.201015	1.79E-07	0.294747	9.35E-15	0.19966
DEGS1	SMSr	liver	0.711597	3.41E-36	0.752033	1.98E-42	0.561101
DEGS1	SPHK1	adipose_su	0.159936	3.52E-05	0.10776	0.005477	0.073223
DEGS1	SPHK1	liver	0.4113	1.23E-10	0.475899	3.56E-14	0.333176
DEGS1	SPTLC3	adipose_su	0.306861	6.36E-16	-0.00278	0.943072	-0.00074
DEGS1	SPTLC3	liver	0.123902	0.062957	0.121632	0.067975	0.098171
DEGS1	TOPBP1	adipose_su	0.208046	6.44E-08	0.253668	3.40E-11	0.174042
DEGS1	TOPBP1	liver	0.585507	3.44E-22	0.656658	2.90E-29	0.471504
DEGS1	UGCG	adipose_su	0.228786	2.54E-09	0.248352	8.90E-11	0.168259
DEGS1	UGCG	liver	0.413934	9.09E-11	0.403694	2.87E-10	0.278269
DEGS2	ACER2	adipose_su	-0.08308	0.032446	-0.18835	1.04E-06	-0.12685
DEGS2	ACER2	liver	0.005766	0.931309	-0.20233	0.00224	-0.12787
DEGS2	CERS1	adipose_su	0.074794	0.054238	0.279842	2.15E-13	0.18702
DEGS2	CERS1	liver	0.028173	0.673555	0.151789	0.022461	0.102498
DEGS2	CERS6	adipose_su	-0.04526	0.244516	-0.28759	4.32E-14	-0.19443
DEGS2	CERS6	liver	-0.0307	0.646153	-0.24095	0.000256	-0.1613
DEGS2	SGMS1	adipose_su	0.207738	6.74E-08	0.249683	7.01E-11	0.168587
DEGS2	SGMS1	liver	0.052807	0.429516	0.221805	0.000785	0.149931
DEGS2	SPHK1	adipose_su	0.046437	0.232446	0.259419	1.17E-11	0.170911
DEGS2	SPHK1	liver	-0.03153	0.637279	-0.01948	0.770906	-0.00944
DEGS2	SPTLC3	adipose_su	0.499137	4.61E-43	0.191745	6.55E-07	0.130484
DEGS2	SPTLC3	liver	-0.20073	0.00243	-0.17266	0.0093	-0.11465
EDA2R	ACER1	adipose_su	-0.00228	0.953243	0.205967	8.75E-08	0.136722
EDA2R	ACER1	liver	-0.04769	0.475588	-0.07305	0.274161	-0.04983
EDA2R	ACER2	adipose_su	-0.12421	0.001352	-0.19925	2.31E-07	-0.13463
EDA2R	ACER2	liver	0.422239	3.48E-11	0.245736	0.000191	0.174238
EDA2R	B4GALT5	adipose_su	0.12085	0.001825	0.170072	1.07E-05	0.114252
EDA2R	B4GALT5	liver	0.2038	0.002076	0.105829	0.112605	0.072488
EDA2R	CBR3	adipose_su	0.183846	1.88E-06	0.235212	8.72E-10	0.159269

EDA2R	CBR3	liver	0.296394	5.81E-06	0.231371	0.000454	0.153786
EDA2R	CDR2	adipose_su	0.375761	1.16E-23	0.444005	2.11E-33	0.303158
EDA2R	CDR2	liver	0.325523	5.63E-07	0.266942	4.81E-05	0.184503
EDA2R	CERS1	adipose_su	0.229728	2.17E-09	0.366894	1.48E-22	0.250254
EDA2R	CERS1	liver	0.327903	4.60E-07	0.096698	0.147328	0.062812
EDA2R	CERS2	adipose_su	0.192338	6.04E-07	0.166041	1.73E-05	0.112594
EDA2R	CERS2	liver	0.123464	0.0639	0.148644	0.02544	0.096834
EDA2R	CERS3	adipose_su	-0.00631	0.871131	0.169602	1.13E-05	0.112275
EDA2R	CERS3	liver	0.090646	0.174481	0.115757	0.082493	0.073707
EDA2R	CERS5	adipose_su	0.56214	1.68E-56	0.609128	1.42E-68	0.435966
EDA2R	CERS5	liver	0.318432	1.02E-06	0.313172	1.56E-06	0.216716
EDA2R	CERS6	adipose_su	-0.20463	1.06E-07	-0.37693	8.26E-24	-0.25242
EDA2R	CERS6	liver	0.22012	0.000863	0.20824	0.001645	0.139312
EDA2R	DEGS1	adipose_su	-0.03865	0.320351	-0.02544	0.513188	-0.01756
EDA2R	DEGS1	liver	0.144825	0.029509	0.158948	0.016781	0.107414
EDA2R	DEGS2	adipose_su	0.058801	0.130406	0.326749	5.84E-18	0.218794
EDA2R	DEGS2	liver	0.124241	0.062233	0.194247	0.003368	0.129558
EDA2R	HKDC1	adipose_su	0.21972	1.09E-08	0.282087	1.36E-13	0.191522
EDA2R	HKDC1	liver	0.701181	9.30E-35	0.476371	3.33E-14	0.331563
EDA2R	KCTD3	adipose_su	0.063488	0.102408	0.106417	0.006093	0.071191
EDA2R	KCTD3	liver	-0.1129	0.090403	-0.05611	0.401221	-0.03851
EDA2R	MAPK7	adipose_su	0.301022	2.36E-15	0.386353	4.98E-25	0.264088
EDA2R	MAPK7	liver	0.25894	8.20E-05	0.278114	2.22E-05	0.193707
EDA2R	SEMA4F	adipose_su	0.249461	7.29E-11	0.293506	1.22E-14	0.198995
EDA2R	SEMA4F	liver	0.525113	2.04E-17	0.455589	5.56E-13	0.316342
EDA2R	SGMS1	adipose_su	0.422713	4.05E-30	0.462243	2.10E-36	0.315826
EDA2R	SGMS1	liver	0.052139	0.435385	0.044369	0.506921	0.027021
EDA2R	SGMS2	adipose_su	0.376054	1.07E-23	0.419185	1.35E-29	0.288659
EDA2R	SGMS2	liver	-0.02634	0.693657	0.031436	0.638292	0.018997
EDA2R	SGPL1	adipose_su	-0.05411	0.164031	-0.13746	0.000386	-0.08976
EDA2R	SGPL1	liver	0.079966	0.231149	0.106704	0.109644	0.073589
EDA2R	SGPP2	adipose_su	-0.04191	0.281268	-0.01297	0.73888	-0.00856
EDA2R	SGPP2	liver	0.066668	0.318381	-0.05625	0.399989	-0.03709
EDA2R	SMPD1	adipose_su	0.203756	1.21E-07	0.217414	1.56E-08	0.148301
EDA2R	SMPD1	liver	0.116742	0.079899	0.155685	0.019192	0.104621
EDA2R	SMPD2	adipose_su	0.343719	8.03E-20	0.331183	1.95E-18	0.225046
EDA2R	SMPD2	liver	0.251503	0.000133	0.29912	4.72E-06	0.203815
EDA2R	SMSr	adipose_su	0.00205	0.957992	-0.05668	0.144875	-0.0388
EDA2R	SMSr	liver	0.27702	2.40E-05	0.258929	8.20E-05	0.175654
EDA2R	SPHK1	adipose_su	0.372826	2.72E-23	0.538513	3.99E-51	0.374317
EDA2R	SPHK1	liver	0.21415	0.001199	0.248322	0.000162	0.172861
EDA2R	SPTLC3	adipose_su	0.295122	8.62E-15	0.403441	2.42E-27	0.276364
EDA2R	SPTLC3	liver	-0.01048	0.875475	0.079921	0.231412	0.053019
EDA2R	TOPBP1	adipose_su	0.229466	2.27E-09	0.2695	1.70E-12	0.181077

EDA2R	TOPBP1	liver	0.227515	0.000568	0.286426	1.22E-05	0.196657
EDA2R	UGCG	adipose_su	0.212025	3.55E-08	0.271642	1.12E-12	0.181109
EDA2R	UGCG	liver	0.332049	3.22E-07	0.220423	0.000849	0.147217
HKDC1	ACER1	adipose_su	0.128117	0.000945	0.202115	1.53E-07	0.136129
HKDC1	ACER1	liver	-0.05381	0.420774	-0.26054	7.38E-05	-0.17573
HKDC1	ACER2	adipose_su	-0.14679	0.000149	-0.21973	1.08E-08	-0.14815
HKDC1	ACER2	liver	0.468999	9.24E-14	0.474601	4.27E-14	0.335929
HKDC1	B4GALT5	adipose_su	-0.05797	0.135967	-0.06294	0.105383	-0.0417
HKDC1	B4GALT5	liver	0.305245	2.93E-06	0.368576	1.11E-08	0.252586
HKDC1	CBR3	adipose_su	0.18609	1.40E-06	0.235376	8.48E-10	0.158107
HKDC1	CBR3	liver	0.275849	2.60E-05	0.365053	1.57E-08	0.249007
HKDC1	CERS1	adipose_su	0.73527	9.37E-114	0.315293	9.09E-17	0.218657
HKDC1	CERS1	liver	0.2861	1.25E-05	0.08599	0.197768	0.053255
HKDC1	CERS6	adipose_su	-0.10069	0.009478	-0.0548	0.158715	-0.03642
HKDC1	CERS6	liver	0.343517	1.17E-07	0.4714	6.65E-14	0.328456
HKDC1	DEGS2	adipose_su	0.150925	9.57E-05	0.235391	8.46E-10	0.160326
HKDC1	DEGS2	liver	0.075734	0.256861	-0.03664	0.583683	-0.02836
HKDC1	MAPK7	adipose_su	0.214428	2.46E-08	0.261704	7.62E-12	0.17586
HKDC1	MAPK7	liver	0.281674	1.72E-05	0.483556	1.20E-14	0.334238
HKDC1	SGMS1	adipose_su	0.225311	4.46E-09	0.274505	6.32E-13	0.183748
HKDC1	SGMS1	liver	0.046029	0.491138	0.096828	0.146784	0.066313
HKDC1	SGMS2	adipose_su	0.188605	1.00E-06	0.23098	1.77E-09	0.155541
HKDC1	SGMS2	liver	0.033531	0.616067	0.296264	5.86E-06	0.198623
HKDC1	SGPL1	adipose_su	0.057431	0.139622	0.160656	3.24E-05	0.10806
HKDC1	SGPL1	liver	0.144029	0.030425	0.226461	0.000603	0.14765
HKDC1	SGPP2	adipose_su	0.117604	0.002422	0.163967	2.21E-05	0.110124
HKDC1	SGPP2	liver	0.118063	0.076521	0.131773	0.047857	0.090383
HKDC1	SMPD1	adipose_su	0.167495	1.46E-05	0.241035	3.22E-10	0.161711
HKDC1	SMPD1	liver	0.212834	0.001287	0.419803	4.62E-11	0.283854
HKDC1	SMSr	adipose_su	-0.07453	0.055089	-0.00898	0.817402	-0.00729
HKDC1	SMSr	liver	0.309475	2.10E-06	0.47221	5.94E-14	0.330187
HKDC1	SPHK1	adipose_su	0.171543	8.93E-06	0.211213	4.01E-08	0.143612
HKDC1	SPHK1	liver	0.20756	0.001705	0.408178	1.74E-10	0.2894
HKDC1	SPTLC3	adipose_su	0.301569	2.09E-15	0.185179	1.58E-06	0.125886
HKDC1	SPTLC3	liver	0.029266	0.661662	0.202661	0.002202	0.133609
HKDC1	TOPBP1	adipose_su	0.105165	0.006723	0.235812	7.88E-10	0.161214
HKDC1	TOPBP1	liver	0.313373	1.54E-06	0.526547	1.61E-17	0.366372
KCTD3	ACER1	adipose_su	0.038701	0.31974	0.133055	0.000593	0.090817
KCTD3	ACER1	liver	0.032148	0.630703	0.077793	0.244112	0.052665
KCTD3	ACER2	adipose_su	0.075622	0.05162	0.058196	0.134413	0.039024
KCTD3	ACER2	liver	-0.04995	0.454961	-0.0179	0.789014	-0.0092
KCTD3	B4GALT5	adipose_su	0.123672	0.001419	0.156006	5.48E-05	0.105959
KCTD3	B4GALT5	liver	-0.01688	0.800789	0.002973	0.964546	0.00822
KCTD3	CBR3	adipose_su	0.04866	0.210816	0.102784	0.008083	0.067741

KCTD3	CBR3	liver	0.097017	0.145994	0.108452	0.103917	0.069066
KCTD3	CDR2	adipose_su	0.038257	0.325329	0.076759	0.048197	0.051915
KCTD3	CDR2	liver	0.060852	0.362516	0.08232	0.21766	0.057542
KCTD3	CERS1	adipose_su	0.02213	0.569482	-0.00295	0.939552	-0.00086
KCTD3	CERS1	liver	-0.15072	0.023438	-0.15333	0.021114	-0.10175
KCTD3	CERS2	adipose_su	0.362819	4.66E-22	0.421275	6.63E-30	0.293215
KCTD3	CERS2	liver	0.421549	3.77E-11	0.387867	1.57E-09	0.27351
KCTD3	CERS3	adipose_su	0.035143	0.36628	0.090444	0.019849	0.062173
KCTD3	CERS3	liver	0.054152	0.417843	0.128451	0.053814	0.082832
KCTD3	CERS5	adipose_su	0.308984	3.92E-16	0.327526	4.83E-18	0.222927
KCTD3	CERS5	liver	-0.07769	0.244722	-0.05124	0.443385	-0.03091
KCTD3	CERS6	adipose_su	0.349967	1.55E-20	0.38618	5.25E-25	0.258935
KCTD3	CERS6	liver	0.312769	1.61E-06	0.227044	0.000583	0.156303
KCTD3	DEGS1	adipose_su	0.194922	4.23E-07	0.237018	6.42E-10	0.161497
KCTD3	DEGS1	liver	0.020449	0.759801	0.01239	0.853047	0.017974
KCTD3	DEGS2	adipose_su	0.058253	0.134034	0.093274	0.016288	0.062524
KCTD3	DEGS2	liver	0.000914	0.989096	0.07449	0.264779	0.053569
KCTD3	HKDC1	adipose_su	0.087316	0.024555	0.226826	3.49E-09	0.153363
KCTD3	HKDC1	liver	-0.08409	0.207888	0.025324	0.704951	0.018328
KCTD3	MAPK7	adipose_su	0.269339	1.75E-12	0.285489	6.70E-14	0.1956
KCTD3	MAPK7	liver	-0.0425	0.524982	-0.04751	0.477281	-0.01931
KCTD3	SGMS1	adipose_su	0.258387	1.42E-11	0.31432	1.14E-16	0.214784
KCTD3	SGMS1	liver	0.579784	1.07E-21	0.549669	3.05E-19	0.391386
KCTD3	SGMS2	adipose_su	0.315568	8.52E-17	0.339989	2.11E-19	0.230295
KCTD3	SGMS2	liver	0.080964	0.225361	0.247804	0.000168	0.184425
KCTD3	SGPL1	adipose_su	0.328357	3.93E-18	0.366912	1.48E-22	0.253963
KCTD3	SGPL1	liver	0.461635	2.50E-13	0.443809	2.52E-12	0.318466
KCTD3	SGPP2	adipose_su	0.127746	0.000979	0.229827	2.14E-09	0.155505
KCTD3	SGPP2	liver	0.000915	0.98909	0.120347	0.070957	0.081455
KCTD3	SMPD1	adipose_su	0.194944	4.22E-07	0.192058	6.28E-07	0.130502
KCTD3	SMPD1	liver	0.099482	0.135976	0.085343	0.201176	0.061986
KCTD3	SMSr	adipose_su	0.508419	7.20E-45	0.56906	3.70E-58	0.400806
KCTD3	SMSr	liver	0.12515	0.06033	0.129399	0.052054	0.101003
KCTD3	SPHK1	adipose_su	-0.03229	0.406506	-0.04784	0.218604	-0.03399
KCTD3	SPHK1	liver	-0.08221	0.218267	-0.22153	0.000797	-0.15414
KCTD3	SPTLC3	adipose_su	0.047917	0.217881	0.049601	0.202119	0.032968
KCTD3	SPTLC3	liver	0.555927	9.88E-20	0.487261	7.05E-15	0.344228
KCTD3	TOPBP1	adipose_su	0.692235	1.03E-95	0.688547	2.58E-94	0.505439
KCTD3	TOPBP1	liver	0.300359	4.29E-06	0.254452	0.00011	0.177856
KCTD3	UGCG	adipose_su	-0.06058	0.119145	-0.01249	0.748166	-0.01097
KCTD3	UGCG	liver	-0.05073	0.447885	0.014885	0.823886	0.007827
KDSR	ACER1	adipose_su	0.094959	0.014445	0.162823	2.52E-05	0.108975
KDSR	ACER1	liver	-0.16502	0.012991	-0.34748	8.19E-08	-0.23929
KDSR	ACER2	adipose_su	0.07867	0.042871	0.065188	0.093519	0.04375

KDSR	ACER2	liver	0.416893	6.47E-11	0.63655	4.46E-27	0.458879
KDSR	B4GALT5	adipose_su	0.272397	9.61E-13	0.261291	8.24E-12	0.176484
KDSR	B4GALT5	liver	0.810539	5.93E-54	0.813435	1.26E-54	0.623953
KDSR	CBR3	adipose_su	0.259599	1.13E-11	0.283618	9.89E-14	0.193112
KDSR	CBR3	liver	0.13581	0.041369	0.105801	0.112699	0.0706
KDSR	CDR2	adipose_su	0.316027	7.65E-17	0.266511	3.04E-12	0.180339
KDSR	CDR2	liver	0.549691	3.04E-19	0.484745	1.01E-14	0.34297
KDSR	CERS1	adipose_su	0.120087	0.001952	0.159434	3.73E-05	0.106524
KDSR	CERS1	liver	0.171467	0.009806	-0.02097	0.753829	-0.01593
KDSR	CERS2	adipose_su	0.775772	2.67E-134	0.77471	1.05E-133	0.583779
KDSR	CERS2	liver	0.460083	3.07E-13	0.559219	5.41E-20	0.404051
KDSR	CERS3	adipose_su	0.093841	0.015646	0.211101	4.08E-08	0.142814
KDSR	CERS3	liver	0.044527	0.505409	0.12942	0.052017	0.084838
KDSR	CERS5	adipose_su	0.562348	1.50E-56	0.530555	2.07E-49	0.369706
KDSR	CERS5	liver	0.541878	1.20E-18	0.490471	4.42E-15	0.349931
KDSR	CERS6	adipose_su	0.240419	3.59E-10	0.250929	5.60E-11	0.168341
KDSR	CERS6	liver	0.469427	8.71E-14	0.626365	4.98E-26	0.455969
KDSR	DEGS1	adipose_su	0.283505	1.01E-13	0.320784	2.47E-17	0.218024
KDSR	DEGS1	liver	0.75565	4.80E-43	0.812043	2.66E-54	0.619626
KDSR	DEGS2	adipose_su	0.150733	9.77E-05	0.202707	1.41E-07	0.136211
KDSR	DEGS2	liver	0.112582	0.091322	-0.20741	0.001719	-0.13809
KDSR	EDA2R	adipose_su	0.290292	2.43E-14	0.26131	8.21E-12	0.178972
KDSR	EDA2R	liver	0.149568	0.024532	0.11591	0.082085	0.077325
KDSR	HKDC1	adipose_su	0.093615	0.0159	0.17948	3.31E-06	0.119748
KDSR	HKDC1	liver	0.224442	0.000677	0.321601	7.82E-07	0.216598
KDSR	KCTD3	adipose_su	0.49788	8.03E-43	0.553961	1.37E-54	0.394007
KDSR	KCTD3	liver	-0.00862	0.897483	0.060372	0.366326	0.053727
KDSR	MAPK7	adipose_su	0.377803	6.38E-24	0.405109	1.41E-27	0.278821
KDSR	MAPK7	liver	0.509509	2.48E-16	0.608301	2.90E-24	0.438151
KDSR	SEMA4F	adipose_su	0.086233	0.026395	0.063528	0.102186	0.042747
KDSR	SEMA4F	liver	0.133913	0.04432	0.201115	0.002384	0.140177
KDSR	SGMS1	adipose_su	0.412335	1.33E-28	0.407838	5.84E-28	0.281445
KDSR	SGMS1	liver	-0.05081	0.447233	-0.09521	0.153697	-0.06191
KDSR	SGMS2	adipose_su	0.357282	2.15E-21	0.362122	5.66E-22	0.246887
KDSR	SGMS2	liver	0.644178	6.90E-28	0.783956	2.85E-48	0.593432
KDSR	SGPL1	adipose_su	0.227602	3.08E-09	0.259655	1.12E-11	0.176639
KDSR	SGPL1	liver	0.112225	0.09236	0.185913	0.005049	0.140964
KDSR	SGPP1	adipose_su	0.50199	1.30E-43	0.523136	7.52E-48	0.366912
KDSR	SGPP1	liver	0.678927	6.85E-32	0.784278	2.46E-48	0.585015
KDSR	SGPP2	adipose_su	0.110252	0.004481	0.087116	0.024886	0.05824
KDSR	SGPP2	liver	0.317748	1.08E-06	0.211862	0.001356	0.14175
KDSR	SMPD1	adipose_su	0.326757	5.83E-18	0.342116	1.22E-19	0.237354
KDSR	SMPD1	liver	0.496418	1.83E-15	0.667264	1.74E-30	0.484208
KDSR	SMPD2	adipose_su	0.238404	5.07E-10	0.245844	1.39E-10	0.167024

KDSR	SMPD2	liver	0.619166	2.60E-25	0.594906	5.02E-23	0.438033
KDSR	SMSr	adipose_su	0.498623	5.79E-43	0.51032	3.02E-45	0.357029
KDSR	SMSr	liver	0.703088	5.13E-35	0.710388	5.05E-36	0.534159
KDSR	SPHK1	adipose_su	0.254461	2.94E-11	0.196723	3.29E-07	0.133359
KDSR	SPHK1	liver	0.482479	1.40E-14	0.430551	1.29E-11	0.300492
KDSR	SPTLC3	adipose_su	0.03123	0.422085	0.0149	0.701759	0.009606
KDSR	SPTLC3	liver	-0.11295	0.090271	-0.0418	0.531881	-0.01546
KDSR	TOPBP1	adipose_su	0.552539	2.90E-54	0.564068	5.86E-57	0.401466
KDSR	TOPBP1	liver	0.501734	8.21E-16	0.574552	2.99E-21	0.415379
KDSR	UGCG	adipose_su	0.100472	0.009634	0.079567	0.040547	0.054007
KDSR	UGCG	liver	0.373165	7.07E-09	0.433538	9.00E-12	0.299233
MAPK7	ACER1	adipose_su	0.06894	0.076084	0.219271	1.16E-08	0.148264
MAPK7	ACER1	liver	-0.12679	0.057008	-0.30761	2.43E-06	-0.2155
MAPK7	ACER2	adipose_su	-0.15647	5.21E-05	-0.16792	1.38E-05	-0.112
MAPK7	ACER2	liver	0.473565	4.93E-14	0.67923	6.28E-32	0.488102
MAPK7	CERS1	adipose_su	0.234057	1.06E-09	0.239492	4.21E-10	0.163903
MAPK7	CERS1	liver	0.564878	1.89E-20	0.117017	0.079186	0.07587
MAPK7	CERS6	adipose_su	-0.11437	0.003188	-0.12483	0.001278	-0.08265
MAPK7	CERS6	liver	0.428929	1.57E-11	0.554726	1.23E-19	0.390796
MAPK7	DEGS2	adipose_su	0.143903	0.000201	0.266981	2.77E-12	0.180389
MAPK7	DEGS2	liver	0.02373	0.72273	-0.18736	0.004712	-0.12791
MAPK7	SGMS1	adipose_su	0.181315	2.62E-06	0.230457	1.93E-09	0.155919
MAPK7	SGMS1	liver	0.000113	0.998657	-0.0876	0.189479	-0.05974
MAPK7	SPHK1	adipose_su	0.359535	1.16E-21	0.432827	1.20E-31	0.298428
MAPK7	SPHK1	liver	0.549945	2.90E-19	0.59839	2.42E-23	0.426627
MAPK7	SPTLC3	adipose_su	0.007922	0.838661	0.02511	0.518647	0.017083
MAPK7	SPTLC3	liver	-0.04293	0.5208	0.042633	0.523703	0.05058
SEMA4F	ACER1	adipose_su	-0.00258	0.947035	-0.05319	0.171318	-0.0357
SEMA4F	ACER1	liver	-0.06439	0.335198	-0.09135	0.171136	-0.05951
SEMA4F	ACER2	adipose_su	0.075995	0.050474	0.070123	0.071168	0.047058
SEMA4F	ACER2	liver	0.395365	7.11E-10	0.319882	9.02E-07	0.219076
SEMA4F	B4GALT5	adipose_su	-0.05524	0.155354	-0.00545	0.888598	-0.0015
SEMA4F	B4GALT5	liver	0.201275	0.002364	0.239829	0.000274	0.170423
SEMA4F	CBR3	adipose_su	0.146085	0.00016	0.161706	2.87E-05	0.110005
SEMA4F	CBR3	liver	0.508736	2.80E-16	0.417674	5.92E-11	0.290973
SEMA4F	CDR2	adipose_su	0.033247	0.392727	0.03992	0.304723	0.027304
SEMA4F	CDR2	liver	0.33433	2.65E-07	0.260879	7.22E-05	0.178053
SEMA4F	CERS1	adipose_su	0.058149	0.13473	0.126235	0.001125	0.085349
SEMA4F	CERS1	liver	0.392044	1.01E-09	0.133588	0.044842	0.084051
SEMA4F	CERS2	adipose_su	0.207528	6.95E-08	0.169652	1.12E-05	0.115341
SEMA4F	CERS2	liver	0.307829	2.39E-06	0.358908	2.83E-08	0.250541
SEMA4F	CERS3	adipose_su	0.017161	0.659161	0.246332	1.28E-10	0.166036
SEMA4F	CERS3	liver	0.049361	0.460277	0.122895	0.065143	0.083382
SEMA4F	CERS5	adipose_su	0.096293	0.013119	0.090118	0.020299	0.061202

SEMA4F	CERS5	liver	0.50335	6.42E-16	0.53993	1.68E-18	0.384346
SEMA4F	CERS6	adipose_su	0.036046	0.354088	-0.01922	0.621297	-0.00881
SEMA4F	CERS6	liver	0.402983	3.10E-10	0.42834	1.69E-11	0.302616
SEMA4F	DEGS1	adipose_su	-0.00454	0.907163	-0.04624	0.234425	-0.03158
SEMA4F	DEGS1	liver	0.264914	5.51E-05	0.311303	1.81E-06	0.211013
SEMA4F	DEGS2	adipose_su	-0.00347	0.928935	0.011003	0.777339	0.007792
SEMA4F	DEGS2	liver	0.132471	0.046678	0.194744	0.003286	0.130659
SEMA4F	HKDC1	adipose_su	0.027065	0.486616	0.072558	0.061873	0.047741
SEMA4F	HKDC1	liver	0.52301	2.87E-17	0.498942	1.25E-15	0.347296
SEMA4F	KCTD3	adipose_su	-0.09076	0.019415	-0.10015	0.009868	-0.06892
SEMA4F	KCTD3	liver	0.044339	0.507203	0.120226	0.071243	0.08177
SEMA4F	MAPK7	adipose_su	-0.10916	0.004895	-0.07256	0.061874	-0.04864
SEMA4F	MAPK7	liver	0.470394	7.63E-14	0.410712	1.31E-10	0.286293
SEMA4F	SGMS1	adipose_su	0.432591	1.30E-31	0.434662	6.23E-32	0.29641
SEMA4F	SGMS1	liver	0.169929	0.010495	0.272256	3.34E-05	0.183559
SEMA4F	SGMS2	adipose_su	0.137477	0.000385	0.176193	5.02E-06	0.119971
SEMA4F	SGMS2	liver	-0.04667	0.485074	0.201283	0.002363	0.144464
SEMA4F	SGPL1	adipose_su	0.259357	1.19E-11	0.245207	1.56E-10	0.165767
SEMA4F	SGPL1	liver	0.389948	1.27E-09	0.443421	2.65E-12	0.312724
SEMA4F	SGPP2	adipose_su	-0.12515	0.001242	-0.2143	2.51E-08	-0.14651
SEMA4F	SGPP2	liver	0.035011	0.600572	-0.02617	0.69559	-0.01137
SEMA4F	SMPD1	adipose_su	0.105229	0.006689	0.115861	0.002811	0.077748
SEMA4F	SMPD1	liver	0.328295	4.45E-07	0.415822	7.32E-11	0.284366
SEMA4F	SMSr	adipose_su	0.116139	0.002745	0.083234	0.032125	0.05536
SEMA4F	SMSr	liver	0.40279	3.17E-10	0.460644	2.85E-13	0.322517
SEMA4F	SPHK1	adipose_su	-0.1458	0.000165	-0.09847	0.011182	-0.06656
SEMA4F	SPHK1	liver	0.180084	0.006638	0.275778	2.61E-05	0.197483
SEMA4F	SPTLC3	adipose_su	0.39911	9.61E-27	0.481197	1.01E-39	0.334659
SEMA4F	SPTLC3	liver	0.1619	0.01483	0.237419	0.000317	0.165349
SEMA4F	TOPBP1	adipose_su	0.100085	0.009918	0.10782	0.005451	0.073442
SEMA4F	TOPBP1	liver	0.480213	1.94E-14	0.52064	4.23E-17	0.368771
SEMA4F	UGCG	adipose_su	-0.05758	0.138571	-0.01145	0.768631	-0.00592
SEMA4F	UGCG	liver	0.239476	0.00028	0.290766	8.86E-06	0.207866
SGMS1	CERS1	adipose_su	0.173564	6.96E-06	0.187981	1.09E-06	0.125676
SGMS1	CERS1	liver	0.041034	0.539402	0.070392	0.292037	0.04468
SGMS2	ACER1	adipose_su	0.031029	0.425077	0.096032	0.01337	0.064597
SGMS2	ACER1	liver	-0.1142	0.086742	-0.32202	7.55E-07	-0.21605
SGMS2	ACER2	adipose_su	-0.23386	1.09E-09	-0.28386	9.40E-14	-0.18834
SGMS2	ACER2	liver	0.207828	0.001681	0.504734	5.19E-16	0.352094
SGMS2	CERS1	adipose_su	0.196405	3.44E-07	0.197879	2.80E-07	0.134357
SGMS2	CERS1	liver	0.176414	0.007855	0.03402	0.610934	0.021278
SGMS2	CERS6	adipose_su	-0.02518	0.517448	-0.11362	0.003395	-0.07358
SGMS2	CERS6	liver	0.190884	0.003974	0.595238	4.69E-23	0.427375
SGMS2	DEGS2	adipose_su	0.027121	0.485721	0.075822	0.051004	0.050457

SGMS2	DEGS2	liver	0.035789	0.592502	-0.16408	0.013521	-0.10855
SGMS2	MAPK7	adipose_su	0.312198	1.87E-16	0.389652	1.83E-25	0.266768
SGMS2	MAPK7	liver	0.286217	1.24E-05	0.530039	9.01E-18	0.376677
SGMS2	SGMS1	adipose_su	0.446189	9.44E-34	0.517018	1.36E-46	0.367227
SGMS2	SGMS1	liver	0.025117	0.707241	0.135393	0.042003	0.093097
SGMS2	SGPL1	adipose_su	0.146793	0.000149	0.174626	6.11E-06	0.117333
SGMS2	SGPL1	liver	-0.14117	0.033915	0.241102	0.000254	0.18765
SGMS2	SMPD1	adipose_su	0.378308	5.50E-24	0.460388	4.32E-36	0.318806
SGMS2	SMPD1	liver	0.112498	0.091564	0.535085	3.85E-18	0.376873
SGMS2	SPHK1	adipose_su	0.403361	2.48E-27	0.288061	3.90E-14	0.197231
SGMS2	SPHK1	liver	0.495547	2.08E-15	0.451762	9.14E-13	0.315477
SGMS2	SPTLC3	adipose_su	0.23119	1.71E-09	0.326654	5.98E-18	0.226121
SGMS2	SPTLC3	liver	-0.1926	0.003653	0.017099	0.798225	0.039489
SGPL1	ACER1	adipose_su	0.216731	1.73E-08	0.032428	0.404486	0.021836
SGPL1	ACER1	liver	0.084071	0.207997	0.02353	0.724975	0.015143
SGPL1	ACER2	adipose_su	0.160247	3.40E-05	0.192575	5.85E-07	0.129121
SGPL1	ACER2	liver	0.025375	0.704374	0.067513	0.31227	0.050108
SGPL1	CERS1	adipose_su	-0.06961	0.073265	-0.12611	0.001138	-0.08305
SGPL1	CERS1	liver	0.061734	0.355586	-0.02345	0.725866	-0.01794
SGPL1	CERS6	adipose_su	0.494456	3.59E-42	0.562453	1.42E-56	0.390006
SGPL1	CERS6	liver	0.38334	2.52E-09	0.335011	2.49E-07	0.229774
SGPL1	DEGS2	adipose_su	0.199286	2.30E-07	-0.0067	0.86319	-0.00324
SGPL1	DEGS2	liver	0.084704	0.204586	0.078194	0.241686	0.053019
SGPL1	MAPK7	adipose_su	0.009558	0.805957	0.024799	0.523841	0.018213
SGPL1	MAPK7	liver	0.292684	7.68E-06	0.235505	0.000355	0.167434
SGPL1	SGMS1	adipose_su	0.137394	0.000388	0.128105	0.000947	0.086542
SGPL1	SGMS1	liver	0.382124	2.86E-09	0.370822	8.92E-09	0.25412
SGPL1	SMPD1	adipose_su	0.199594	2.20E-07	0.234113	1.05E-09	0.158262
SGPL1	SMPD1	liver	0.346241	9.17E-08	0.379227	3.84E-09	0.264621
SGPL1	SPHK1	adipose_su	-0.26141	8.05E-12	-0.38521	7.05E-25	-0.26384
SGPL1	SPHK1	liver	-0.05035	0.451332	-0.09364	0.160614	-0.0658
SGPL1	SPTLC3	adipose_su	0.210374	4.55E-08	0.078425	0.043525	0.052772
SGPL1	SPTLC3	liver	0.421123	3.96E-11	0.397145	5.87E-10	0.288456
SGPP1	ACER1	adipose_su	-0.04398	0.258165	0.1231	0.001494	0.083526
SGPP1	ACER1	liver	-0.14813	0.025963	-0.30519	2.95E-06	-0.21015
SGPP1	ACER2	adipose_su	0.00283	0.942014	-0.01537	0.692853	-0.00883
SGPP1	ACER2	liver	0.331442	3.40E-07	0.526886	1.52E-17	0.359882
SGPP1	B4GALT5	adipose_su	0.381334	2.25E-24	0.431208	2.12E-31	0.302311
SGPP1	B4GALT5	liver	0.70544	2.45E-35	0.710057	5.62E-36	0.520079
SGPP1	CBR3	adipose_su	0.097814	0.01174	0.12429	0.001343	0.082687
SGPP1	CBR3	liver	-0.01687	0.800868	-0.00387	0.953867	-0.00346
SGPP1	CDR2	adipose_su	0.185236	1.57E-06	0.21107	4.10E-08	0.142924
SGPP1	CDR2	liver	0.357894	3.11E-08	0.408844	1.62E-10	0.283776
SGPP1	CERS1	adipose_su	0.051721	0.183475	0.064254	0.098321	0.043631

SGPP1	CERS1	liver	0.082758	0.215216	-0.06304	0.34551	-0.04637
SGPP1	CERS2	adipose_su	0.399024	9.88E-27	0.428223	6.04E-31	0.290527
SGPP1	CERS2	liver	0.512718	1.50E-16	0.502383	7.44E-16	0.351858
SGPP1	CERS3	adipose_su	-0.04968	0.201387	0.09678	0.012663	0.065527
SGPP1	CERS3	liver	0.082558	0.216328	0.164596	0.013226	0.113589
SGPP1	CERS5	adipose_su	0.332178	1.53E-18	0.343264	9.04E-20	0.235686
SGPP1	CERS5	liver	0.322492	7.26E-07	0.373053	7.15E-09	0.253845
SGPP1	CERS6	adipose_su	0.190547	7.71E-07	0.164474	2.08E-05	0.110434
SGPP1	CERS6	liver	0.498793	1.28E-15	0.627787	3.57E-26	0.440433
SGPP1	DEGS1	adipose_su	0.380795	2.64E-24	0.518645	6.33E-47	0.361348
SGPP1	DEGS1	liver	0.712539	2.51E-36	0.769348	1.76E-45	0.574671
SGPP1	DEGS2	adipose_su	-0.04467	0.250694	0.037158	0.339427	0.023736
SGPP1	DEGS2	liver	0.025355	0.7046	-0.30793	2.37E-06	-0.2142
SGPP1	EDA2R	adipose_su	0.159409	3.74E-05	0.177498	4.26E-06	0.12034
SGPP1	EDA2R	liver	0.042512	0.524879	0.101465	0.128303	0.066352
SGPP1	HKDC1	adipose_su	0.070278	0.070547	0.14362	0.000207	0.094918
SGPP1	HKDC1	liver	0.091379	0.171007	0.279742	1.98E-05	0.183756
SGPP1	KCTD3	adipose_su	0.487129	8.33E-41	0.529628	3.26E-49	0.372367
SGPP1	KCTD3	liver	0.115886	0.08215	0.088698	0.183966	0.064621
SGPP1	MAPK7	adipose_su	0.219356	1.15E-08	0.254421	2.96E-11	0.17319
SGPP1	MAPK7	liver	0.388354	1.50E-09	0.577092	1.82E-21	0.4035
SGPP1	SEMA4F	adipose_su	0.111886	0.00392	0.068526	0.077865	0.047719
SGPP1	SEMA4F	liver	0.094901	0.155032	0.169657	0.010621	0.112134
SGPP1	SGMS1	adipose_su	0.539383	2.57E-51	0.565167	3.21E-57	0.40194
SGPP1	SGMS1	liver	-0.05877	0.379162	-0.10825	0.104564	-0.07461
SGPP1	SGMS2	adipose_su	0.599914	4.77E-66	0.614208	5.28E-70	0.440527
SGPP1	SGMS2	liver	0.288209	1.07E-05	0.69926	1.68E-34	0.511111
SGPP1	SGPL1	adipose_su	0.263407	5.51E-12	0.304487	1.09E-15	0.209193
SGPP1	SGPL1	liver	0.223143	0.000728	0.200546	0.002454	0.137778
SGPP1	SGPP2	adipose_su	0.046415	0.232671	0.199597	2.20E-07	0.133997
SGPP1	SGPP2	liver	0.08227	0.217942	0.058715	0.379648	0.036853
SGPP1	SMPD1	adipose_su	0.332038	1.58E-18	0.359077	1.31E-21	0.242744
SGPP1	SMPD1	liver	0.617481	3.80E-25	0.620575	1.89E-25	0.444366
SGPP1	SMPD2	adipose_su	0.140613	0.000281	0.169709	1.12E-05	0.114599
SGPP1	SMPD2	liver	0.444984	2.17E-12	0.541446	1.29E-18	0.377738
SGPP1	SMSr	adipose_su	0.437098	2.60E-32	0.46173	2.57E-36	0.320132
SGPP1	SMSr	liver	0.594787	5.15E-23	0.652097	9.39E-29	0.463795
SGPP1	SPHK1	adipose_su	0.131408	0.000694	0.095384	0.014011	0.064027
SGPP1	SPHK1	liver	0.106111	0.111646	0.269666	3.99E-05	0.18293
SGPP1	SPTLC3	adipose_su	0.170894	9.66E-06	0.240348	3.63E-10	0.1629
SGPP1	SPTLC3	liver	0.155986	0.018958	0.109424	0.100836	0.078584
SGPP1	TOPBP1	adipose_su	0.577244	3.61E-60	0.575912	7.74E-60	0.409245
SGPP1	TOPBP1	liver	0.427719	1.82E-11	0.524182	2.37E-17	0.360197
SGPP1	UGCG	adipose_su	0.132788	0.000608	0.212874	3.12E-08	0.14604

SGPP1	UGCG	liver	0.236111	0.000343	0.307409	2.47E-06	0.211249
SGPP2	ACER1	adipose_su	0.715354	5.48E-105	0.163208	2.41E-05	0.110671
SGPP2	ACER1	liver	0.115001	0.084531	-0.1028	0.123344	-0.06714
SGPP2	ACER2	adipose_su	-0.15528	5.94E-05	-0.31668	6.56E-17	-0.21554
SGPP2	ACER2	liver	0.061996	0.35355	0.148819	0.025267	0.101396
SGPP2	CERS1	adipose_su	0.031587	0.416789	0.078276	0.043927	0.05202
SGPP2	CERS1	liver	0.17417	0.008692	0.085208	0.201893	0.055969
SGPP2	CERS6	adipose_su	0.00241	0.95061	-0.08646	0.026005	-0.05742
SGPP2	CERS6	liver	0.04617	0.489809	0.091931	0.168425	0.057581
SGPP2	DEGS2	adipose_su	0.76763	7.79E-130	0.269567	1.68E-12	0.184541
SGPP2	DEGS2	liver	0.09521	0.153686	0.053161	0.426428	0.036735
SGPP2	MAPK7	adipose_su	0.089733	0.020843	0.1106	0.004355	0.074358
SGPP2	MAPK7	liver	0.290199	9.24E-06	0.254147	0.000112	0.174907
SGPP2	SGMS1	adipose_su	0.071783	0.064717	0.007823	0.840655	0.003873
SGPP2	SGMS1	liver	-0.16275	0.014305	-0.05773	0.387692	-0.04031
SGPP2	SGMS2	adipose_su	0.127534	0.000998	0.227391	3.19E-09	0.152543
SGPP2	SGMS2	liver	0.337128	2.07E-07	0.25344	0.000117	0.16531
SGPP2	SGPL1	adipose_su	0.229048	2.43E-09	0.133429	0.000572	0.089468
SGPP2	SGPL1	liver	0.077351	0.246812	0.165447	0.012752	0.111819
SGPP2	SMPD1	adipose_su	0.125053	0.001253	0.253179	3.72E-11	0.171845
SGPP2	SMPD1	liver	0.022767	0.733553	0.053641	0.422262	0.036578
SGPP2	SPHK1	adipose_su	0.12978	0.000809	0.107404	0.005635	0.072562
SGPP2	SPHK1	liver	0.562372	3.02E-20	0.332565	3.08E-07	0.231072
SGPP2	SPTLC3	adipose_su	0.328876	3.46E-18	-0.03993	0.304657	-0.02523
SGPP2	SPTLC3	liver	-0.14642	0.027751	-0.09471	0.155888	-0.05876
SGPP2	TOPBP1	adipose_su	0.183184	2.05E-06	0.323807	1.19E-17	0.221236
SGPP2	TOPBP1	liver	0.223173	0.000727	0.216498	0.001054	0.147335
SMPD1	ACER1	adipose_su	0.027492	0.479762	0.13667	0.000417	0.091432
SMPD1	ACER1	liver	-0.12792	0.054829	-0.26094	7.19E-05	-0.17691
SMPD1	ACER2	adipose_su	-0.29167	1.81E-14	-0.28846	3.59E-14	-0.19505
SMPD1	ACER2	liver	0.409513	1.50E-10	0.638243	2.96E-27	0.459115
SMPD1	CERS1	adipose_su	0.104234	0.007228	0.085525	0.027662	0.057456
SMPD1	CERS1	liver	0.037656	0.57333	-0.01339	0.841282	-0.01038
SMPD1	CERS6	adipose_su	-0.03852	0.32207	-0.03904	0.315485	-0.02555
SMPD1	CERS6	liver	0.579306	1.18E-21	0.719942	2.17E-37	0.521416
SMPD1	DEGS2	adipose_su	0.125738	0.001177	0.244534	1.75E-10	0.164851
SMPD1	DEGS2	liver	-0.05241	0.432965	-0.27936	2.03E-05	-0.18623
SMPD1	MAPK7	adipose_su	0.442138	4.19E-33	0.432809	1.20E-31	0.299759
SMPD1	MAPK7	liver	0.409096	1.57E-10	0.616424	4.82E-25	0.437522
SMPD1	SGMS1	adipose_su	0.291339	1.95E-14	0.294011	1.10E-14	0.198525
SMPD1	SGMS1	liver	-0.02193	0.742975	-0.0406	0.543723	-0.02376
SMPD1	SPHK1	adipose_su	0.111164	0.004159	0.127863	0.000968	0.086574
SMPD1	SPHK1	liver	0.106773	0.109415	0.33916	1.73E-07	0.229381
SMPD1	SPTLC3	adipose_su	0.176694	4.71E-06	0.230792	1.82E-09	0.157469

SMPD1	SPTLC3	liver	0.427111	1.95E-11	0.384188	2.31E-09	0.268201
SMPD2	ACER1	adipose_su	0.170368	1.03E-05	0.099747	0.010172	0.066397
SMPD2	ACER1	liver	-0.04239	0.526094	-0.24135	0.00025	-0.1626
SMPD2	ACER2	adipose_su	-0.14784	0.000133	-0.15553	5.78E-05	-0.10561
SMPD2	ACER2	liver	0.381909	2.92E-09	0.593164	7.21E-23	0.417502
SMPD2	B4GALT5	adipose_su	0.016538	0.670793	0.068622	0.077451	0.047076
SMPD2	B4GALT5	liver	0.530389	8.50E-18	0.574172	3.22E-21	0.418328
SMPD2	CBR3	adipose_su	0.159597	3.66E-05	0.162404	2.65E-05	0.11022
SMPD2	CBR3	liver	0.396431	6.34E-10	0.312454	1.65E-06	0.208496
SMPD2	CDR2	adipose_su	0.013389	0.730763	0.016801	0.665866	0.010749
SMPD2	CDR2	liver	0.388641	1.45E-09	0.325332	5.72E-07	0.223874
SMPD2	CERS1	adipose_su	0.191933	6.39E-07	0.175133	5.73E-06	0.117059
SMPD2	CERS1	liver	0.223506	0.000714	-0.04525	0.498554	-0.03508
SMPD2	CERS2	adipose_su	0.349197	1.90E-20	0.362678	4.85E-22	0.249188
SMPD2	CERS2	liver	0.347547	8.14E-08	0.471651	6.42E-14	0.327434
SMPD2	CERS3	adipose_su	0.187803	1.12E-06	0.171531	8.94E-06	0.115733
SMPD2	CERS3	liver	0.067383	0.313205	0.068245	0.30704	0.045742
SMPD2	CERS5	adipose_su	0.395107	3.38E-26	0.365231	2.37E-22	0.249548
SMPD2	CERS5	liver	0.75383	9.83E-43	0.67289	3.72E-31	0.497581
SMPD2	CERS6	adipose_su	0.007212	0.852952	0.005811	0.881278	0.004958
SMPD2	CERS6	liver	0.402671	3.21E-10	0.579391	1.16E-21	0.407276
SMPD2	DEGS1	adipose_su	0.169866	1.09E-05	0.092265	0.017487	0.061116
SMPD2	DEGS1	liver	0.613547	9.16E-25	0.684212	1.51E-32	0.497856
SMPD2	DEGS2	adipose_su	0.203354	1.28E-07	0.101224	0.009102	0.067413
SMPD2	DEGS2	liver	0.124655	0.06136	-0.12253	0.065959	-0.08382
SMPD2	HKDC1	adipose_su	0.181703	2.49E-06	0.221321	8.43E-09	0.149103
SMPD2	HKDC1	liver	0.242005	0.00024	0.453093	7.69E-13	0.308869
SMPD2	KCTD3	adipose_su	-0.02169	0.577158	0.009947	0.79823	0.005172
SMPD2	KCTD3	liver	-0.11042	0.097744	-0.09644	0.148438	-0.06269
SMPD2	MAPK7	adipose_su	0.364782	2.69E-22	0.377334	7.33E-24	0.258557
SMPD2	MAPK7	liver	0.655445	3.97E-29	0.697465	2.92E-34	0.511386
SMPD2	SEMA4F	adipose_su	0.343159	9.29E-20	0.382203	1.73E-24	0.260712
SMPD2	SEMA4F	liver	0.393805	8.40E-10	0.48548	9.13E-15	0.333137
SMPD2	SGMS1	adipose_su	0.24706	1.12E-10	0.274094	6.86E-13	0.184509
SMPD2	SGMS1	liver	-0.1225	0.066023	-0.16552	0.012713	-0.11296
SMPD2	SGMS2	adipose_su	0.271584	1.13E-12	0.333635	1.06E-18	0.226787
SMPD2	SGMS2	liver	0.246273	0.000184	0.477029	3.04E-14	0.346667
SMPD2	SGPL1	adipose_su	0.174009	6.59E-06	0.191643	6.65E-07	0.130921
SMPD2	SGPL1	liver	0.334915	2.51E-07	0.3181	1.04E-06	0.218053
SMPD2	SGPP2	adipose_su	0.085519	0.027673	-0.07127	0.066666	-0.04977
SMPD2	SGPP2	liver	0.446494	1.80E-12	0.175694	0.008115	0.112173
SMPD2	SMPD1	adipose_su	0.359866	1.06E-21	0.368179	1.03E-22	0.252605
SMPD2	SMPD1	liver	0.494155	2.56E-15	0.673755	2.92E-31	0.486411
SMPD2	SMSr	adipose_su	-0.04384	0.259635	-0.04489	0.248432	-0.02948

SMPD2	SMSr	liver	0.65233	8.85E-29	0.690801	2.18E-33	0.503953
SMPD2	SPHK1	adipose_su	0.195242	4.05E-07	0.21941	1.14E-08	0.147699
SMPD2	SPHK1	liver	0.577278	1.76E-21	0.475454	3.79E-14	0.34061
SMPD2	SPTLC3	adipose_su	0.237138	6.29E-10	0.219693	1.09E-08	0.150743
SMPD2	SPTLC3	liver	-0.05842	0.382046	0.086405	0.195605	0.073274
SMPD2	TOPBP1	adipose_su	0.128979	0.000873	0.164745	2.02E-05	0.112534
SMPD2	TOPBP1	liver	0.596646	3.50E-23	0.67307	3.54E-31	0.487276
SMPD2	UGCG	adipose_su	0.01031	0.791026	-0.00618	0.87376	-0.00415
SMPD2	UGCG	liver	0.213339	0.001253	0.303654	3.32E-06	0.206647
SMPD3	ACER1	adipose_su	0.14051	0.000284	0.142476	0.000233	0.095515
SMPD3	ACER1	liver	-0.02135	0.74954	0.029564	0.658437	0.015693
SMPD3	ACER2	adipose_su	-0.03257	0.402419	-0.04682	0.228629	-0.03062
SMPD3	ACER2	liver	-0.03156	0.636963	-0.03594	0.590909	-0.01738
SMPD3	B4GALT5	adipose_su	-0.22602	3.98E-09	-0.17775	4.13E-06	-0.12136
SMPD3	B4GALT5	liver	-0.05186	0.437845	-0.09826	0.140862	-0.05251
SMPD3	CBR3	adipose_su	0.223794	5.69E-09	0.255677	2.35E-11	0.172802
SMPD3	CBR3	liver	-0.07694	0.249356	-0.02899	0.664639	-0.02014
SMPD3	CDR2	adipose_su	0.136602	0.00042	0.164918	1.98E-05	0.110484
SMPD3	CDR2	liver	-0.15535	0.01946	-0.17623	0.007919	-0.11426
SMPD3	CERS1	adipose_su	0.193594	5.09E-07	0.200947	1.81E-07	0.137934
SMPD3	CERS1	liver	-0.13576	0.041439	-0.22938	0.00051	-0.15272
SMPD3	CERS2	adipose_su	0.09708	0.012389	0.072405	0.062425	0.047582
SMPD3	CERS2	liver	0.126649	0.057295	0.080221	0.229662	0.056637
SMPD3	CERS3	adipose_su	0.149643	0.00011	0.296509	6.37E-15	0.201105
SMPD3	CERS3	liver	-0.00298	0.964518	0.067717	0.31081	0.046293
SMPD3	CERS5	adipose_su	0.304065	1.20E-15	0.2973	5.36E-15	0.202754
SMPD3	CERS5	liver	-0.15786	0.017554	-0.13908	0.03667	-0.08842
SMPD3	CERS6	adipose_su	-0.1132	0.003516	-0.12525	0.00123	-0.08238
SMPD3	CERS6	liver	0.154965	0.019763	0.15574	0.019149	0.10588
SMPD3	DEGS1	adipose_su	-0.23819	5.26E-10	-0.39145	1.05E-25	-0.27119
SMPD3	DEGS1	liver	-0.06668	0.318257	-0.01527	0.819439	-0.00971
SMPD3	DEGS2	adipose_su	0.248702	8.36E-11	0.380498	2.88E-24	0.263464
SMPD3	DEGS2	liver	-0.11675	0.079881	-0.12445	0.061781	-0.07992
SMPD3	EDA2R	adipose_su	0.403555	2.33E-27	0.436235	3.55E-32	0.30001
SMPD3	EDA2R	liver	-0.05495	0.410992	0.026067	0.696714	0.016716
SMPD3	HKDC1	adipose_su	0.251494	5.05E-11	0.24112	3.18E-10	0.162235
SMPD3	HKDC1	liver	-0.04375	0.512895	0.069066	0.301248	0.046883
SMPD3	KCTD3	adipose_su	-0.0383	0.324845	0.008074	0.835609	0.006553
SMPD3	KCTD3	liver	0.194068	0.003398	0.156133	0.018844	0.105526
SMPD3	KDSR	adipose_su	0.057306	0.140486	0.046607	0.230743	0.031565
SMPD3	KDSR	liver	-0.11209	0.092751	-0.15562	0.019241	-0.09809
SMPD3	MAPK7	adipose_su	0.039636	0.308182	0.054919	0.157802	0.036691
SMPD3	MAPK7	liver	-0.08425	0.207005	-0.07046	0.291586	-0.03559
SMPD3	SEMA4F	adipose_su	0.312882	1.60E-16	0.359848	1.06E-21	0.243291

SMPD3	SEMA4F	liver	-0.04503	0.500565	0.053312	0.425112	0.033471
SMPD3	SGMS1	adipose_su	0.306646	6.68E-16	0.307289	5.77E-16	0.208664
SMPD3	SGMS1	liver	-0.15861	0.017015	-0.14829	0.025799	-0.10214
SMPD3	SGMS2	adipose_su	-0.10328	0.00778	-0.06573	0.090803	-0.04383
SMPD3	SGMS2	liver	-0.12093	0.069596	-0.14876	0.025323	-0.09129
SMPD3	SGPL1	adipose_su	0.061118	0.115898	0.022806	0.55775	0.017544
SMPD3	SGPL1	liver	0.098426	0.140199	0.071075	0.287371	0.050777
SMPD3	SGPP1	adipose_su	-0.10796	0.005392	-0.10473	0.006956	-0.07113
SMPD3	SGPP1	liver	0.066454	0.319941	0.080373	0.228773	0.060688
SMPD3	SGPP2	adipose_su	0.035697	0.358771	-0.08546	0.027778	-0.05861
SMPD3	SGPP2	liver	-0.03227	0.629409	-0.1206	0.07037	-0.08067
SMPD3	SMPD1	adipose_su	0.032535	0.402944	0.046908	0.227738	0.031041
SMPD3	SMPD1	liver	0.035398	0.596549	0.105916	0.112308	0.074022
SMPD3	SMPD2	adipose_su	0.159872	3.54E-05	0.154626	6.39E-05	0.10343
SMPD3	SMPD2	liver	-0.06351	0.341888	0.028488	0.670118	0.024149
SMPD3	SMSr	adipose_su	0.015155	0.696903	-0.01092	0.779033	-0.00839
SMPD3	SMSr	liver	-0.06717	0.314746	-0.07912	0.236135	-0.04869
SMPD3	SPHK1	adipose_su	-0.02673	0.492083	0.094164	0.015291	0.063804
SMPD3	SPHK1	liver	-0.15446	0.020176	-0.24001	0.000271	-0.16232
SMPD3	SPTLC3	adipose_su	0.366027	1.90E-22	0.385901	5.71E-25	0.26484
SMPD3	SPTLC3	liver	0.287651	1.12E-05	0.457613	4.26E-13	0.32236
SMPD3	ST3GAL5	adipose_su	0.345571	4.95E-20	0.376806	8.55E-24	0.255002
SMPD3	ST3GAL5	liver	0.022836	0.732776	0.116664	0.080102	0.079803
SMPD3	TOPBP1	adipose_su	0.076772	0.048157	0.090262	0.020099	0.062305
SMPD3	TOPBP1	liver	-0.02975	0.65641	-0.00185	0.977923	-0.00118
SMPD3	UGCG	adipose_su	-0.10198	0.008593	-0.0839	0.030762	-0.05698
SMPD3	UGCG	liver	-0.20622	0.00183	-0.26853	4.32E-05	-0.1775
SMPD3	ZDHHC2	adipose_su	0.118389	0.002263	0.123135	0.00149	0.082861
SMPD3	ZDHHC2	liver	0.117408	0.078182	0.110602	0.0972	0.076067
SMSr	ACER1	adipose_su	-0.03756	0.334171	0.111569	0.004023	0.076422
SMSr	ACER1	liver	-0.06335	0.343121	-0.20952	0.001538	-0.14631
SMSr	ACER2	adipose_su	0.530765	1.87E-49	0.516869	1.46E-46	0.359909
SMSr	ACER2	liver	0.637331	3.70E-27	0.742334	7.93E-41	0.54297
SMSr	B4GALT5	adipose_su	0.211625	3.77E-08	0.274303	6.58E-13	0.187521
SMSr	B4GALT5	liver	0.631658	1.44E-26	0.679626	5.61E-32	0.505408
SMSr	CBR3	adipose_su	-0.03912	0.314491	0.039997	0.303787	0.025677
SMSr	CBR3	liver	0.316791	1.16E-06	0.27386	2.99E-05	0.18529
SMSr	CERS1	adipose_su	-0.10462	0.007016	-0.14085	0.000275	-0.09356
SMSr	CERS1	liver	0.325737	5.53E-07	0.064176	0.336846	0.040983
SMSr	CERS6	adipose_su	0.526332	1.62E-48	0.51507	3.37E-46	0.36637
SMSr	CERS6	liver	0.637504	3.54E-27	0.715664	9.02E-37	0.527473
SMSr	DEGS2	adipose_su	-0.02933	0.45089	0.000454	0.990698	0.001969
SMSr	DEGS2	liver	0.088583	0.184537	-0.10577	0.112809	-0.07162
SMSr	MAPK7	adipose_su	0.025348	0.51469	0.034773	0.371347	0.02293

SMSr	MAPK7	liver	0.656747	2.83E-29	0.723401	6.73E-38	0.53412
SMSr	SGMS1	adipose_su	0.283723	9.67E-14	0.288071	3.90E-14	0.193754
SMSr	SGMS1	liver	0.063636	0.340938	0.062637	0.348582	0.042399
SMSr	SGMS2	adipose_su	0.118079	0.002324	0.101349	0.009017	0.06714
SMSr	SGMS2	liver	0.35421	4.40E-08	0.642497	1.05E-27	0.479135
SMSr	SGPL1	adipose_su	0.399355	8.90E-27	0.440408	7.87E-33	0.309488
SMSr	SGPL1	liver	0.203552	0.002103	0.219141	0.000911	0.155674
SMSr	SGPP2	adipose_su	-0.06955	0.073524	-0.04246	0.274916	-0.02879
SMSr	SGPP2	liver	0.204093	0.002044	0.181653	0.006172	0.119803
SMSr	SMPD1	adipose_su	0.016427	0.672866	0.032299	0.406362	0.02056
SMSr	SMPD1	liver	0.465871	1.41E-13	0.597456	2.95E-23	0.426706
SMSr	SPHK1	adipose_su	-0.10663	0.005991	-0.20712	7.39E-08	-0.14297
SMSr	SPHK1	liver	0.470054	8.00E-14	0.520942	4.02E-17	0.375929
SMSr	SPTLC3	adipose_su	-0.00798	0.837433	0.033843	0.384288	0.022984
SMSr	SPTLC3	liver	0.036444	0.585742	0.067298	0.313816	0.063284
SMSr	TOPBP1	adipose_su	0.501115	1.92E-43	0.510387	2.93E-45	0.360756
SMSr	TOPBP1	liver	0.67385	2.85E-31	0.770048	1.31E-45	0.573884
SPHK1	ACER2	adipose_su	-0.15474	6.31E-05	-0.26447	4.50E-12	-0.17632
SPHK1	ACER2	liver	0.278977	2.09E-05	0.526416	1.64E-17	0.367119
SPHK1	CERS1	adipose_su	0.277861	3.22E-13	0.409924	2.95E-28	0.279468
SPHK1	CERS1	liver	0.426283	2.16E-11	0.234772	0.000371	0.158781
SPHK1	SGMS1	adipose_su	0.071649	0.065217	0.178123	3.94E-06	0.121251
SPHK1	SGMS1	liver	-0.03703	0.579688	-0.10736	0.107478	-0.07422
SPHK2	ACER1	adipose_su	-0.05611	0.148962	0.008811	0.820858	0.006024
SPHK2	ACER1	liver	0.203585	0.002099	0.310544	1.93E-06	0.21176
SPHK2	ACER2	adipose_su	-0.16184	2.83E-05	-0.14038	0.000288	-0.09453
SPHK2	ACER2	liver	-0.27167	3.48E-05	-0.28028	1.90E-05	-0.18773
SPHK2	ACER3	adipose_su	0.201929	1.57E-07	0.199114	2.35E-07	0.133737
SPHK2	ACER3	liver	-0.03611	0.589144	-0.07457	0.264249	-0.05019
SPHK2	B4GALT5	adipose_su	-0.30023	2.81E-15	-0.2914	1.92E-14	-0.20024
SPHK2	B4GALT5	liver	-0.40473	2.56E-10	-0.3848	2.17E-09	-0.25652
SPHK2	CBR3	adipose_su	0.010766	0.782012	0.089384	0.021347	0.059384
SPHK2	CBR3	liver	0.144219	0.030204	0.189931	0.004162	0.125978
SPHK2	CDR2	adipose_su	-0.37172	3.74E-23	-0.38623	5.16E-25	-0.26369
SPHK2	CDR2	liver	-0.28089	1.82E-05	-0.28331	1.53E-05	-0.18918
SPHK2	CERK	adipose_su	0.092127	0.017657	0.137747	0.000375	0.094016
SPHK2	CERK	liver	-0.14292	0.031736	-0.07029	0.292762	-0.04134
SPHK2	CERS1	adipose_su	0.013704	0.724687	-0.09139	0.01859	-0.05965
SPHK2	CERS1	liver	-0.06754	0.312075	-0.01629	0.80761	-0.01259
SPHK2	CERS2	adipose_su	0.249654	7.05E-11	0.307669	5.29E-16	0.210109
SPHK2	CERS2	liver	0.165264	0.012853	0.188966	0.004361	0.128338
SPHK2	CERS3	adipose_su	-0.04227	0.277093	0.149275	0.000114	0.101051
SPHK2	CERS3	liver	0.00769	0.908475	0.055061	0.41006	0.037483
SPHK2	CERS4	adipose_su	0.192315	6.06E-07	0.252248	4.41E-11	0.173814

SPHK2	CERS4	liver	0.589583	1.50E-22	0.576963	1.87E-21	0.40944
SPHK2	CERS5	adipose_su	-0.1032	0.007828	-0.04589	0.238022	-0.0298
SPHK2	CERS5	liver	-0.2253	0.000644	-0.2197	0.000883	-0.14564
SPHK2	CERS6	adipose_su	0.409607	3.27E-28	0.425214	1.72E-30	0.28325
SPHK2	CERS6	liver	-0.09251	0.165758	-0.11664	0.080157	-0.07941
SPHK2	DEGS1	adipose_su	-0.07924	0.041387	-0.04568	0.240151	-0.03074
SPHK2	DEGS1	liver	-0.36949	1.02E-08	-0.36323	1.87E-08	-0.2433
SPHK2	DEGS2	adipose_su	-0.03749	0.335122	0.017723	0.648738	0.012627
SPHK2	DEGS2	liver	0.022184	0.740124	0.147875	0.026219	0.105447
SPHK2	EDA2R	adipose_su	-0.12067	0.001855	-0.13194	0.00066	-0.08351
SPHK2	EDA2R	liver	0.012788	0.848384	0.043954	0.510909	0.029105
SPHK2	HKDC1	adipose_su	0.082423	0.033847	0.169247	1.18E-05	0.115013
SPHK2	HKDC1	liver	-0.06765	0.311321	-0.0407	0.542691	-0.02655
SPHK2	KCTD3	adipose_su	0.083396	0.031791	0.132856	0.000604	0.091031
SPHK2	KCTD3	liver	0.462024	2.37E-13	0.454098	6.75E-13	0.319292
SPHK2	KDSR	adipose_su	0.076564	0.048769	0.149165	0.000116	0.102118
SPHK2	KDSR	liver	-0.4458	1.96E-12	-0.44003	4.05E-12	-0.29542
SPHK2	MAPK7	adipose_su	0.0941	0.015361	0.152571	8.01E-05	0.104774
SPHK2	MAPK7	liver	-0.14061	0.034629	-0.15822	0.017297	-0.10077
SPHK2	SEMA4F	adipose_su	0.079981	0.039512	0.146068	0.000161	0.095446
SPHK2	SEMA4F	liver	0.123898	0.062965	0.137103	0.039454	0.092586
SPHK2	SGMS1	adipose_su	-0.02671	0.492426	0.049747	0.20079	0.034089
SPHK2	SGMS1	liver	0.401053	3.84E-10	0.394736	7.60E-10	0.274533
SPHK2	SGMS2	adipose_su	-0.01422	0.714812	0.067579	0.082073	0.043996
SPHK2	SGMS2	liver	-0.40124	3.76E-10	-0.30895	2.19E-06	-0.20909
SPHK2	SGPL1	adipose_su	0.361224	7.26E-22	0.466405	4.08E-37	0.323081
SPHK2	SGPL1	liver	0.495128	2.22E-15	0.489648	4.98E-15	0.35233
SPHK2	SGPP1	adipose_su	0.044629	0.251154	0.088089	0.02331	0.05979
SPHK2	SGPP1	liver	-0.27233	3.32E-05	-0.31995	8.97E-07	-0.21459
SPHK2	SGPP2	adipose_su	-0.03341	0.390422	0.079466	0.040803	0.054244
SPHK2	SGPP2	liver	-0.13838	0.037641	-0.06971	0.296765	-0.04633
SPHK2	SMPD1	adipose_su	0.415533	4.60E-29	0.462796	1.69E-36	0.319207
SPHK2	SMPD1	liver	-0.01127	0.866227	-0.03738	0.576164	-0.02128
SPHK2	SMPD2	adipose_su	0.256068	2.19E-11	0.320375	2.73E-17	0.219086
SPHK2	SMPD2	liver	-0.16792	0.01146	-0.1501	0.02402	-0.10136
SPHK2	SMPD3	adipose_su	0.102597	0.0082	0.114481	0.003158	0.080035
SPHK2	SMPD3	liver	0.208506	0.001622	0.308344	2.30E-06	0.21176
SPHK2	SMSr	adipose_su	-0.00432	0.911673	0.030418	0.434249	0.021326
SPHK2	SMSr	liver	-0.26923	4.11E-05	-0.26061	7.34E-05	-0.17137
SPHK2	SPHK1	adipose_su	-0.31388	1.26E-16	-0.32919	3.20E-18	-0.22573
SPHK2	SPHK1	liver	-0.23627	0.00034	-0.37599	5.33E-09	-0.25974
SPHK2	SPTLC1	adipose_su	0.022228	0.567774	0.06915	0.07519	0.046867
SPHK2	SPTLC1	liver	-0.06419	0.336764	-0.07612	0.254411	-0.04216
SPHK2	SPTLC2	adipose_su	0.006411	0.869129	0.047463	0.222281	0.032107

SPHK2	SPTLC2	liver	-0.1415	0.033487	-0.15616	0.01882	-0.10041
SPHK2	SPTLC3	adipose_su	-0.02837	0.465848	0.010703	0.783255	0.00653
SPHK2	SPTLC3	liver	0.489787	4.88E-15	0.51649	8.23E-17	0.360393
SPHK2	ST3GAL5	adipose_su	0.027765	0.475414	0.095208	0.014189	0.061662
SPHK2	ST3GAL5	liver	0.282764	1.59E-05	0.343709	1.15E-07	0.244248
SPHK2	TOPBP1	adipose_su	0.094195	0.015257	0.158911	3.95E-05	0.110639
SPHK2	TOPBP1	liver	0.035117	0.599468	0.032971	0.621974	0.029066
SPHK2	TTC39A	adipose_su	0.128408	0.00092	0.183654	1.93E-06	0.124341
SPHK2	TTC39A	liver	-0.36266	1.98E-08	-0.38275	2.68E-09	-0.25215
SPHK2	UGCG	adipose_su	-0.43687	2.82E-32	-0.51621	1.99E-46	-0.35718
SPHK2	UGCG	liver	-0.37001	9.66E-09	-0.35148	5.67E-08	-0.23756
SPHK2	ZDHHC2	adipose_su	0.022595	0.561397	0.08319	0.032216	0.05434
SPHK2	ZDHHC2	liver	-0.08148	0.222402	-0.09675	0.147125	-0.06557
SPTLC1	ACER1	adipose_su	0.05635	0.14724	0.146436	0.000154	0.098139
SPTLC1	ACER1	liver	-0.10406	0.118766	-0.29724	5.44E-06	-0.20865
SPTLC1	ACER2	adipose_su	0.16138	2.98E-05	0.114313	0.003203	0.076299
SPTLC1	ACER2	liver	0.521246	3.83E-17	0.671283	5.79E-31	0.482989
SPTLC1	B4GALT5	adipose_su	0.305775	8.14E-16	0.36682	1.52E-22	0.252031
SPTLC1	B4GALT5	liver	0.679738	5.44E-32	0.683742	1.72E-32	0.503245
SPTLC1	CBR3	adipose_su	0.116559	0.002648	0.144902	0.000181	0.097989
SPTLC1	CBR3	liver	0.445612	2.01E-12	0.30109	4.05E-06	0.203815
SPTLC1	CDR2	adipose_su	0.326407	6.35E-18	0.324123	1.11E-17	0.221218
SPTLC1	CDR2	liver	0.581008	8.44E-22	0.460986	2.72E-13	0.324838
SPTLC1	CERK	adipose_su	0.312624	1.69E-16	0.297946	4.65E-15	0.203716
SPTLC1	CERK	liver	0.422883	3.22E-11	0.331967	3.25E-07	0.234651
SPTLC1	CERS1	adipose_su	0.076251	0.049701	0.079217	0.041441	0.051993
SPTLC1	CERS1	liver	0.29784	5.20E-06	0.046742	0.484444	0.030324
SPTLC1	CERS2	adipose_su	0.561473	2.42E-56	0.557547	2.02E-55	0.390193
SPTLC1	CERS2	liver	0.696826	3.55E-34	0.74178	9.74E-41	0.55237
SPTLC1	CERS3	adipose_su	0.054468	0.161247	0.186446	1.34E-06	0.125713
SPTLC1	CERS3	liver	0.153487	0.020982	0.257573	8.97E-05	0.16944
SPTLC1	CERS4	adipose_su	0.285146	7.20E-14	0.308691	4.19E-16	0.210022
SPTLC1	CERS4	liver	0.080462	0.22826	0.096433	0.148447	0.073196
SPTLC1	CERS5	adipose_su	0.49706	1.15E-42	0.468678	1.65E-37	0.325755
SPTLC1	CERS5	liver	0.644189	6.88E-28	0.553083	1.65E-19	0.399371
SPTLC1	CERS6	adipose_su	0.312423	1.78E-16	0.248615	8.49E-11	0.166323
SPTLC1	CERS6	liver	0.663287	5.05E-30	0.704535	3.26E-35	0.524208
SPTLC1	DEGS1	adipose_su	0.371058	4.53E-23	0.479462	2.07E-39	0.330189
SPTLC1	DEGS1	liver	0.713845	1.64E-36	0.727983	1.39E-38	0.538092
SPTLC1	DEGS2	adipose_su	0.079569	0.04054	0.11166	0.003993	0.075346
SPTLC1	DEGS2	liver	0.093478	0.161345	-0.11476	0.085197	-0.07127
SPTLC1	EDA2R	adipose_su	0.318692	4.08E-17	0.274495	6.33E-13	0.186587
SPTLC1	EDA2R	liver	0.281155	1.79E-05	0.235433	0.000357	0.161534
SPTLC1	HKDC1	adipose_su	0.102152	0.008483	0.164664	2.03E-05	0.110648

SPTLC1	HKDC1	liver	0.353455	4.72E-08	0.45448	6.42E-13	0.310796
SPTLC1	KCTD3	adipose_su	0.58811	6.27E-63	0.611877	2.41E-69	0.437593
SPTLC1	KCTD3	liver	0.351746	5.54E-08	0.370692	9.04E-09	0.267414
SPTLC1	KDSR	adipose_su	0.644312	4.93E-79	0.629591	1.71E-74	0.453063
SPTLC1	KDSR	liver	0.713298	1.96E-36	0.72053	1.78E-37	0.538682
SPTLC1	MAPK7	adipose_su	0.340596	1.80E-19	0.357483	2.04E-21	0.247219
SPTLC1	MAPK7	liver	0.689992	2.77E-33	0.698984	1.83E-34	0.512999
SPTLC1	SEMA4F	adipose_su	0.147777	0.000134	0.124977	0.001262	0.085622
SPTLC1	SEMA4F	liver	0.430038	1.38E-11	0.418463	5.40E-11	0.299508
SPTLC1	SGMS1	adipose_su	0.533054	6.06E-50	0.540616	1.38E-51	0.380578
SPTLC1	SGMS1	liver	0.19237	0.003695	0.171186	0.009929	0.113235
SPTLC1	SGMS2	adipose_su	0.426771	1.00E-30	0.449386	2.87E-34	0.3091
SPTLC1	SGMS2	liver	0.436879	5.98E-12	0.701099	9.54E-35	0.51174
SPTLC1	SGPL1	adipose_su	0.287158	4.72E-14	0.293341	1.27E-14	0.201032
SPTLC1	SGPL1	liver	0.463877	1.85E-13	0.419294	4.90E-11	0.29825
SPTLC1	SGPP1	adipose_su	0.636364	1.49E-76	0.648885	1.70E-80	0.465521
SPTLC1	SGPP1	liver	0.563943	2.25E-20	0.593715	6.43E-23	0.419312
SPTLC1	SGPP2	adipose_su	0.128739	0.000892	0.198435	2.59E-07	0.134653
SPTLC1	SGPP2	liver	0.395048	7.35E-10	0.343879	1.14E-07	0.235084
SPTLC1	SMPD1	adipose_su	0.296494	6.39E-15	0.308591	4.29E-16	0.211458
SPTLC1	SMPD1	liver	0.529453	9.94E-18	0.602872	9.37E-24	0.42061
SPTLC1	SMPD2	adipose_su	0.125992	0.00115	0.145796	0.000165	0.097356
SPTLC1	SMPD2	liver	0.655774	3.64E-29	0.586584	2.76E-22	0.423756
SPTLC1	SMPD3	adipose_su	0.036595	0.346806	0.017084	0.660587	0.01254
SPTLC1	SMPD3	liver	0.000412	0.995082	-0.06649	0.319691	-0.03976
SPTLC1	SMSr	adipose_su	0.554403	1.08E-54	0.5533	1.94E-54	0.389286
SPTLC1	SMSr	liver	0.753534	1.10E-42	0.776182	9.24E-47	0.582262
SPTLC1	SPHK1	adipose_su	0.134672	0.000507	0.125976	0.001152	0.085317
SPTLC1	SPHK1	liver	0.473954	4.67E-14	0.386203	1.87E-09	0.269617
SPTLC1	SPTLC2	adipose_su	0.214934	2.28E-08	0.22643	3.72E-09	0.151914
SPTLC1	SPTLC2	liver	0.809644	9.52E-54	0.76953	1.63E-45	0.578643
SPTLC1	SPTLC3	adipose_su	0.185644	1.49E-06	0.225558	4.29E-09	0.154616
SPTLC1	SPTLC3	liver	0.226657	0.000596	0.211255	0.001401	0.165506
SPTLC1	ST3GAL5	adipose_su	0.313529	1.37E-16	0.286209	5.76E-14	0.196625
SPTLC1	ST3GAL5	liver	0.196952	0.002943	0.161943	0.014804	0.11237
SPTLC1	TOPBP1	adipose_su	0.760985	2.52E-126	0.760426	4.92E-126	0.569953
SPTLC1	TOPBP1	liver	0.792546	5.13E-50	0.797651	4.30E-51	0.610895
SPTLC1	UGCG	adipose_su	0.118733	0.002196	0.176437	4.87E-06	0.120112
SPTLC1	UGCG	liver	0.289879	9.46E-06	0.330583	3.66E-07	0.225015
SPTLC1	ZDHHC2	adipose_su	0.386425	4.87E-25	0.367076	1.41E-22	0.253034
SPTLC1	ZDHHC2	liver	0.649826	1.67E-28	0.625029	6.79E-26	0.444838
SPTLC2	ACER1	adipose_su	0.000677	0.986128	-0.06934	0.07441	-0.04631
SPTLC2	ACER1	liver	-0.11692	0.079446	-0.30609	2.75E-06	-0.20712
SPTLC2	ACER2	adipose_su	0.529865	2.91E-49	0.517273	1.21E-46	0.357585

SPTLC2	ACER2	liver	0.528343	1.20E-17	0.733225	2.19E-39	0.534553
SPTLC2	B4GALT5	adipose_su	0.030153	0.438265	0.156286	5.31E-05	0.109673
SPTLC2	B4GALT5	liver	0.626298	5.06E-26	0.724325	4.90E-38	0.53884
SPTLC2	CBR3	adipose_su	-0.22583	4.10E-09	-0.20383	1.20E-07	-0.13887
SPTLC2	CBR3	liver	0.569523	7.86E-21	0.400052	4.28E-10	0.272763
SPTLC2	CDR2	adipose_su	-0.02387	0.539515	-0.04559	0.241089	-0.02957
SPTLC2	CDR2	liver	0.604125	7.16E-24	0.454582	6.34E-13	0.318741
SPTLC2	CERS1	adipose_su	-0.15394	6.89E-05	-0.23278	1.31E-09	-0.15875
SPTLC2	CERS1	liver	0.439185	4.50E-12	0.069318	0.299479	0.046962
SPTLC2	CERS2	adipose_su	0.197227	3.07E-07	0.235965	7.68E-10	0.157154
SPTLC2	CERS2	liver	0.60821	2.96E-24	0.65745	2.36E-29	0.468397
SPTLC2	CERS3	adipose_su	0.011773	0.762219	0.058886	0.129847	0.039922
SPTLC2	CERS3	liver	0.086587	0.194664	0.229432	0.000508	0.156735
SPTLC2	CERS5	adipose_su	-0.14487	0.000182	-0.15304	7.60E-05	-0.10281
SPTLC2	CERS5	liver	0.689913	2.83E-33	0.62881	2.81E-26	0.456283
SPTLC2	CERS6	adipose_su	0.47998	1.67E-39	0.516717	1.56E-46	0.3564
SPTLC2	CERS6	liver	0.682961	2.16E-32	0.741271	1.18E-40	0.544857
SPTLC2	DEGS1	adipose_su	0.090814	0.019347	0.107373	0.005649	0.071687
SPTLC2	DEGS1	liver	0.733915	1.71E-39	0.822039	1.07E-56	0.62529
SPTLC2	DEGS2	adipose_su	-0.0731	0.05993	-0.26459	4.40E-12	-0.17896
SPTLC2	DEGS2	liver	-0.05564	0.40514	-0.25498	0.000106	-0.16861
SPTLC2	EDA2R	adipose_su	-0.21737	1.57E-08	-0.25997	1.06E-11	-0.17581
SPTLC2	EDA2R	liver	0.296671	5.68E-06	0.264965	5.49E-05	0.178092
SPTLC2	HKDC1	adipose_su	-0.09056	0.019691	-0.0934	0.016139	-0.06335
SPTLC2	HKDC1	liver	0.397782	5.48E-10	0.556559	8.81E-20	0.386037
SPTLC2	KCTD3	adipose_su	0.217941	1.43E-08	0.240213	3.72E-10	0.161748
SPTLC2	KCTD3	liver	0.10078	0.130916	0.089446	0.180276	0.061986
SPTLC2	KDSR	adipose_su	0.110159	0.004515	0.146218	0.000158	0.097948
SPTLC2	KDSR	liver	0.602857	9.40E-24	0.686987	6.71E-33	0.505998
SPTLC2	MAPK7	adipose_su	-0.12921	0.000854	-0.12368	0.001419	-0.08275
SPTLC2	MAPK7	liver	0.797741	4.12E-51	0.784642	2.08E-48	0.592724
SPTLC2	SEMA4F	adipose_su	0.164145	2.16E-05	0.166554	1.63E-05	0.112949
SPTLC2	SEMA4F	liver	0.526304	1.67E-17	0.510236	2.21E-16	0.356106
SPTLC2	SGMS1	adipose_su	0.095343	0.014052	0.096265	0.013146	0.065782
SPTLC2	SGMS1	liver	0.046617	0.485618	-0.03412	0.609879	-0.01542
SPTLC2	SGMS2	adipose_su	-0.12897	0.000873	-0.15204	8.48E-05	-0.10279
SPTLC2	SGMS2	liver	0.292334	7.88E-06	0.589388	1.57E-22	0.422655
SPTLC2	SGPL1	adipose_su	0.267115	2.70E-12	0.297495	5.14E-15	0.202508
SPTLC2	SGPL1	liver	0.423428	3.02E-11	0.362743	1.96E-08	0.249833
SPTLC2	SGPP1	adipose_su	0.085394	0.027903	0.084228	0.030118	0.056613
SPTLC2	SGPP1	liver	0.507122	3.59E-16	0.636383	4.65E-27	0.448378
SPTLC2	SGPP2	adipose_su	-0.0934	0.016147	-0.20059	1.91E-07	-0.1358
SPTLC2	SGPP2	liver	0.294719	6.59E-06	0.258404	8.49E-05	0.173451
SPTLC2	SMPD1	adipose_su	-0.1998	2.13E-07	-0.2075	6.99E-08	-0.13821

SPTLC2	SMPD1	liver	0.582629	6.11E-22	0.752868	1.43E-42	0.541632
SPTLC2	SMPD2	adipose_su	-0.04565	0.240486	-0.01584	0.683983	-0.01212
SPTLC2	SMPD2	liver	0.648336	2.44E-28	0.698269	2.28E-34	0.498132
SPTLC2	SMPD3	adipose_su	-0.05344	0.169304	-0.03265	0.401219	-0.02223
SPTLC2	SMPD3	liver	-0.00808	0.903878	0.039517	0.554515	0.029577
SPTLC2	SMSr	adipose_su	0.495394	2.38E-42	0.525887	2.01E-48	0.365595
SPTLC2	SMSr	liver	0.68862	4.15E-33	0.751204	2.74E-42	0.559725
SPTLC2	SPHK1	adipose_su	-0.27414	6.79E-13	-0.36259	4.97E-22	-0.24967
SPTLC2	SPHK1	liver	0.465295	1.53E-13	0.459078	3.51E-13	0.328732
SPTLC2	SPTLC3	adipose_su	-0.05165	0.18407	-0.05214	0.179953	-0.03396
SPTLC2	SPTLC3	liver	0.204681	0.001983	0.246086	0.000187	0.177502
SPTLC2	ST3GAL5	adipose_su	-0.10534	0.006629	-0.07609	0.050189	-0.05344
SPTLC2	ST3GAL5	liver	0.179437	0.00684	0.244447	0.000207	0.166529
SPTLC2	TOPBP1	adipose_su	0.21584	1.98E-08	0.229633	2.21E-09	0.156302
SPTLC2	TOPBP1	liver	0.793905	2.67E-50	0.770252	1.20E-45	0.573097
SPTLC2	UGCG	adipose_su	-0.05529	0.155001	0.014346	0.712345	0.012422
SPTLC2	UGCG	liver	0.291143	8.61E-06	0.329946	3.86E-07	0.22647
SPTLC2	ZDHHC2	adipose_su	0.438966	1.33E-32	0.433136	1.07E-31	0.299536
SPTLC2	ZDHHC2	liver	0.710752	4.49E-36	0.719567	2.46E-37	0.513628
SPTLC3	ACER2	adipose_su	-0.12545	0.001209	-0.13528	0.000478	-0.09059
SPTLC3	ACER2	liver	0.023183	0.728869	0.112023	0.092951	0.081141
SPTLC3	CERS1	adipose_su	0.215057	2.24E-08	0.188471	1.02E-06	0.129126
SPTLC3	CERS1	liver	-0.20268	0.0022	-0.16262	0.014384	-0.11103
SPTLC3	SGMS1	adipose_su	0.541845	7.39E-52	0.587376	9.70E-63	0.417757
SPTLC3	SGMS1	liver	0.274094	2.94E-05	0.284451	1.41E-05	0.194218
SPTLC3	SPHK1	adipose_su	-0.0463	0.233818	0.042367	0.276012	0.029979
SPTLC3	SPHK1	liver	-0.24382	0.000215	-0.23885	0.000291	-0.15937
ST3GAL5	ACER1	adipose_su	-0.01178	0.762142	0.192636	5.80E-07	0.129928
ST3GAL5	ACER1	liver	-0.08144	0.22265	-0.06795	0.309116	-0.04944
ST3GAL5	ACER2	adipose_su	-0.0149	0.701846	-0.03394	0.382868	-0.02239
ST3GAL5	ACER2	liver	0.087217	0.191424	0.173573	0.008928	0.116657
ST3GAL5	B4GALT5	adipose_su	-0.03619	0.352185	0.054258	0.162876	0.03948
ST3GAL5	B4GALT5	liver	0.240941	0.000256	0.194185	0.003379	0.134474
ST3GAL5	CBR3	adipose_su	0.492651	7.84E-42	0.510262	3.10E-45	0.358013
ST3GAL5	CBR3	liver	0.044855	0.502278	0.078195	0.241678	0.050895
ST3GAL5	CDR2	adipose_su	0.256369	2.07E-11	0.295537	7.88E-15	0.202795
ST3GAL5	CDR2	liver	-0.05818	0.384028	-0.02002	0.764662	-0.01089
ST3GAL5	CERS1	adipose_su	0.124936	0.001266	0.166615	1.62E-05	0.111432
ST3GAL5	CERS1	liver	0.076799	0.25021	0.108736	0.103009	0.073196
ST3GAL5	CERS2	adipose_su	0.259629	1.13E-11	0.249393	7.38E-11	0.168656
ST3GAL5	CERS2	liver	0.315088	1.34E-06	0.273602	3.04E-05	0.185644
ST3GAL5	CERS3	adipose_su	-0.01969	0.612707	0.206177	8.49E-08	0.13844
ST3GAL5	CERS3	liver	0.137505	0.038874	0.15515	0.019615	0.104385
ST3GAL5	CERS5	adipose_su	0.469857	1.03E-37	0.455957	2.39E-35	0.321048

ST3GAL5	CERS5	liver	0.058361	0.382537	0.113937	0.087467	0.079371
ST3GAL5	CERS6	adipose_su	-0.25265	4.09E-11	-0.2841	8.94E-14	-0.18945
ST3GAL5	CERS6	liver	0.01785	0.789565	0.050891	0.446472	0.034454
ST3GAL5	DEGS1	adipose_su	-0.10075	0.009432	-0.11001	0.004569	-0.07255
ST3GAL5	DEGS1	liver	0.242758	0.000229	0.160932	0.015447	0.112763
ST3GAL5	DEGS2	adipose_su	0.085572	0.027576	0.350656	1.29E-20	0.239441
ST3GAL5	DEGS2	liver	-0.08981	0.178481	-0.08416	0.20749	-0.05585
ST3GAL5	EDA2R	adipose_su	0.457802	1.18E-35	0.479553	1.99E-39	0.332317
ST3GAL5	EDA2R	liver	-0.01421	0.831731	0.011754	0.860505	0.010423
ST3GAL5	HKDC1	adipose_su	0.180446	2.93E-06	0.219026	1.21E-08	0.146697
ST3GAL5	HKDC1	liver	-0.05866	0.3801	0.004085	0.951308	0.003068
ST3GAL5	KCTD3	adipose_su	0.055938	0.150225	0.09918	0.010611	0.070885
ST3GAL5	KCTD3	liver	0.104554	0.117028	0.099556	0.13568	0.06352
ST3GAL5	KDSR	adipose_su	0.385309	6.83E-25	0.360789	8.19E-22	0.248614
ST3GAL5	KDSR	liver	0.078971	0.237023	0.071243	0.286226	0.047276
ST3GAL5	MAPK7	adipose_su	0.269614	1.66E-12	0.323782	1.20E-17	0.222581
ST3GAL5	MAPK7	liver	0.194792	0.003278	0.275064	2.75E-05	0.19355
ST3GAL5	SEMA4F	adipose_su	0.176789	4.66E-06	0.240374	3.62E-10	0.162272
ST3GAL5	SEMA4F	liver	0.066882	0.316829	0.121238	0.068878	0.080905
ST3GAL5	SGMS1	adipose_su	0.533076	6.00E-50	0.535987	1.41E-50	0.375725
ST3GAL5	SGMS1	liver	0.111701	0.093901	0.12347	0.063887	0.081613
ST3GAL5	SGMS2	adipose_su	0.248343	8.91E-11	0.31899	3.80E-17	0.218224
ST3GAL5	SGMS2	liver	-0.14519	0.029101	0.020419	0.760145	0.02002
ST3GAL5	SGPL1	adipose_su	0.044673	0.250692	0.057546	0.13883	0.037739
ST3GAL5	SGPL1	liver	0.264737	5.58E-05	0.226671	0.000596	0.153432
ST3GAL5	SGPP1	adipose_su	0.29277	1.43E-14	0.286422	5.51E-14	0.196179
ST3GAL5	SGPP1	liver	0.385792	1.95E-09	0.232272	0.00043	0.155162
ST3GAL5	SGPP2	adipose_su	-0.03845	0.322917	-0.01162	0.765139	-0.0065
ST3GAL5	SGPP2	liver	0.019969	0.765273	0.02732	0.682896	0.016716
ST3GAL5	SMPD1	adipose_su	0.388536	2.57E-25	0.364288	3.09E-22	0.245875
ST3GAL5	SMPD1	liver	0.367316	1.26E-08	0.287055	1.16E-05	0.194612
ST3GAL5	SMPD2	adipose_su	0.278679	2.72E-13	0.306056	7.64E-16	0.207552
ST3GAL5	SMPD2	liver	0.171663	0.009722	0.160826	0.015516	0.107689
ST3GAL5	SMSr	adipose_su	0.16924	1.18E-05	0.168942	1.22E-05	0.113131
ST3GAL5	SMSr	liver	0.147849	0.026246	0.161449	0.015115	0.113117
ST3GAL5	SPHK1	adipose_su	0.139205	0.000324	0.260211	1.01E-11	0.180266
ST3GAL5	SPHK1	liver	-0.05805	0.385045	-0.0331	0.620593	-0.02081
ST3GAL5	SPTLC3	adipose_su	0.265509	3.69E-12	0.358698	1.46E-21	0.247406
ST3GAL5	SPTLC3	liver	0.246212	0.000185	0.283819	1.47E-05	0.197129
ST3GAL5	TOPBP1	adipose_su	0.354658	4.39E-21	0.358616	1.49E-21	0.244677
ST3GAL5	TOPBP1	liver	0.116259	0.081163	0.210898	0.001428	0.145369
ST3GAL5	UGCG	adipose_su	-0.02107	0.588113	0.090123	0.020292	0.06292
ST3GAL5	UGCG	liver	-0.04762	0.476227	0.021746	0.745072	0.017581
TOPBP1	ACER1	adipose_su	0.069617	0.073236	0.201967	1.57E-07	0.135118

TOPBP1	ACER1	liver	-0.04065	0.543184	-0.1941	0.003393	-0.13443
TOPBP1	ACER2	adipose_su	0.075691	0.051408	0.058685	0.131165	0.039667
TOPBP1	ACER2	liver	0.469752	8.34E-14	0.677321	1.08E-31	0.482557
TOPBP1	CERS1	adipose_su	0.001427	0.970735	0.042735	0.271861	0.029893
TOPBP1	CERS1	liver	0.241465	0.000248	0.006561	0.921865	0.003382
TOPBP1	CERS6	adipose_su	0.218023	1.41E-08	0.214513	2.43E-08	0.143962
TOPBP1	CERS6	liver	0.718807	3.18E-37	0.742182	8.39E-41	0.550521
TOPBP1	DEGS2	adipose_su	0.109365	0.004815	0.174012	6.59E-06	0.118358
TOPBP1	DEGS2	liver	0.074355	0.265644	-0.10577	0.112793	-0.0673
TOPBP1	MAPK7	adipose_su	0.358165	1.69E-21	0.390827	1.27E-25	0.269999
TOPBP1	MAPK7	liver	0.675573	1.76E-31	0.678442	7.85E-32	0.493609
TOPBP1	SGMS1	adipose_su	0.480598	1.29E-39	0.498113	7.25E-43	0.350508
TOPBP1	SGMS1	liver	0.203835	0.002072	0.127894	0.05487	0.088968
TOPBP1	SGMS2	adipose_su	0.390194	1.55E-25	0.446813	7.49E-34	0.311392
TOPBP1	SGMS2	liver	0.226808	0.000591	0.519823	4.82E-17	0.379626
TOPBP1	SGPL1	adipose_su	0.375212	1.36E-23	0.399848	7.61E-27	0.279504
TOPBP1	SGPL1	liver	0.53034	8.57E-18	0.46098	2.73E-13	0.328889
TOPBP1	SMPD1	adipose_su	0.348329	2.39E-20	0.350717	1.27E-20	0.238785
TOPBP1	SMPD1	liver	0.483405	1.23E-14	0.635956	5.15E-27	0.442124
TOPBP1	SPHK1	adipose_su	0.025628	0.510049	0.065468	0.092112	0.04297
TOPBP1	SPHK1	liver	0.316069	1.23E-06	0.361795	2.15E-08	0.254789
TOPBP1	SPTLC3	adipose_su	0.192746	5.72E-07	0.232764	1.31E-09	0.161766
TOPBP1	SPTLC3	liver	0.272568	3.27E-05	0.274026	2.96E-05	0.206057
TTC39A	ACER1	adipose_su	0.4469	7.25E-34	-0.05744	0.139581	-0.03941
TTC39A	ACER1	liver	-0.11492	0.084739	-0.35961	2.65E-08	-0.2422
TTC39A	ACER2	adipose_su	0.036091	0.353489	0.115402	0.002922	0.076928
TTC39A	ACER2	liver	0.225352	0.000643	0.593474	6.76E-23	0.414749
TTC39A	B4GALT5	adipose_su	0.009331	0.810484	0.002227	0.954368	0.00118
TTC39A	B4GALT5	liver	0.568017	1.05E-20	0.759974	8.51E-44	0.565978
TTC39A	CBR3	adipose_su	-0.22	1.04E-08	-0.24386	1.97E-10	-0.16505
TTC39A	CBR3	liver	0.173081	0.009127	0.230659	0.000473	0.155477
TTC39A	CDR2	adipose_su	-0.23213	1.46E-09	-0.3787	4.90E-24	-0.25742
TTC39A	CDR2	liver	0.320418	8.63E-07	0.417309	6.17E-11	0.294907
TTC39A	CERK	adipose_su	0.031878	0.412517	0.040116	0.302352	0.025245
TTC39A	CERK	liver	0.38686	1.75E-09	0.259675	7.81E-05	0.17412
TTC39A	CERS1	adipose_su	-0.08432	0.029932	-0.11395	0.003304	-0.07848
TTC39A	CERS1	liver	0.131835	0.047751	-0.00999	0.881228	-0.00739
TTC39A	CERS2	adipose_su	-0.02893	0.457018	0.001332	0.972696	0.000661
TTC39A	CERS2	liver	0.252642	0.000123	0.453272	7.52E-13	0.310678
TTC39A	CERS3	adipose_su	0.463866	1.11E-36	0.001182	0.975758	-9.11E-06
TTC39A	CERS3	liver	0.098754	0.138878	0.112114	0.092685	0.077758
TTC39A	CERS4	adipose_su	-0.06278	0.106314	-0.09542	0.013974	-0.06282
TTC39A	CERS4	liver	-0.32549	5.64E-07	-0.23495	0.000367	-0.15615
TTC39A	CERS5	adipose_su	-0.16593	1.75E-05	-0.31551	8.64E-17	-0.21607

TTC39A	CERS5	liver	0.591125	1.10E-22	0.630851	1.74E-26	0.448968
TTC39A	CERS6	adipose_su	0.239675	4.08E-10	0.322568	1.61E-17	0.214679
TTC39A	CERS6	liver	0.271732	3.46E-05	0.55677	8.47E-20	0.386352
TTC39A	DEGS1	adipose_su	0.428275	5.93E-31	0.194268	4.64E-07	0.130985
TTC39A	DEGS1	liver	0.555872	9.98E-20	0.809686	9.32E-54	0.604444
TTC39A	DEGS2	adipose_su	0.388982	2.24E-25	-0.14833	0.000126	-0.10095
TTC39A	DEGS2	liver	0.207543	0.001707	-0.16987	0.010524	-0.11622
TTC39A	EDA2R	adipose_su	-0.1137	0.003371	-0.21285	3.13E-08	-0.14404
TTC39A	EDA2R	liver	0.125079	0.060476	0.211762	0.001364	0.143481
TTC39A	HKDC1	adipose_su	-0.02466	0.526189	-0.05657	0.14565	-0.03792
TTC39A	HKDC1	liver	0.213068	0.001271	0.402733	3.19E-10	0.27528
TTC39A	KCTD3	adipose_su	-0.02404	0.536563	-0.05163	0.184275	-0.03514
TTC39A	KCTD3	liver	-0.21009	0.001491	-0.15269	0.021664	-0.09707
TTC39A	KDSR	adipose_su	-0.02618	0.50102	-0.06404	0.099462	-0.0429
TTC39A	KDSR	liver	0.662319	6.54E-30	0.728867	1.02E-38	0.545015
TTC39A	MAPK7	adipose_su	-0.11064	0.004342	-0.24717	1.10E-10	-0.16758
TTC39A	MAPK7	liver	0.466414	1.31E-13	0.715193	1.05E-36	0.525388
TTC39A	SEMA4F	adipose_su	0.093967	0.015507	0.1599	3.53E-05	0.105877
TTC39A	SEMA4F	liver	0.248605	0.000159	0.280348	1.89E-05	0.189577
TTC39A	SGMS1	adipose_su	-0.1011	0.009187	-0.2	2.07E-07	-0.13439
TTC39A	SGMS1	liver	-0.12749	0.055639	-0.25376	0.000115	-0.17023
TTC39A	SGMS2	adipose_su	0.048968	0.207946	0.004236	0.913318	0.001937
TTC39A	SGMS2	liver	0.336565	2.18E-07	0.622187	1.31E-25	0.442714
TTC39A	SGPL1	adipose_su	0.326377	6.40E-18	0.342107	1.22E-19	0.226823
TTC39A	SGPL1	liver	0.153093	0.021317	0.181537	0.006205	0.128614
TTC39A	SGPP1	adipose_su	0.007473	0.847701	-0.01583	0.684084	-0.01023
TTC39A	SGPP1	liver	0.464422	1.72E-13	0.664452	3.70E-30	0.474336
TTC39A	SGPP2	adipose_su	0.404976	1.48E-27	0.083922	0.030725	0.056814
TTC39A	SGPP2	liver	0.345693	9.64E-08	0.344479	1.08E-07	0.233235
TTC39A	SMPD1	adipose_su	0.024768	0.524365	0.088232	0.023086	0.059042
TTC39A	SMPD1	liver	0.258235	8.59E-05	0.602775	9.57E-24	0.42647
TTC39A	SMPD2	adipose_su	0.129107	0.000862	0.087676	0.023968	0.058906
TTC39A	SMPD2	liver	0.649955	1.62E-28	0.70697	1.51E-35	0.522281
TTC39A	SMPD3	adipose_su	-0.07865	0.042933	-0.18407	1.83E-06	-0.126
TTC39A	SMPD3	liver	-0.15275	0.021614	-0.08178	0.220713	-0.04279
TTC39A	SMSr	adipose_su	-0.05095	0.190131	0.068403	0.078403	0.045231
TTC39A	SMSr	liver	0.527275	1.43E-17	0.629068	2.65E-26	0.451957
TTC39A	SPHK1	adipose_su	-0.07864	0.042963	-0.30745	5.56E-16	-0.2074
TTC39A	SPHK1	liver	0.339077	1.74E-07	0.546329	5.51E-19	0.373412
TTC39A	SPTLC1	adipose_su	-0.01875	0.629778	-0.0386	0.320998	-0.02478
TTC39A	SPTLC1	liver	0.507126	3.59E-16	0.632861	1.08E-26	0.44885
TTC39A	SPTLC2	adipose_su	0.036137	0.352874	0.129604	0.000823	0.086861
TTC39A	SPTLC2	liver	0.456486	4.94E-13	0.68726	6.19E-33	0.497896
TTC39A	SPTLC3	adipose_su	0.200787	1.85E-07	-0.02821	0.468365	-0.01971

TTC39A	SPTLC3	liver	-0.21781	0.000981	-0.01259	0.850643	0.003265
TTC39A	ST3GAL5	adipose_su	-0.24932	7.48E-11	-0.29543	8.06E-15	-0.20226
TTC39A	ST3GAL5	liver	0.048784	0.46554	0.07271	0.276399	0.053255
TTC39A	TOPBP1	adipose_su	-0.07646	0.049076	-0.07967	0.040296	-0.05232
TTC39A	TOPBP1	liver	0.404725	2.56E-10	0.576799	1.93E-21	0.406726
TTC39A	UGCG	adipose_su	0.006932	0.858608	-0.05709	0.141996	-0.037
TTC39A	UGCG	liver	0.148768	0.025317	0.327451	4.78E-07	0.223009
TTC39A	ZDHHC2	adipose_su	0.006629	0.864725	0.064555	0.096753	0.043458
TTC39A	ZDHHC2	liver	0.294393	6.75E-06	0.524168	2.38E-17	0.364877
UGCG	ACER1	adipose_su	0.025169	0.517665	0.083683	0.031204	0.056276
UGCG	ACER1	liver	-0.04637	0.487901	-0.14578	0.028445	-0.09797
UGCG	ACER2	adipose_su	0.224852	4.80E-09	0.342076	1.23E-19	0.231366
UGCG	ACER2	liver	0.595416	4.52E-23	0.444327	2.36E-12	0.309538
UGCG	B4GALT5	adipose_su	0.714606	1.13E-104	0.719327	1.12E-106	0.531043
UGCG	B4GALT5	liver	0.38047	3.38E-09	0.448406	1.41E-12	0.311465
UGCG	CBR3	adipose_su	-0.16198	2.78E-05	-0.16036	3.35E-05	-0.10705
UGCG	CBR3	liver	0.187573	0.004664	0.195347	0.003189	0.136087
UGCG	CERS1	adipose_su	0.030683	0.430254	0.123758	0.001409	0.082172
UGCG	CERS1	liver	0.346838	8.69E-08	0.213282	0.001256	0.143009
UGCG	CERS3	adipose_su	0.017205	0.658337	-0.10003	0.009961	-0.06811
UGCG	CERS3	liver	0.027457	0.681396	0.201876	0.002292	0.136716
UGCG	CERS6	adipose_su	-0.19583	3.73E-07	-0.2995	3.30E-15	-0.19197
UGCG	CERS6	liver	0.243752	0.000216	0.258465	8.46E-05	0.178014
UGCG	DEGS2	adipose_su	-0.03446	0.375639	-0.0486	0.211393	-0.03455
UGCG	DEGS2	liver	0.047835	0.474275	0.138307	0.037739	0.091563
UGCG	HKDC1	adipose_su	-0.05407	0.164321	-0.03768	0.332625	-0.02407
UGCG	HKDC1	liver	0.345295	9.99E-08	0.365274	1.54E-08	0.253294
UGCG	MAPK7	adipose_su	0.063645	0.101558	0.070147	0.071074	0.045007
UGCG	MAPK7	liver	0.35055	6.18E-08	0.367967	1.18E-08	0.250737
UGCG	SGMS1	adipose_su	0.030963	0.426064	0.139595	0.000312	0.097018
UGCG	SGMS1	liver	0.087066	0.192194	0.192175	0.003731	0.134002
UGCG	SGMS2	adipose_su	0.128642	0.0009	0.131523	0.000686	0.088798
UGCG	SGMS2	liver	0.390363	1.21E-09	0.409676	1.47E-10	0.282203
UGCG	SGPL1	adipose_su	-0.21469	2.37E-08	-0.22756	3.10E-09	-0.15477
UGCG	SGPL1	liver	-0.25095	0.000137	-0.09977	0.134844	-0.06124
UGCG	SGPP2	adipose_su	-0.02605	0.503115	-0.12326	0.001473	-0.08127
UGCG	SGPP2	liver	0.040523	0.54447	0.039711	0.55257	0.026785
UGCG	SMPD1	adipose_su	-0.1272	0.001029	-0.13378	0.000553	-0.08874
UGCG	SMPD1	liver	0.141499	0.033495	0.231619	0.000447	0.155123
UGCG	SMSr	adipose_su	0.119791	0.002003	0.163077	2.45E-05	0.110083
UGCG	SMSr	liver	0.545778	6.07E-19	0.512769	1.49E-16	0.361691
UGCG	SPHK1	adipose_su	0.435033	5.46E-32	0.414333	6.87E-29	0.283127
UGCG	SPHK1	liver	0.414238	8.78E-11	0.500596	9.76E-16	0.350718
UGCG	SPTLC3	adipose_su	0.016455	0.672354	0.096397	0.01302	0.066534

UGCG	SPTLC3	liver	-0.17581	0.008071	-0.12272	0.06554	-0.0822
UGCG	TOPBP1	adipose_su	-0.07389	0.057213	0.045426	0.242787	0.030038
UGCG	TOPBP1	liver	0.216029	0.001082	0.290637	8.94E-06	0.200826
ZDHHC2	ACER1	adipose_su	-0.07462	0.054791	-0.06871	0.077079	-0.04643
ZDHHC2	ACER1	liver	-0.17167	0.00972	-0.25671	9.49E-05	-0.17849
ZDHHC2	ACER2	adipose_su	0.287658	4.25E-14	0.280159	2.02E-13	0.188341
ZDHHC2	ACER2	liver	0.449096	1.29E-12	0.574781	2.86E-21	0.396185
ZDHHC2	B4GALT5	adipose_su	-0.01328	0.732952	0.110058	0.004552	0.077424
ZDHHC2	B4GALT5	liver	0.520101	4.61E-17	0.556898	8.28E-20	0.399056
ZDHHC2	CBR3	adipose_su	0.020587	0.596695	0.031149	0.423295	0.019248
ZDHHC2	CBR3	liver	0.28986	9.47E-06	0.239556	0.000279	0.159725
ZDHHC2	CDR2	adipose_su	0.102749	0.008105	0.099125	0.010654	0.066725
ZDHHC2	CDR2	liver	0.370399	9.30E-09	0.340625	1.52E-07	0.236146
ZDHHC2	CERS1	adipose_su	0.039537	0.309394	0.011053	0.776359	0.007605
ZDHHC2	CERS1	liver	0.245533	0.000193	0.065594	0.326256	0.044523
ZDHHC2	CERS2	adipose_su	0.337762	3.73E-19	0.323992	1.14E-17	0.219696
ZDHHC2	CERS2	liver	0.677423	1.05E-31	0.641079	1.48E-27	0.460374
ZDHHC2	CERS3	adipose_su	-0.05952	0.125773	0.204045	1.16E-07	0.137597
ZDHHC2	CERS3	liver	0.063838	0.339402	0.132574	0.046506	0.086883
ZDHHC2	CERS5	adipose_su	0.111521	0.004039	0.107557	0.005566	0.072426
ZDHHC2	CERS5	liver	0.449459	1.23E-12	0.450056	1.14E-12	0.314533
ZDHHC2	CERS6	adipose_su	0.345713	4.77E-20	0.311637	2.13E-16	0.209494
ZDHHC2	CERS6	liver	0.634379	7.52E-27	0.65897	1.58E-29	0.467925
ZDHHC2	DEGS1	adipose_su	0.095034	0.014368	0.149432	0.000112	0.101028
ZDHHC2	DEGS1	liver	0.615405	6.05E-25	0.630869	1.73E-26	0.450816
ZDHHC2	DEGS2	adipose_su	-0.10238	0.00834	-0.12745	0.001006	-0.08294
ZDHHC2	DEGS2	liver	-0.0302	0.65151	-0.22374	0.000704	-0.1565
ZDHHC2	EDA2R	adipose_su	0.139135	0.000327	0.105713	0.00644	0.071282
ZDHHC2	EDA2R	liver	0.20989	0.001507	0.175407	0.008221	0.116028
ZDHHC2	HKDC1	adipose_su	0.047871	0.218323	0.05578	0.151379	0.037903
ZDHHC2	HKDC1	liver	0.335932	2.30E-07	0.389192	1.37E-09	0.269853
ZDHHC2	KCTD3	adipose_su	0.218066	1.41E-08	0.247141	1.10E-10	0.170629
ZDHHC2	KCTD3	liver	0.198952	0.002661	0.153405	0.021051	0.103363
ZDHHC2	KDSR	adipose_su	0.303659	1.31E-15	0.276425	4.30E-13	0.186938
ZDHHC2	KDSR	liver	0.48043	1.88E-14	0.580727	8.92E-22	0.427414
ZDHHC2	MAPK7	adipose_su	-0.0801	0.039205	-0.04353	0.263038	-0.0305
ZDHHC2	MAPK7	liver	0.551508	2.20E-19	0.594043	6.01E-23	0.423913
ZDHHC2	SEMA4F	adipose_su	0.48365	3.62E-40	0.410384	2.54E-28	0.28273
ZDHHC2	SEMA4F	liver	0.436238	6.47E-12	0.455882	5.35E-13	0.319135
ZDHHC2	SGMS1	adipose_su	0.481466	9.00E-40	0.460939	3.49E-36	0.319216
ZDHHC2	SGMS1	liver	0.066386	0.320439	0.046109	0.490385	0.029892
ZDHHC2	SGMS2	adipose_su	0.172331	8.11E-06	0.179846	3.16E-06	0.122227
ZDHHC2	SGMS2	liver	0.132304	0.046958	0.496881	1.71E-15	0.363265
ZDHHC2	SGPL1	adipose_su	0.272878	8.73E-13	0.269522	1.69E-12	0.182887

ZDHHC2	SGPL1	liver	0.443567	2.60E-12	0.39877	4.92E-10	0.281613
ZDHHC2	SGPP1	adipose_su	0.392861	6.79E-26	0.393094	6.32E-26	0.268873
ZDHHC2	SGPP1	liver	0.5978	2.74E-23	0.627561	3.77E-26	0.448535
ZDHHC2	SGPP2	adipose_su	-0.179	3.52E-06	-0.21077	4.29E-08	-0.1433
ZDHHC2	SGPP2	liver	0.05053	0.449703	0.050352	0.451308	0.03296
ZDHHC2	SMPD1	adipose_su	-0.03216	0.408409	-0.05067	0.192582	-0.03583
ZDHHC2	SMPD1	liver	0.622738	1.15E-25	0.653897	5.92E-29	0.4647
ZDHHC2	SMPD2	adipose_su	0.165187	1.91E-05	0.201196	1.75E-07	0.134612
ZDHHC2	SMPD2	liver	0.493603	2.78E-15	0.564035	2.21E-20	0.39296
ZDHHC2	SMSr	adipose_su	0.464784	7.75E-37	0.459004	7.39E-36	0.315922
ZDHHC2	SMSr	liver	0.614113	8.07E-25	0.654437	5.15E-29	0.472802
ZDHHC2	SPHK1	adipose_su	-0.16724	1.50E-05	-0.16185	2.83E-05	-0.11165
ZDHHC2	SPHK1	liver	0.184067	0.005511	0.258161	8.63E-05	0.185487
ZDHHC2	SPTLC3	adipose_su	0.187224	1.21E-06	0.250349	6.22E-11	0.169353
ZDHHC2	SPTLC3	liver	0.282968	1.57E-05	0.23613	0.000342	0.1635
ZDHHC2	ST3GAL5	adipose_su	0.187542	1.16E-06	0.204233	1.13E-07	0.137897
ZDHHC2	ST3GAL5	liver	0.356277	3.63E-08	0.28862	1.04E-05	0.193668
ZDHHC2	TOPBP1	adipose_su	0.302817	1.58E-15	0.30369	1.30E-15	0.207808
ZDHHC2	TOPBP1	liver	0.62173	1.45E-25	0.660731	9.96E-30	0.468397
ZDHHC2	UGCG	adipose_su	-0.00949	0.807322	0.064914	0.094905	0.045627
ZDHHC2	UGCG	liver	0.171469	0.009806	0.230332	0.000482	0.156775

Suppl. Table 4. Candidate Genes to Interact with SMSr

Gene	R(Human)	P(Human)	R(Mouse)	P(Mouse)
SPTLC2	0.75	4.2e-33	0.979	$2X10^{-5}$
CERS6	0.72	3.5e-27	0.879	0.003
CERS2	0.54	2.6e-13	-0.75	0.031
SGPP1	0.65	5.2e-23	-0.81	0.014
B4GALT5	0.68	$1X10^{-26}$	0.88	0.003
ST3GAL5	0.16	2.6e-02	-0.79	0.019

Suppl. Fig.1. Li et al

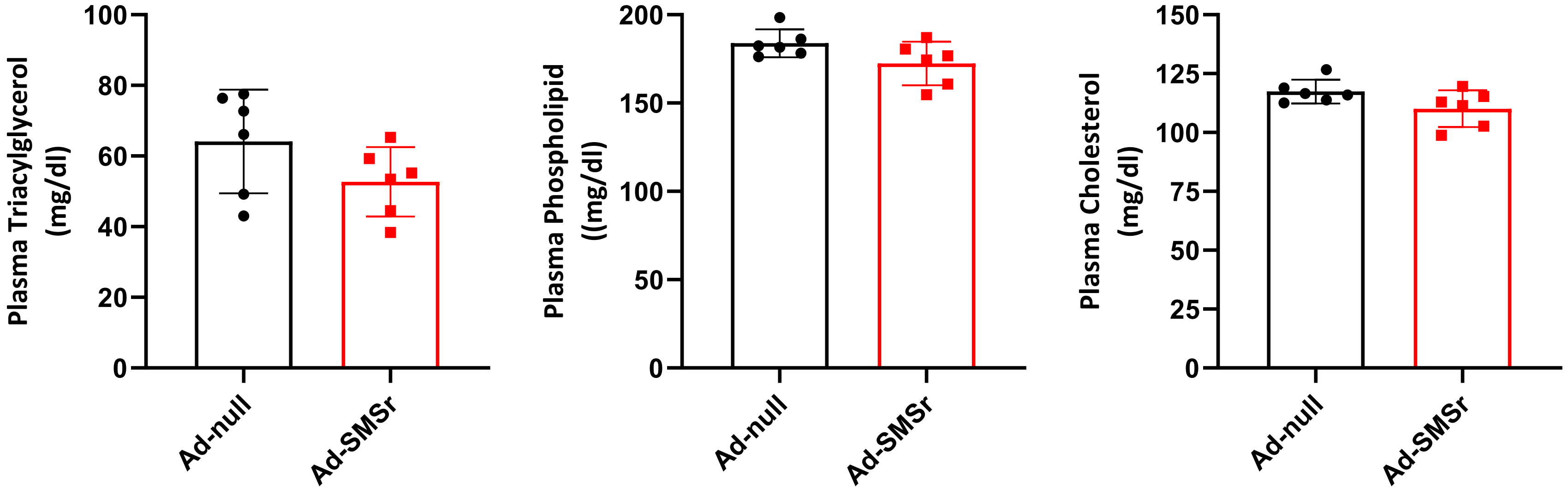

Fig. S1. Plasma lipid measurements. AdV-SMSr was injected into wild type mice. At day 4, At day one of the injection, plasma triglyceride (TAG), phospholipid (PL), and cholesterol (Chol) were measured. Values are mean ± SD, n=6.

Suppl. Fig.2. Li et al

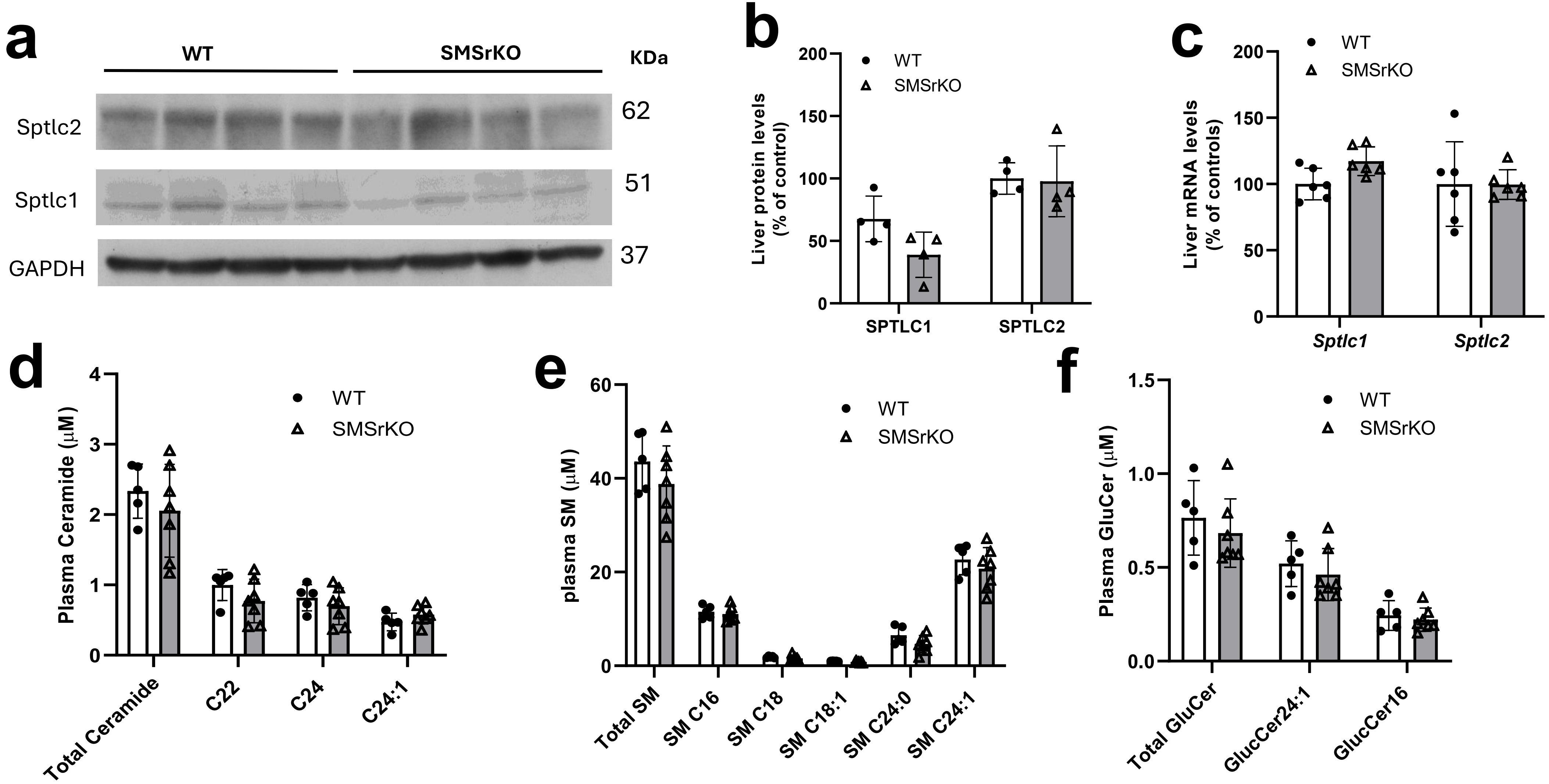

Fig. S2. *Smsr* Deficiency in Mice Shows No Significant Changes in Sphingolipid Levels Under a Normal Chow Diet . Four-month-old *Smsr* KO and WT mice on chow diet were utilized. Their livers and plasma were isolated. A, B) Western blots and corresponding quantification for liver SPTLC1 and SPTLC2. C) Real-time PCR analysis of liver *Sptlc1* and *Sptlc2*. D-F) Plasma sphingolipid measurements were measured by LC/MS/MS. Sphingolipid:C, ceramide; SM, sphingomyelin; GluCer, glucosylceramide. Values are mean ± SD, n =4-6, **P* < 0.01; **P< 0.05.
